# Resveratrol reduces the apoptosis induced by cigarette smoke extract by upregulating MFN2

**DOI:** 10.1371/journal.pone.0175009

**Published:** 2017-04-13

**Authors:** Chao Song, Bailing Luo, Li Gong

**Affiliations:** Respiratory Department, Xiangya Hospital, Central South University, Changsha, Hunan Province,China; Institute of Biochemistry and Biotechnology, TAIWAN

## Abstract

**Purpose:**

The aim of this study was to investigate the effect of resveratrol (RSV) on cigarette smoke extract (CSE)-induced cell apoptosis and mitochondrial dysfunction in vitro, as well as changes in the MFN2 expression level.

**Methods:**

Cultured human bronchial epithelial (HBE) cells were initially treated with CSE to induce apoptosis, followed by incubation either with or without RSV. Numerous techniques were used to evaluate the outcomes of the present study, including a cell counting kit-8 assay, real-time quantitative polymerase chain reaction (real-time qPCR), western blotting, JC-1 fluorescence, Hoechst 33342 staining, Annexin V-PI flow cytometry apoptosis analyses, and siRNA technology.

**Results:**

A 24 h incubation in 3.5% CSE induced apoptosis in HBE cells, and pretreatment of HBE cells with RSV (20 μM) significantly suppressed the CSE-induced apoptosis, prevented the CSE-induced decrease in MFN2 levels, suppressed BAX translocation to the mitochondria, and prevented mitochondrial membrane potential loss and cytochrome C release. However, following the transfection of MFN2 siRNA, the anti-apoptotic effects of RSV were significantly attenuated.

**Conclusion:**

The results of the present study demonstrated that RSV may protect bronchial epithelial cells from CS-induced apoptosis in vitro by preventing mitochondrial dysfunction, and MFN2 may be associated with the anti-apoptotic functions of RSV in HBE cells.

## Introduction

Chronic obstructive pulmonary disease (COPD) is the fourth leading cause of death in the world[1], and smoking is one of the most important causes of the disease. A large sample survey conducted in 2007 showed that the prevalence of COPD was 8.2% in the population over 40 years of age[2]. COPD pathogenesis is complex, and cell apoptosis has been a hot topic in the pathogenesis of COPD in recent years. Recent studies from around the world have found that, under the stimulation of cigarette smoke extract (CSE), abnormal mitochondrial morphology, the increase of mitochondrial fragments, and the dysfunction of mitochondria in cells can be observed[3–5]. Indeed, mitochondrial function is closely related to oxidative stress, inflammation, and apoptosis[6].

Mitochondria are dynamic organelles that continuously undergo fission and fusion, which is regulated by mitochondrial fusion and fission proteins, and their constant expression is a necessary condition for maintaining the stability of mitochondrial dynamics[7]. Mitochondrial fusion in mammals is mediated by the fusion proteins mitofusin 1 (MFN1), mitofusin 2 (MFN2) and optic atrophy 1 (OPA1). MFN1 and MFN2 are responsible for the fusion of the outer mitochondrial membranes. OPA1 is responsible for fusion of the inner mitochondrial membranes. Mitochondrial fission in mammals is mediated by dynamin-related protein 1 (Drp1), which interacts with four mitochondrial receptor proteins: fission 1 (Fis1), mitochondrial fission factor (Mff), mitochondrial dynamics protein of 49 kDa (MID49) and MID51. Severe stress can lead to mitochondrial fusion disorder; fusion is reduced, fission is increased, mitochondrial fragmentation is increased, the level of energy metabolism is decreased, ATP production capacity is decreased, the mitochondrial function is disordered, the self-repair capacity is decreased, and, finally, all these disorders induce oxidative stress and cell apoptosis. MFN2, as an important member of the mitochondrial fusion protein family, plays a key role in the process of mitochondrial fusion. Studies have shown that MFN2 also plays an important role in the occurrence and development of metabolic diseases, such as cardiovascular disease, cancer, and type 2 hereditary motor and sensory neuropathy (Charcot-Marie-Tooth, disease, CMT2)[8–10].

According to the related literature, when the MFN2 level is decreased, the expression of the apoptotic protein BAX can be increased; at the same time, BAX is located in the mitochondria, and the translocation of BAX can cause the release of cytochrome C. As a result, MFN2 deficiency potentially induces apoptosis[11]. It is known that the effect of cigarette on the function of mitochondrial fusion and fission is related to the effect of the fusion protein on the mitochondria. Cigarette smoke extract stimulates lung parenchymal cells, the expression of MFN2 is decreased, and Drp1 (mitochondrial fission protein) expression is increased, which causes an increase in mitochondrial fission fragments and results in human airway smooth muscle cell dysfunction[4, 12]. According to the above findings, the pathogenesis of mitochondrial dysfunction plays a role in apoptosis in COPD. It can be inferred that the downregulation of MFN2 might be involved in the process of apoptosis caused by mitochondrial dysfunction.

According to related articles, resveratrol (RSV) may protect bronchial epithelial cells from CS-induced apoptosis in vitro and in vivo by preventing mitochondrial dysfunction[13]. Vernon W. Dolinsky found that RSV can attenuate doxorubicin-induced cardiac injury in mice by stimulating the expression of MFN1 and MFN2[14]. At present, there are no studies on the effects of RSV on MFN2 in COPD; therefore, we hypothesized that RSV may inhibit the degradation of MFN2, promote the increase in mitochondrial fusion and enhance mitochondrial adaptation to prevent apoptosis under the stimulation of CSE. This study intended to explore whether, under CSE stimulation, RSV can inhibit apoptosis through a protective effect on mitochondrial function in human bronchial epithelial cells and provide a new theoretical basis for the prevention and treatment of COPD.

## Materials and methods

### Cell line and cell culture

Human bronchial epithelial (HBE) cells were obtained from Xiangya Central Laboratory (Bailing Luo, Changsha, Hunan province, China) and were cultured in a humidified incubator containing 95% air and 5% CO_2_ at 37°C in DMEM (Hyclone Laboratories, Inc., Logan, UT, USA) supplemented with 10% heat-inactivated fetal bovine serum (FBS; Hyclone Laboratories, Inc., Logan, UT, USA). The cells were detached for subculture using 0.25% trypsin (Gibco-BRL, Carlsbad, CA, USA). Once the cells reached 80% confluence, they were seeded into cell culture plates at the proper density, and grown to 80% confluence, prior to being used for further experiments.

### CSE preparation

CSE was prepared using a smoke machine as described by previous methods [15,16,17] with some modifications. The direct and side-stream smoke from a cigarette (Fu Rong brand, China) was directed via a tube through 5 ml of DMEM. The CSE solution was filtered through a 0.22 μm pore filter to remove bacteria and large particles. The concentration of CSE was calculated spectrophotometrically, measuring the optical density as previously described at a wavelength of 320 nm. The 5 ml solution was determined as 100% CSE and was diluted to obtain the desired concentration in each experiment. In all of the experiments, freshly prepared CSE was used.

### Preparation of RSV solution

RSV (purity >99%) was purchased from Sigma-Aldrich (Shanghai, China). A stock solution of 10^5^ μmol/l was made by dissolving 22.8 mg of RSV in 1 ml of dimethyl sulfoxide. We used different concentrations of the RSV (0 μM, 1 μM, 5 μM, 10 μM, 20 μM, 40 μM), and the final concentration of RSV used in the present study was 20 μmol/l.

### Cell viability assay

To determine a suitable concentration and duration for the CSE intervention, a cell counting kit-8 (CCK-8) assay was used to monitor cell viability. Briefly, HBE cells were seeded in 96-well culture plates with 8 × 10^3^ cells in 100 μl of culture medium per well. After allowing 24 h for cell attachment, the cultured HBE cells, at 80% confluence, were treated with CSE at various concentrations for 0, 6, 12, and 24 h at 37°C in an incubator containing 95% air and 5% CO_2_. A monosodium salt (WST-8) solution was added to each well, and the cells were further incubated at 37°C for 1 h in a 5% CO_2_ humidified incubator. A microplate reader was used to measure the absorbance at 450 nm. Each assay was performed in triplicate. The results of the treated groups were compared to the control group and represented as the percentage of the control value.

### Examination of apoptosis by Hoechst-PI staining and fluorescence imaging

To visualize the morphological changes of HBE cells during apoptosis, cell morphology was assessed using an Apoptosis and Necrosis Assay Kit (Beyotime Institute of Biotechnology). Cells in 6-well plates were washed twice with PBS (phosphate-buffered saline, pH 7.4). Then, 1 ml of assay buffer was added to each well, and the cells were stained with Hoechst 33342 (5 μl) and PI (5 μl) for 20–30 min at 4°C in the dark. The cells were then observed under a fluorescence microscope.

### Apoptosis detection with Annexin V/PI by flow cytometer

HBE cells were seeded in 6-well plates at a density of 3×10^5^ cells/well. Cells treated with drugs or the blank diluent were rinsed with warm PBS (37°C). The cell suspensions were washed twice with ice-cold PBS before further processing. The cells were stained using an Annexin V/PI staining kit (KeyGEN Annexin V-PE Apoptosis Detection Kit); 500 μl of binding buffer was added to each tube and transferred to a 1.5 ml centrifuge tube (1–5×10^5^ cells). Then, 5 μl of Annexin V–FITC and 5 μl of PI were added, and the cells were gently vortexed. Cells were then incubated for 15 min at room temperature (RT) in the dark. Finally, the cells were analyzed by flow cytometer after 1 h (Becton Dickinson) (Ex 488 nm, and Em 530 nm).

### Subcellular fractionation and western blot analysis

Cells were separately washed, collected, homogenized in a RIPA lysis buffer (10 mM Tris–HCl, pH 8, 0.32 mM sucrose, 5 mM EDTA, 2 mM dithiothreitol, 1 mM phenylmethyl sulfonylfluoride, and 1% Triton X-100), and centrifuged (12,000 rpm, 30 min, 4°C). The protein-containing supernatant was used to detect MFN2, BAX, and cytochrome C. To examine the subcellular location of BAX and cytochrome C, samples of cytosolic extracts were prepared according to the manual provided in the Cell Mitochondria Isolation Kit (Beyotime). To ensure that an equal amount of protein was loaded in each case, western blots were also performed using the BCA kit (Beyotime). Equal amounts of proteins (40 μg) were subjected to electrophoresis on a sodium dodecyl sulfate–polyacrylamide gel (8%-15%). The gel-separated proteins were transferred to polyvinylidine fluoride membranes (EMD Millipore, Billerica, MA, USA), and the membranes were blocked with 5% non-fat dry milk in TBST [10 mM Tris–HCl (pH 8.0), 137 mM NaCl, and 0.05% Tween-20 by vol] at RT (25°C) for 2 h and probed with primary antibodies (MFN2 1:1000, β-actin 1:2000, Cell Signaling Technology, USA; cytochrome C 1:1000, BAX 1:600, Abcam, USA) overnight at 4°C. Each of the targeted proteins was immunostained using these specific antibodies. The following day, the membranes were incubated with horseradish peroxidase-conjugated secondary antibodies (1:10,000 dilution; ZSGB-BIO, Beijing, China) in TBST. The blots were visualized using an enhanced chemiluminescence detection system (Advansta, Menlo Park, CA, USA).

### Real-time qPCR

Total RNA was isolated from HBE cells using the RNAiso Plus kit (Takara Biotechnology Co. Ltd., Dalian, China), according to the manufacturer's instructions. cDNA was synthesized from the RNA using the PrimeScript™ RT reagent kit with gDNA Eraser (Takara Biotechnology Co. Ltd., Dalian, China). Real-time qPCR was performed using SYBR®Premix Ex Taq^TM^ II (Tli RNaseH Plus) (Takara Biotechnology Co. Ltd., Dalian, China) on an iCycler (ABI ViiATM7; Applied Biosystems, Carlsbad, CA, USA). The thermocycler parameters were set as follows: step one, activation of the HotStartTaq DNA polymerase (Takara Biotechnology Co. Ltd.) at 95°C for 30 sec; step two, PCR was performed for 40 cycles with denaturation at 95°C for 5 sec and annealing at 60°C for 34 sec; step three, fixed parameters set by the ABI 7500 Fast Real-time PCR system (Applied Biosystems)-associated SDS software version 2.3 (Applied Biosystems). Data were quantitated with 2^−ΔΔCt^.

For each gene, an amplification curve was generated to evaluate the amplification efficiency. The sequences of the forward and reverse primers were: MFN2 forward 5'-*CAGGTGTAAGGGACGATTGG-*3' and reverse 5'-*CAAATGGGATGAAGCACTGA-*3'; GAPDH forward 5'-*CAAATGGGATGAAGCACTGA-*3' and reverse 5'-*CGTCAAAGGTGGAGGAGTG-*3'.

### Mitochondrial membrane potential (ΔΨm) analysis by JC-1 fluorescence

Cellular mitochondrial dysfunction can be reflected by the loss of the mitochondrial membrane potential, which can be indirectly measured by the fluorescent probe JC-1. JC-1 is a mitochondrial dye (5,5′,6,6′-tetrachloro-1,1,3,3′- tetraethylbenzimidazolylcarbocyanine chloride). The BD^TM^ MitoScreen Kit (BD Biosciences, USA) stains mitochondria in living cells in a membrane potential-dependent fashion. The JC-1 monomer is in equilibrium with J-aggregates that bind to the mitochondrial membrane. The monomer JC-1 fluoresces green (λ em = 527 nm), while the J-aggregates fluoresce red (λ em = 590 nm). Therefore, cells with normal mitochondrial membrane potential fluoresce orange. The depolarization of the mitochondria results in a decrease in the red component and, therefore, green fluorescence. The experiments were in strict accordance with the BD mitochondrial membrane potential detection kit. In summary, after the cells were trypsinized and washed twice with PBS, the cells were labeled with the fluorescent dye JC-1 for 30 min at 37°C. The excess dye was then removed with assay buffer, and the remaining cells were suspended in the buffer solution. The cells were then observed under a fluorescence microscope after 1 h.

### Effect of siRNA-mediated downregulation of MFN2 on the mitochondrial apoptosis of HBE cells

HBE cells were seeded in 6-well plates at a density of 3×10^5^ cells/well, and then transfected for 24 h with 50 nM of MFN2 siRNA (si-h-mfn2_001: *CGGCAAGACCGACTGAAAT*) and with 50 nM of negtive control RNAi (Ribobio Co., Ltd., Guangzhou, China), according to the riboFECTTMCP transfection reagent (Ribobio Co., Ltd., Guangzhou, Chia) instructions.

### Statistical analyses

Data are presented as the mean ± SE, and 3 experiments were performed in each case.Differences between two groups were assessed using two-tailed Student's t-test, and differences among three or more groups were assessed with one-way ANOVA with Tukey’s post hoc test. The tests were performed by GraphPad Prime 6 software (GraphPad, San Diego, CA, USA). Differences between groups were considered statistically significant when at least a 95% confidence level (p<0.05) was obtained.

## Results

### The influence of different concentrations of CSE for different durations on HBE cell viability

**Fig 1:**
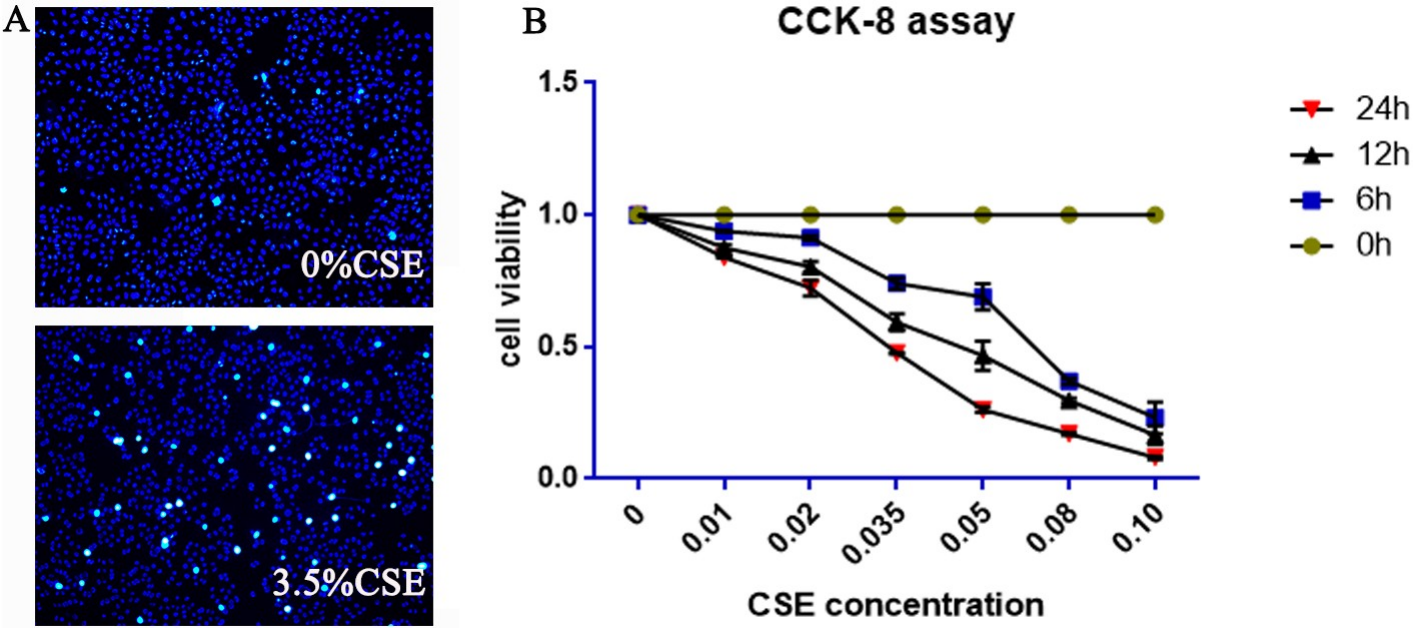
Effects of CSE on cell viability and apoptosis in HBE cells. (A) HBE cells undergoing 0% and 3.5% CSE exposure for 24 h showed typical characteristics of apoptosis, as detected by Hoechst 33342 staining. Apoptotic cells showed strong fluorescent signals (bright turquoise color) outlining the chromatin-condensed nuclei, whereas normal cells appeared weakly stained. (B) CCK-8 assay showed the influence of different concentrations of CSE at different times on HBE cell viability, and the 24 h of treatment with 3.5% CSE was the optimal choice. h, hours.

A CCK-8 kit was used to detect the cytotoxicity of CSE. Different concentrations of the CSE (0%, 1%, 2%, 3.5%, 5%, 8% and 10%) and different intervention time points (0, 6, 12, and 24 h) were tested. As the exposure time increased, the cell viability decreased. Thus, CSE affected cell viability in a dose-dependent manner. Cells were not significantly affected in terms of mortality or proliferation when exposed to 1% and 2% CSE. The cell viability after 24 h of exposure to 1% and 2% CSE were (83.9ÿ0.2)% and (72.3ÿ3)%, respectively. However, the cell viabilities after 24 h of exposure to 5%, 8% and 10% CSE were (26.2ÿ1)%, (17.5ÿ0.5)% and (8ÿ0.6)%, respectively. Thus, the viability was too low to perform further experiments. There was significant inhibition or toxic effects of 3.5% CSE on cells, and its 24 h cell viability was (47.7ÿ0.3)%; thus, 24 h of treatment with 3.5% CSE was chosen for the follow-up experiments. Detailed information can be found in Fig 1B.

### Effect of different concentrations of resveratrol (RSV) on MFN2 protein expression in HBE cells

**Fig 2:**
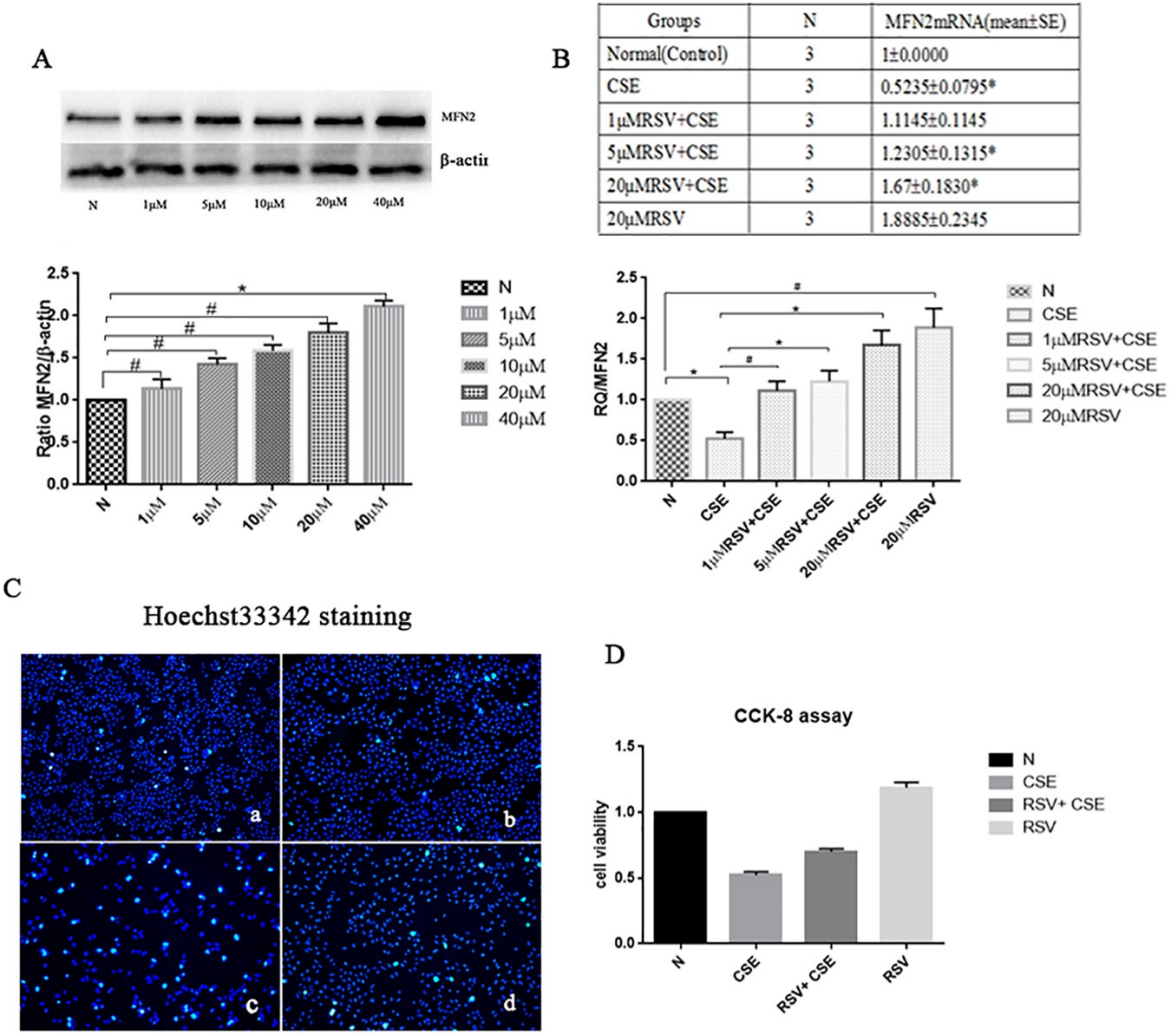
Effect of different concentrations of RSV on MFN2 protein expression in HBE cells and on the MFN2 mRNA levels in each group and the examination of apoptosis by Hoechst33342 staining fluorescence imaging as well as cell viability by CCK-8 assay. (A) With the increase of the RSV concentration, the expression of MFN2 increased, except for the 40 μM RSV group,there were no statistically significant differences in other RSV groups when compared with the control group (p>0.05). (B) The MFN2 mRNA level in the 5 μM RSV+CSE and 20 μM RSV+CSE groups was increased compared with that in the CSE group, and the 20 μM RSV+CSE group had a higher expression level than the 5 μM RSV+CSE group (p<0.05). However, the MFN2 mRNA level was not significantly affected by 1 μM RSV+CSE compared with that in the CSE group. (C) a; Control group; b: RSV group; c: CSE group; d: RSV+CSE group. Hoechst staining of HBE cells. HBE cells pretreated with RSV exhibited fewer apoptotic cells (d) than that of the non-RSV-treated group (c), following a 24 h exposure to 3.5% CSE. Apoptotic cells are strongly stained bright turquoise. (D) CCK-8 assay. The RSV+CSE group has a higher cell viability than CSE group. *: p<0.05, #: p>0.05.

HBE cells were exposed to different concentrations of RSV (0 μM, 1 μM, 5 μM, 10 μM, 20 μM, 40 μM) for 24 h. The western blotting results showed that with an increase in the concentration of RSV, the expression of MFN2 increased(Fig 2A). Except for the 40 μM RSV group,there were no statistically significant differences in other RSV groups when compared with the control group(p<0.05).

### MFN2 mRNA levels in HBE cells in each group were detected by real-time qPCR (means±SE)

In this study, the HBE cells were divided into four groups: 1) Control (Normal, N), HBE cells at 80% confluence cultured without CSE or RSV; 2) RSV, 20 μM RSV added to the cell culture medium; 3) CSE, HBE cells underwent 3.5% CSE exposure for 24 h; and 4) RSV+CSE, HBE cells were precultured with 1 μM, 5 μM, or 20 μM RSV for 2 h, followed by a 24 h exposure to 3.5% CSE. The MFN2 mRNA level in the CSE group was decreased compared with that in the control groups, and the difference was statistically significant (p<0.05). The MFN2 mRNA levels of the 5 μM RSV+CSE and 20 μM RSV+CSE groups were increased compared with that in the CSE group, and the 20 μM RSV+CSE group had a higher level than the 5 μM RSV+CSE group (p<0.05); however, the MFN2 mRNA level was not significantly affected by 1 μM RSV+CSE compared with that in the CSE group. These results indicated that within a certain concentration range(0 μM–20 μM), RSV can help increase MFN2 mRNA level, which can be decreased by the CSE.Thus, 24 h of treatment with 20 μM RSV and 3.5% CSE was chosen for the follow-up experiments.

### Examination of apoptosis by Hoechst–PI staining fluorescence imaging and cell viability by CCK-8 assay

When cells undergo apoptosis, chromatin condensation occurs (Fig 1A). Hoechst 33342 can penetrate the cell membrane, and the fluorescence of apoptotic cells is obviously enhanced compared to that of normal cells. As seen in Fig 2C, the control group (Fig 2C.a) and RSV group (Fig 2C.b) cells exhibited uniform Hoechst 33342 staining, and there was a low level of fluorescence-enhanced cells and many normal cells. By contrast, the number of cells treated by CSE (Fig 2C.c) was less than that of the normal group, the staining was uneven, and the blue fluorescence was enhanced, representing typical apoptotic cells. The number of apoptotic cells in the CSE+RSV group (Fig 2C.d) was less than that of the CSE group. From the Fig 2D, we can see the RSV+CSE group has a higher cell viability than CSE group, and the differe -nce was statistically significant (p<0.05). These results showed that RSV has protective effects on HBE cells treated with CSE.

### Western blot analysis to test the protein expression of MFN2, release of cytochrome C from mitochondria to cytosol and BAX translocation in HBE cells

#### MFN2 protein expression levels

**Fig 3:**
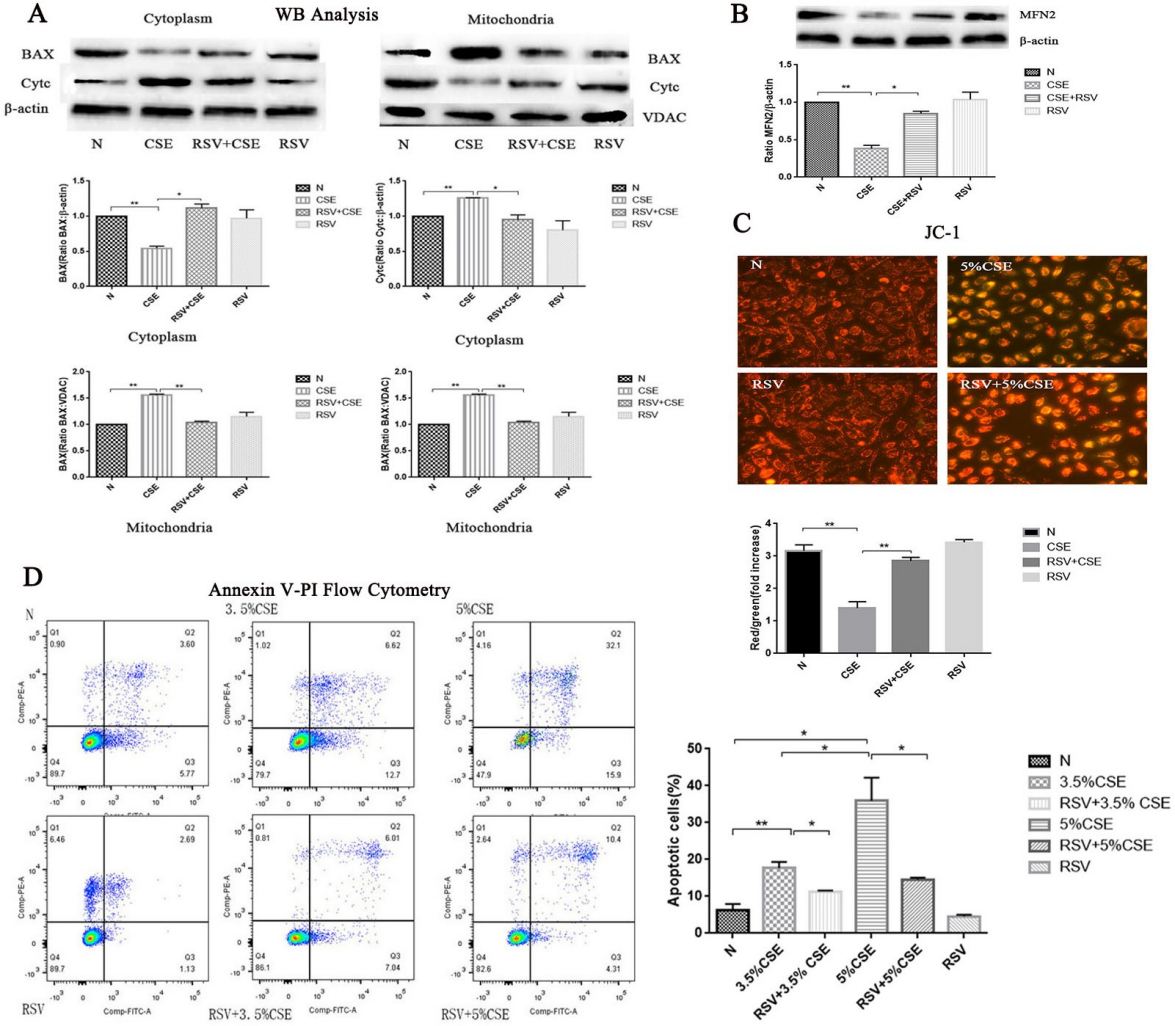
The protective effects of RSV against CSE-induced apoptosis and the activity of MFN2 and its preventative effects of the release of cytochrome C from mitochondria to cytosol and BAX translocation. (A) BAX began to translocate to mitochondria in the CSE group, cytochrome C in the cytoplasm began to increase in the CSE group compared with the normal group (p<0.05). However, RSV pretreatment prevented the release of cytochrome C from the mitochondria to cytosol and prevented the translocation of the BAX from the cytosol to mitochondria. (B) MFN2 levels were decreased in the CSE group compared with those in the control group (p<0.05). However, RSV pretreatment prevented the decrease of MFN2 expression (p<0.05). (C) Mitochondrial membrane potential (ΔΨm) analysis by JC-1 fluorescence. In the CSE group, ΔΨm levels were lower than those in the control groups (p<0.05). However, ΔΨm was significantly increased in the RSV+CSE group compared with the CSE group (p<0.05).

We determined the expression of MFN2 by western blotting to assess whether the MFN2 levels were different among the four groups. As shown in Fig 3B, the MFN2 levels were decreased in the CSE group compared with those in the control group (p<0.05). However, RSV pretreatment prevented the decrease in the MFN2 level (p<0.05).

#### The release of cytochrome C from the mitochondria to the cytosol and BAX translocation

We investigated the effects of RSV on the CSE-induced subcellular distribution of BAX and cytochrome C. The levels of BAX and cytochrome C in the cytosolic and mitochondrial fractions were determined by western blotting. As shown in Fig 3A, BAX began to translocate to mitochondria in the CSE group, and cytochrome C in the cytoplasm began to increase in the CSE group compared with that in the normal group (p<0.05). However, RSV pretreatment prevented the release of the cytochrome C from the mitochondria to the cytosol and prevented the translocation of BAX from the cytosol to mitochondria.

### Mitochondrial membrane potential (ΔΨm) analysis by JC-1 fluorescence

We assessed ΔΨm using the JC-1 (Fig 3C) probe. In the CSE group, the ΔΨm levels were lower than those in the control groups. However, a difference in the levels was noted between the two groups; ΔΨm was significantly increased in the RSV+CSE group compared with the CSE group. These results suggest that RSV inhibits CSE-induced apoptosis by affecting mitochondrial function through energy metabolism.

### Apoptosis detection with Annexin V/PI by flow cytometry

At 24 h, CSE exposure resulted in typical apoptotic changes in the cells compared with cells from the other groups, and the 5% CSE group had higher apoptotic rates than those of the 3.5% group (Fig 3D). RSV+CSE groups remarkably lessened the severity of apoptosis compared with the CSE groups, which was confirmed by Annexin V-PI flow cytometry.

### MFN2 siRNA attenuates the protective effects of RSV against CSE-induced apoptosis in HBE cells

**Fig 4:**
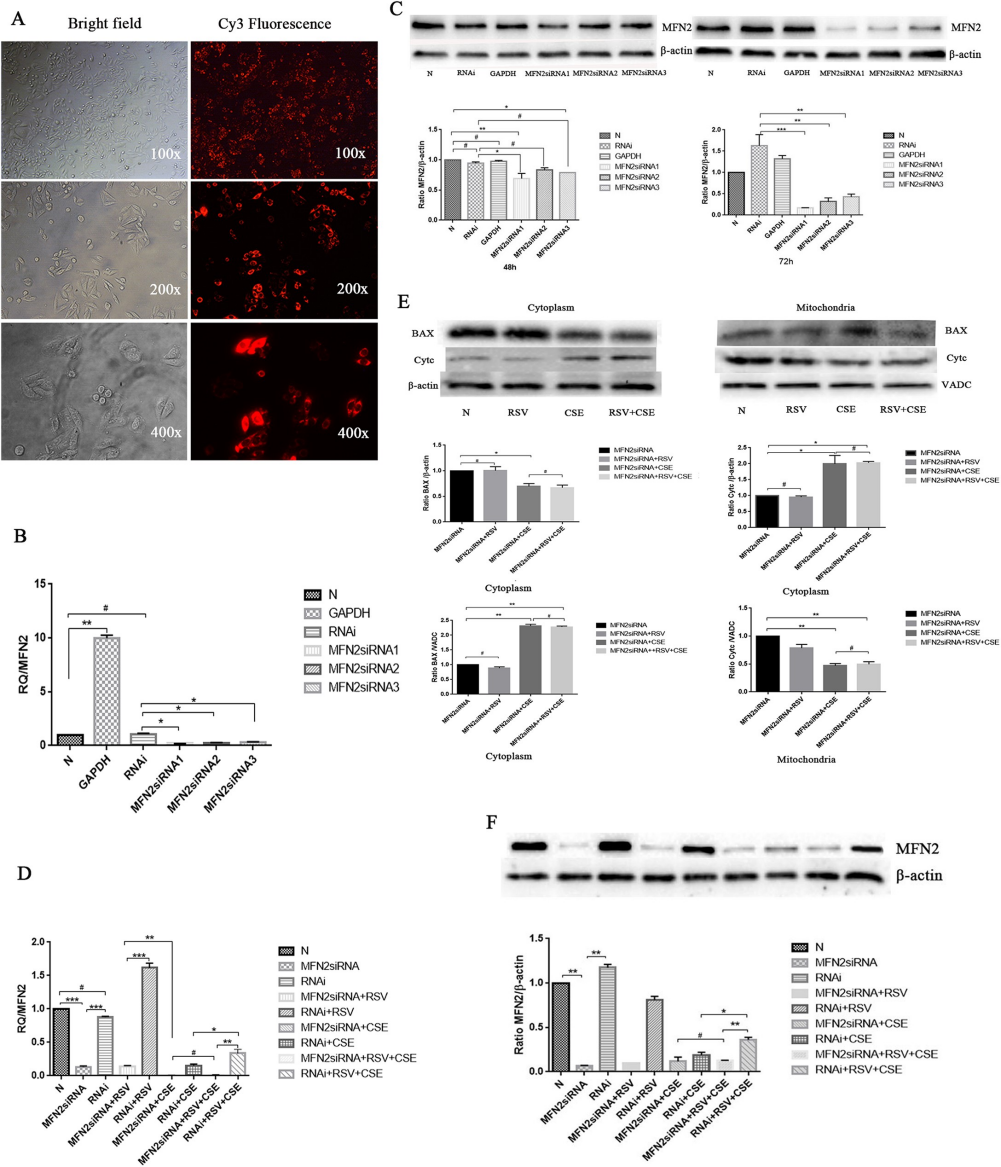
MFN2 siRNA attenuates the protective effects of RSV against the apoptosis induced by CSE in HBE cells. (A) The transfection efficiency of HBE cells was detected by fluorescence microscopy, which showed that more than 90% of cells were labeled (red fluorescence). (B-C) Real-time qPCR and western blot analysis were used to test three MFN2 RNA interference target sequences for gene silencing efficiency. The results show that the first silence sequence (si-h-mfn2_001: *CGGCAAGACCGACTGAAAT*) exhibited the highest efficiency, and the optimal intervention time was 72 h. Therefore, the first RNA interference target sequence was selected for the following experiments. (D) CSE-induced apoptosis was markedly attenuated by RSV pretreatment (RNAi + RSV + CSE); however, this protective effect was abolished by MFN2 gene knockdown (MFN2 siRNAi+ RSV + CSE). (E) Western blot analysis showed apoptosis marker molecules in all of the groups. BAX began to translocate to mitochondria in the CSE group, and cytochrome C in the cytoplasm began to increase in the CSE group compared with that in the MFN2 siRNA group (p<0.05). However, the effects of RSV to prevent the release of the cytochrome C from the mitochondria to the cytosol and prevent the translocation of the BAX from the cytosol to the mitochondria were decreased. There were no differences between the MFN2 siRNA+ CSE group and the MFN2 siRNA+ RSV+CSE group (p>0.05). (F) Western blot analysis showed that the RSV pretreatment did not increase the MFN2 levels in the MFN2 siRNA groups compared with the negative control siRNA groups. *: p<0.05, **: p<0.01, ***; p<0.001, #: p>0.05.

**Fig 5:**
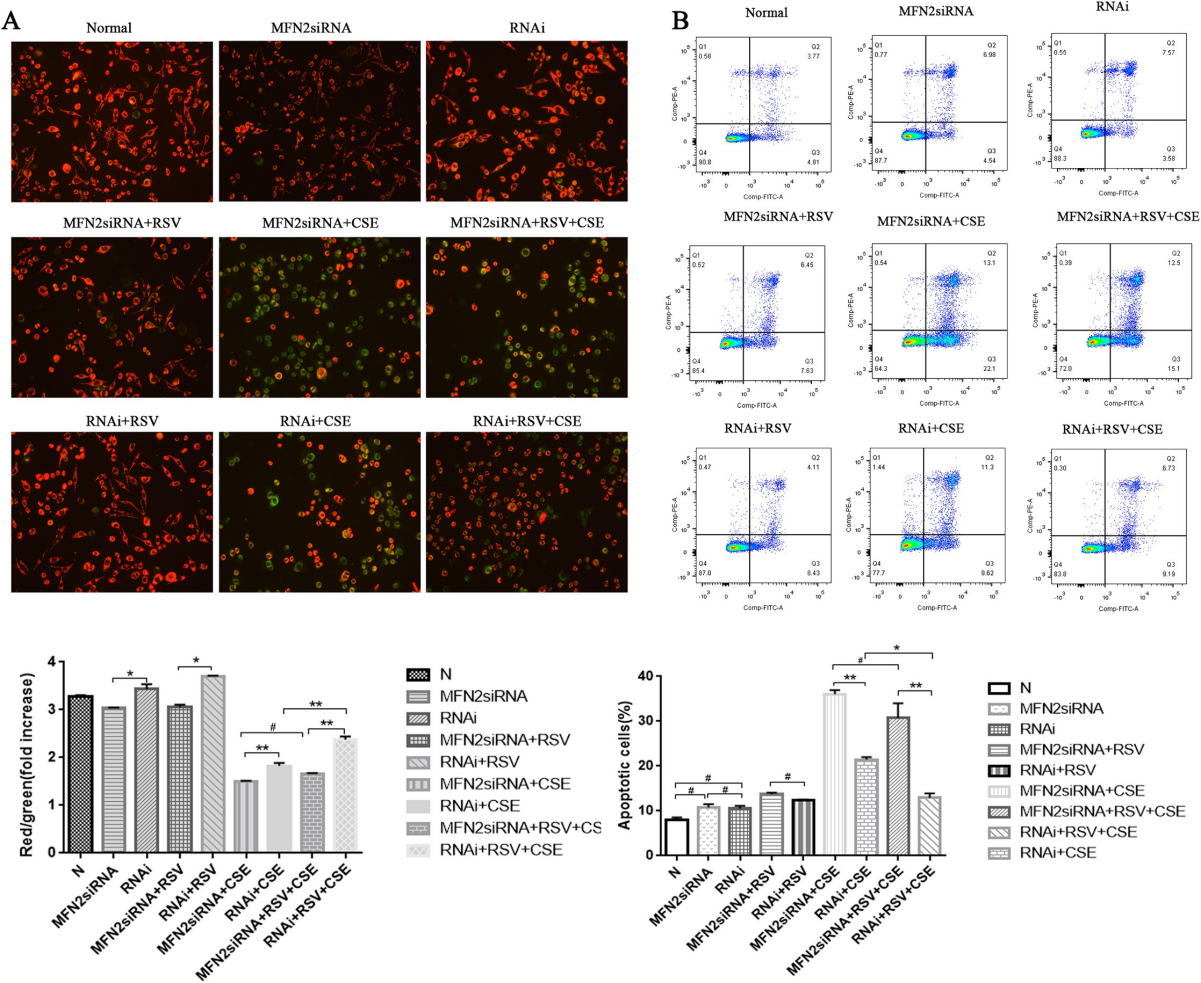
The protective effects of RSV were markedly reduced following the siRNA-mediated knockdown of MFN2 compared with those in the negative RNAi groups, as shown in the MFN2 siRNA+RSV+CSE group and the RNAi+RSV+CSE group. (A-B) As for the MFN2 siRNA groups, an increased number of apoptotic cells was detected, together with a much higher apoptotic rate than the control RNAi groups (B), which was consistent with the changes in the mitochondrial membrane potential (ΔΨm) analyzed by JC-1 fluorescence (A). Group 9 had a lower red/green rate than group 8. *:p<0.05, **:p<0.01, #:p>0.05.

To determine whether MFN2 is associated with the protective effects of RSV against CSE-induced apoptosis in HBE cells, MFN2 siRNA transfection was performed (Figs 4 and 5). The transfection efficiency of HBE cells was detected by fluorescence microscopy (Fig 4A), which showed that more than 90% of cells were labeled (red fluorescence). Real-time qPCR and western blot analysis were used to test three MFN2 RNA interference target sequences for optimal gene silencing efficiency. The results showed that the first silence sequence (si-h-mfn2_001: *CGGCAAGACCGACTGAAAT*) exhibited the highest efficiency, and the optimal intervention time was 72 h. Therefore, the first RNA interference target sequence was selected for the following experiments (Fig 4B and 4C). HBE cells were divided into nine groups: 1) Control (Normal, N), HBE cells at 80% confluence cultured without any treatment for 24 h; 2) MFN2 RNAi, MFN2 siRNA-transduced HBE cells without RSV or CSE; 3) MFN2 RNAi+RSV, MFN2 siRNA-transduced HBE cells pre-cultured with 20 μM RSV; 4) RNAi, negative control siRNA-transduced HBE cells without RSV or CSE; 5) RNAi + CSE, negative control siRNA-transduced HBE cells exposed to 3.5% CSE for 24 h; 6) RNAi + RSV, negative control siRNA-transduced HBE cells precultured with 20 μM RSV; 7) MFN2 RNAi + CSE, MFN2 siRNA-transduced HBE cells exposed to 3.5% CSE for 24 h; 8) RNAi + RSV + CSE, negative control siRNA-transduced HBE cells pre-cultured with 20 μM RSV for 2 h, followed by a 24 h exposure to 3.5% CSE; and 9) MFN2 RNAi + RSV + CSE, MFN2 siRNA-transduced HBE cells precultured with 20 μM RSV for 2 h, followed by a 24 h exposure to 3.5% CSE.

The protective effects of RSV were still present in the cells that were transfected with the negative control siRNA and underwent CSE exposure (group 8). However, following the transfection of HBE cells with the specific MFN2 siRNA (group 9), the protective effects of RSV were markedly attenuated (Figs 4 and 5). The cells that were pre-incubated with control siRNA showed no effect on MFN2 expression, implying that the successful downregulation of MFN2 was performed in HBE cells (Fig 4D and 4F). Compared with group 9, the cells in group 8 showed marked durability against CSE-induced apoptosis when the cells were transfected with the negative control siRNA and exhibited downregulation of the mitochondrial apoptotic markers cytochrome C release and BAX translocation (Fig 4E), thus implying that the protective effects of RSV on the cells may be achieved by the expression of MFN2. The apoptotic cell rate in group 8 was 15.92%, compared with 20.92%, 35.2%, and 27.6% in groups 5, 7, and 9, respectively (Fig 5B). However, the protective effects of RSV were markedly reduced following the siRNA-mediated knockdown of MFN2, as shown in group 9. An increased number of apoptotic cells was detected (Fig 5B), which was consistent with the changes in the mitochondrial membrane potential (ΔΨm) analyzed by JC-1 fluorescence (Fig 5A) and a marked upregulation of the mitochondrial apoptotic markers cytochrome C release and BAX translocation (Fig 4E). There was no statistically significant difference between the MFN2 siRNA+CSE group and the MFN2 siRNA+RSV+CSE group (p>0.05).

These results indicate that MFN2 is necessary for RSV to exert its anti-apoptotic effects in the CSE apoptosis cell model. However, the mechanisms by which RSV influences MFN2 expression remain unclear.

## Discussion

COPD pathogenesis is complex, and CS is an important contributor to the incidence of COPD. Apoptosis induced by CSE is one of the significant mechanisms of COPD. Mitochondria play a major role in apoptosis. Mitochondrial dysfunction in cell apoptosis is characterized by a decrease in the mitochondrial membrane potential, increased production of ROS, respiratory impairment and the release of apoptogenic proteins, including cytochrome C and apoptosis-inducing factor (AIF)[13,18,19]. Mitochondrial membrane potential loss plays a central role in the initiation of apoptosis and is linked to the release of cytochrome C from the mitochondria to the cytoplasm[20, 21]. CS can cause a variety of types of cell oxidative damage. Several lines of evidence demonstrate that CS exposure induces mitochondrial morphology abnormalities, mitochondrial fragmentation, and mitochondrial dysfunction. In conclusion, the function of mitochondria is closely related to oxidative stress, inflammation, and apoptosis. HBE cells are the first line of defense against external pathogens in the respiratory tract. Excessive apoptosis of the epithelial cells of the airways and defective repair processes are hallmarks of COPD[22]. Our previous study indicated that RSV plays a protective role in HBE cell apoptosis induced by CSE[17, 23], but the exact mechanism needs to be further discussed.

MFN2 is a mitochondrial protein that controls mitochondrial fusion and tethering. When the level of MFN2 is decreased, dysfunction of mitochondrial fusion occurs, mitochondrial membrane fragments are increased, and the ions inside and outside of the mitochondria are imbalanced. As a result, the mitochondrial membrane potential is disrupted, and BAX translocates to the mitochondria in response to stimulation[13]. Mitochondrion-bound BAX is associated with the loss of the mitochondrial membrane potential and the release of cytochrome C from the mitochondrial inter-membrane space[13].

In our previous study, we demonstrated that RSV inhibited apoptosis in CSE-treated HBE cells and CS-exposed mouse lungs[17,23]. Here, we confirmed that RSV inhibited apoptosis in CSE-treated HBE cells in vitro and also attenuated CS-induced apoptosis in vitro by improving mitochondrial function. In this study, we observed that CSE can decrease MFN2 expression and thereby affect the mitochondrial membrane potential. However, RSV can improve mitochondrial function by upregulating MFN2 expression.

Our data that support a role for MFN2 is consistent with recent findings demonstrating that MFN2 can interact with the Bcl family proteins[24]. First, MFN2 affects the mitochondrial recruitment of BAX and the release of cytochrome C during cell apoptosis. When the MFN2 level is increased, cytochrome C is released from the mitochondria to the cytosol and BAX translocation is decreased, and vice versa.

When we used CSE to stimulate HBE cells transfected with MFN2 siRNA, there were more apoptotic cells in the low MFN2 expression group than that of the negative control group. Even when the cells were pretreated for 2 h with RSV, the cell protection and mitochondrial membrane potential improvement of low MFN2-expressing cells were not significant compared to the negative control group cells. In addition, the inhibiting levels of the release of cytochrome C and BAX translocation of the negative control group were higher than the low MFN2-expressing cells.

## Conclusions

In conclusion, the protective effect of RSV on HBE cells may occur by increasing MFN2 expression, improving mitochondrial function, inhibiting BAX translocation and the release of cytochrome C, and inhibiting cell apoptosis caused by the mitochondrial apoptosis pathway. This study provides a new theoretical basis for the prevention and control of COPD; we can increase MFN2 expression or inhibit its degradation to improve mitochondrial fusion function. Further studies will be needed to investigate the mechanism of how RSV interacts with MFN2. According to our preliminary work and other published literature[17,25,26], we know that RSV may exert protective effect against CSE-induced apoptosis by increasing SIRT1 and ORP150 expression in HBE cells, and ORP150 can inhibit endo-plasmic reticulum (ER)-stress(ERS); the mitochondria and ER have extensive contacts, and MFN2 maybe one of the important molecular briges which mediate the contacts. Therefore, we speculate that the effect of RSV on MFN2 may be associated with SIRT1[27,28] and ORP150, so the further research will be performed around the relationship between MFN2 and SIRT1/ORP150.

## Supporting information

S1 Fig CCK-8. (TIF)

S2 Fig JC-1. (TIF)

S3 Fig flow cytometer. (TIF)

S1 Text Resveratrol exerts an anti-apoptotic effect on human bronchial epithelial cells undergoing cigarette smoke exposure. (PDF)

S2 Text Endoplasmic reticulum–mitochondria contacts: function of the junction. (PDF)

S3 Text ER Tubules Mark Sites of Mitochondrial Division. (PDF)

S4 Text Vam3, a resveratrol dimer, inhibits cigarette smokeinduced cell apoptosis in lungs by improving mitochondrial function. (PDF)
