# Correction: Resveratrol reduces the apoptosis induced by cigarette smoke extract by upregulating MFN2

**DOI:** 10.1371/journal.pone.0213877

**Published:** 2019-03-12

**Authors:** Chao Song, Bailing Luo, Li Gong

After publication of this article [1], concerns were raised about Fig 3.

In Fig 3A, the "BAX" band in the Cytoplasm group is identical to the "Cytc" band in the Mitochondria group. Since the BAX in the cytoplasm is similar to the Cytc trend in the mitochondria, the authors mistakenly placed the band representing the BAX trend in cytoplasm in the position that represents the mitochondrial Cytc.

In the cell experiment, the authors repeated each experimental method at least three times. The experiments were repeated at different times and the results of the three experiments were consistent with each other. The authors have provided the raw, underlying images for Fig 3A from all experimental repeats. The original data of Fig 3A are included in S1 File.

The authors note that there is another error in Fig 3A. The second bar graph for Mitochondria is a duplicate of the first bar graph and the cytochrome C graph is missing. The bar graph of “Cytc in the mitochondria” was mistakenly replaced by the bar graph of “BAX in the mitochondria” in Fig 3A, resulting in the duplication of BAX in the mitochondria picture.

Please see corrected Fig 3 here.

**Fig 3:**
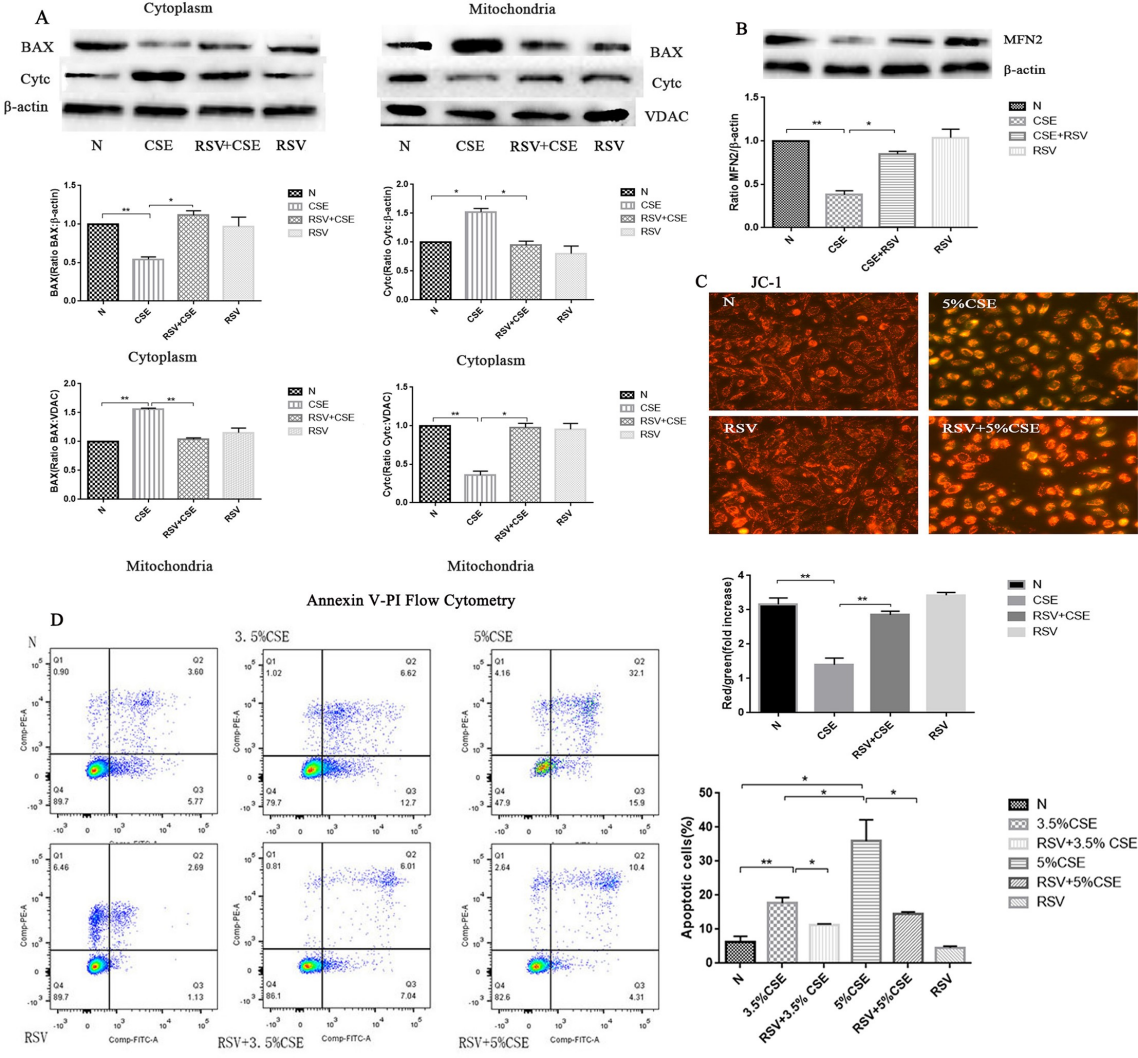
The protective effects of RSV against CSE-induced apoptosis and the activity of MFN2 and its preventative effects of the release of cytochrome C from mitochondria to cytosol and BAX translocation. (A) BAX began to translocate to mitochondria in the CSE group, cytochrome C in the cytoplasm began to increase in the CSE group compared with the normal group (p<0.05). However, RSV pretreatment prevented the release of cytochrome C from the mitochondria to cytosol and prevented the translocation of the BAX from the cytosol to mitochondria. (B) MFN2 levels were decreased in the CSE group compared with those in the control group (p<0.05). However, RSV pretreatment prevented the decrease of MFN2 expression (p<0.05). (C) Mitochondrial membrane potential (ΔΨm) analysis by JC-1 fluorescence. In the CSE group, ΔΨm levels were lower than those in the control groups (p<0.05). However, ΔΨm was significantly increased in the RSV+CSE group compared with the CSE group (p<0.05).

Primary data underlying all other figures can be found in S2 File.

An Academic Editor reviewed the updated figure(s) and underlying data and confirmed that they support the results and conclusions reported in the published article.

## Supporting information

S1 File Raw figures underling Fig 3. Raw data for mitochondria bax, Cytc and cytoplasm bax and Cytc.
(ZIP)

S2 File Supplementary raw data. Raw data for Fig 1B, Fig 2A and 2B, Fig 3A, 3B, 3C and 3D, Fig 4B–4F, Fig 5A and 5B.
(ZIP)
