# Supplementary figures and images for: Resveratrol reduces the apoptosis induced by cigarette smoke extract by upregulating MFN2
Source: PLoS One. 2017 Apr 13;12(4):e0175009. doi: 10.1371/journal.pone.0175009 (PMC5391199; doi:10.1371/journal.pone.0175009)

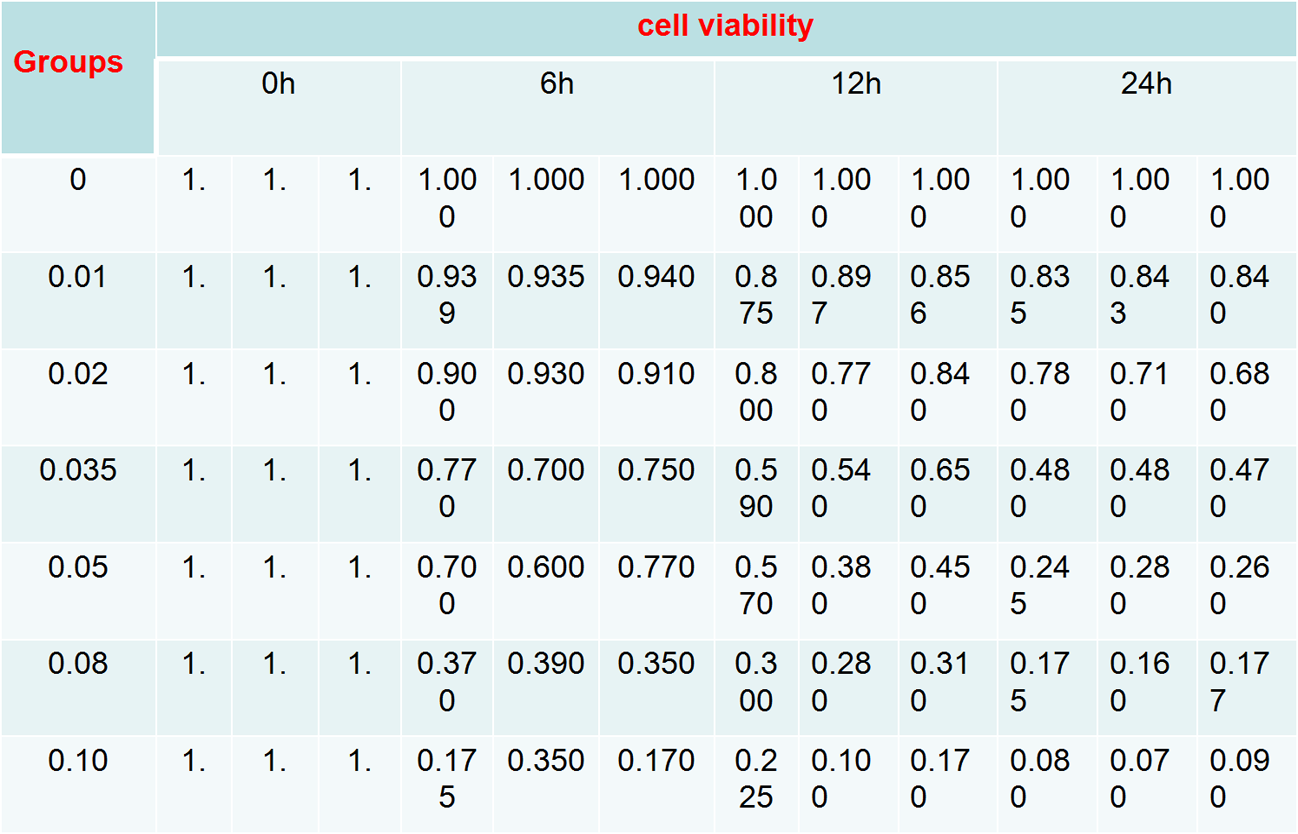

Supplement: S1 Fig — (TIF) [file pone.0175009.s001.tif]

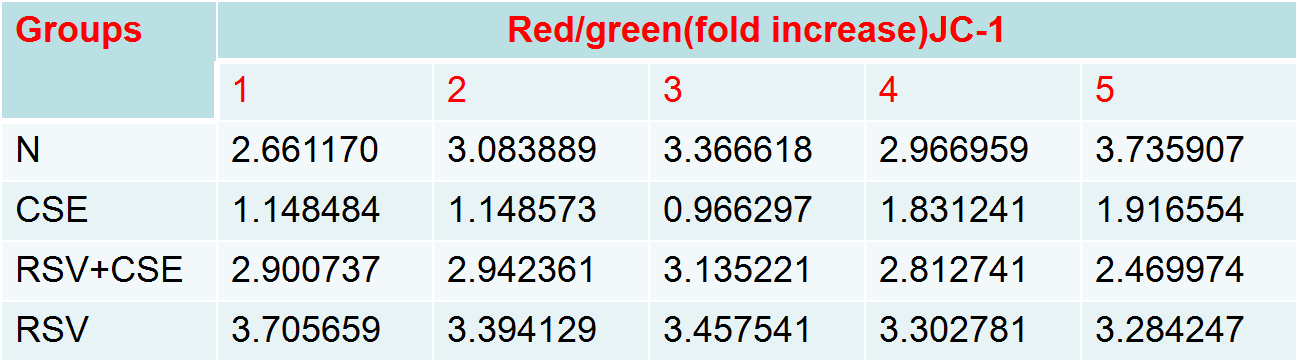

Supplement: S2 Fig — (TIF) [file pone.0175009.s002.tif]

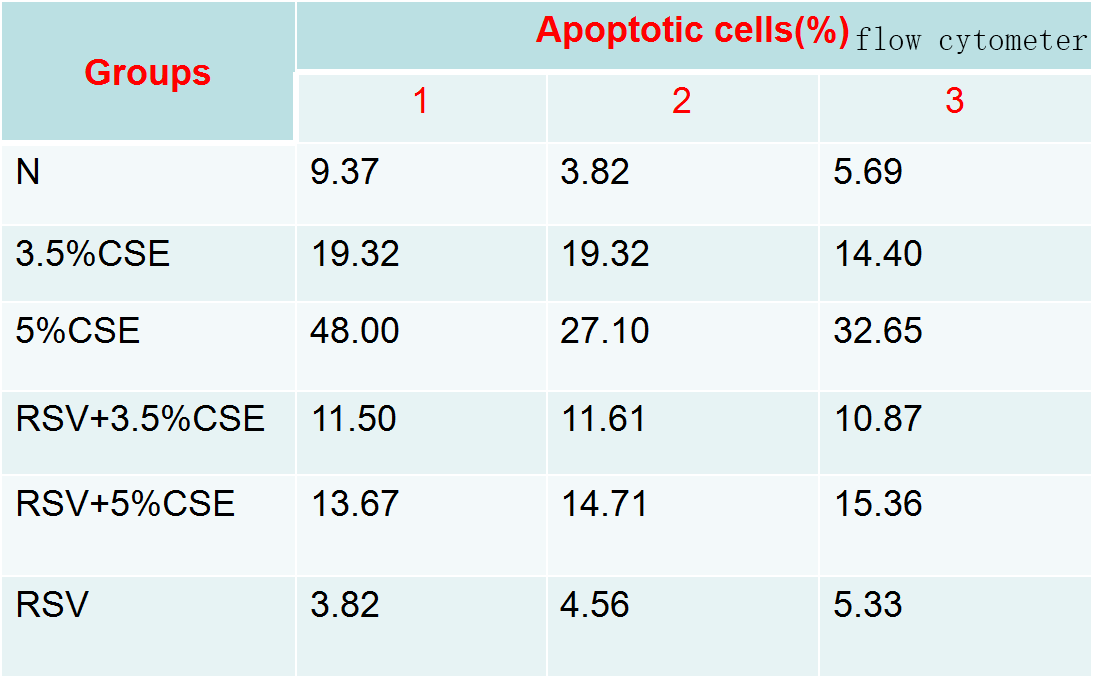

Supplement: S3 Fig — (TIF) [file pone.0175009.s003.tif]
