# Supplementary material for: Resveratrol reduces the apoptosis induced by cigarette smoke extract by upregulating MFN2
Source: PLoS One. 2017 Apr 13;12(4):e0175009. doi: 10.1371/journal.pone.0175009 (PMC5391199; doi:10.1371/journal.pone.0175009)
Supplement: S2 Text — (PDF) [file pone.0175009.s005.pdf]

Endoplasmic reticulum–mitochondria contacts: function of the junction

Ashley A. Rowland and Gia K. Voeltz

Abstract | The most well-characterized organelle contact sites are those between the endoplasmic reticulum (ER) and mitochondria. Increased understanding is being gained of how ER–mitochondria contact sites are organized and which factors converge at this interface, some of which may provide a tethering function. The role of the ER–mitochondria junction in coordinating the functions of these two organelles is also becoming clearer, and it has been shown to be involved in the regulation of lipid synthesis, Ca^{2+} signalling and the control of mitochondrial biogenesis and intracellular trafficking.

Department of Molecular, Cellular, and Developmental Biology, University of Colorado, Boulder, Colorado 80309, USA.
Correspondence to G.K.V.
e-mail:
gia.voeltz@colorado.edu
doi:10.1038/nrm3440
Published online
20 September 2012

The cytoplasm of eukaryotic cells is partitioned into membrane-bound organelles in order to compartmentalize and concentrate specialized functions within, or on, the membrane surfaces. The largest of the membrane-bound organelles is the endoplasmic reticulum (ER). Many important cellular functions are carried out by the domains of the ER. It is the site where luminal and secreted proteins, as well as membrane proteins, are synthesized and then translocated so that they can undergo trafficking to secretory and endocytic compartments. The ER is also an intracellular storage site for Ca^{2+} and holds most of the biosynthetic enzymes that are involved in the synthesis of cellular lipids. In the past few decades, it has become apparent through the use of high-resolution microscopy that the ER is not isolated but rather forms contact sites with many other cytoplasmic organelles, including the mitochondria, Golgi, peroxisomes, endosomes, lysosomes and lipid droplets, as well as the plasma membrane (for reviews, see REFS 1,2). The identity of organelles is based on their resident proteins and the specific functions that only they perform. Therefore, the existence of contact sites between organelles suggests that the factors that are localized to two different organelles can come together and synergize additional functions at these specialized domains.

The most well-characterized organelle contact sites are those between the ER and mitochondria. Here we discuss the organization of ER–mitochondria contacts and the factors that converge at this interface, some of which may provide a tethering function. We also emphasize the emerging role of the ER–mitochondria junction in coordinating the functions of these two organelles, including the part it plays in regulating lipid synthesis, Ca^{2+} signalling and controlling mitochondrial biogenesis and intracellular trafficking.

Structure of ER–mitochondria contacts

Regions of close contact between the ER and mitochondrial membranes can be observed by electron microscopy and fluorescence microscopy in animal cells and yeast (FIG. 1A–C). Contact sites are defined as regions where two membranes are closely apposed but the membranes do not fuse and thus the organelles each maintain their identities. The contact sites between the ER and mitochondria have been measured to be 10–30 nm wide[3,4]. This distance is close enough to suggest that the two organelles are tethered together by proteins located on the apposing membranes. Ribosomes are also excluded from the ER membrane at contact sites, which further indicates that contact sites form at specialized ER domains[3,4]. Contact sites can have different structural features. Some contact sites are discrete, whereas others are more extensive. For example, in some cases ER tubules circumscribe almost completely around the mitochondrial membrane[4] (FIG. 1A). Contact sites also appear to be stable structures because the two organelles stay tethered to each other even as they move along the cytoskeleton[5]. Live cell imaging shows that the two organelles can traffic in a coordinated fashion without any noticeable disruption in their contact[5] (FIG. 1D,E). This perseverance of the tight linkage between these organelles despite their dynamics suggests that maintained contact is important. Multiple functions that occur at contact sites are being characterized; whether these each occur at separate specialized contact domains or whether they occur synergistically through a common domain has yet to be determined and will be discussed here.

Functions of ER–mitochondria contacts

Stable contact sites between the ER and mitochondria provide an opportunity to synergize the functions of the two organelles. It has now become clear that these

© 2012 Macmillan Publishers Limited. All rights reserved

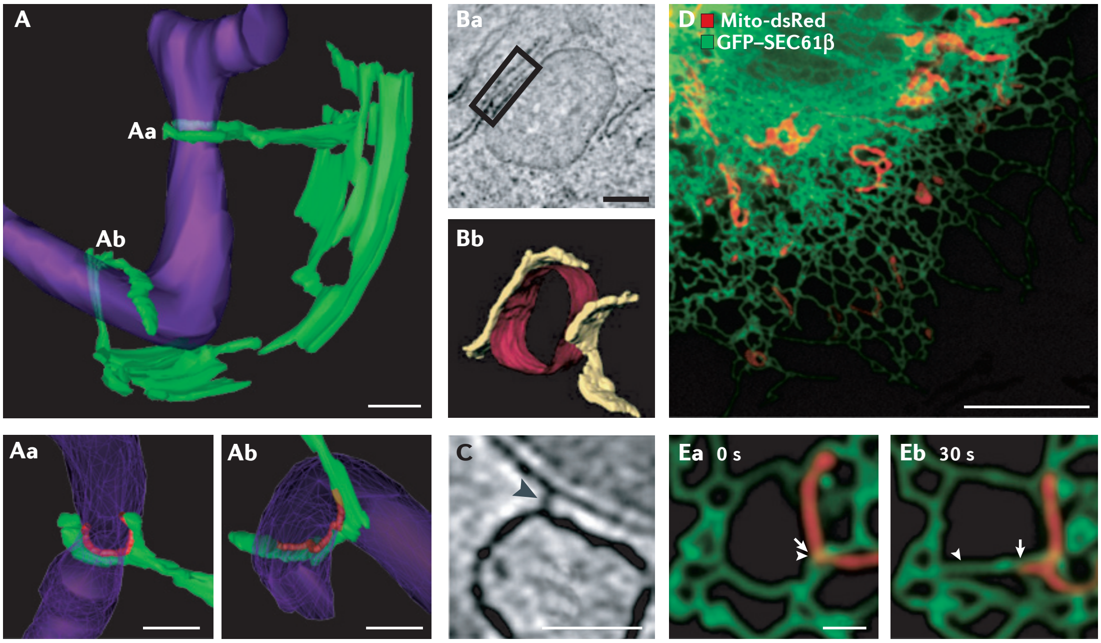

Figure 1 | **Structure and dynamics of ER–mitochondria contact sites.** **A** | A three-dimensional (3D) electron microscope (EM) tomogram reveals contact sites (**Aa** and **Ab**) between the endoplasmic reticulum (ER; green) and a mitochondrion (purple) in a wild-type yeast cell. The mitochondrial membrane is constricted at position **Aa** where it is 'clamped' by an ER tubule. Higher-magnification images of contact sites **Aa** and **Ab** are shown in the bottom panels. Regions of contact are marked in red and are defined as points where the apposed membranes are within 30 nm of each other and free of ribosomes on the ER. **B** | An EM tomograph (**Ba**) and corresponding three-dimensional tomogram (**Bb**) of contact domains between the mitochondria (red) and the ER (yellow) in an inositol 1,4,5-trisphosphate receptor (*IP3R*) triple-knockout DT40 cultured chicken cell. The box in **Ba** shows a region of contact. **C** | An EM tomograph of a rat liver cell reveals electron-dense 'tethers' between the ER and mitochondrial membrane (marked by an arrowhead). **D** | A confocal fluorescent image of a Cos-7 cell labelled with mito-dsRed (showing the mitochondria in red) and green fluorescent protein (GFP)–SEC61β (showing the ER in green). **E** | A higher-magnification image of mitochondria and ER imaged live as in **D** at two time points. Arrows indicate the position of ER (arrowhead) and mitochondria (arrow) movement from 0 to 30 seconds. As the mitochondria moves, the ER moves with it. Scale bars represent 200 nm in **A**, 250 nm in **B**, 50 nm in **C**, 10 μM in **D** and 1 μM in **E**. Images in **A** are reproduced, with permission, from REF. 4 © (2011) American Association for the Advancement of Science. Images in **B** and **C** are reproduced, with permission, from REF. 3 © (2006) Rockefeller University Press. Images in **D** and **E** are reproduced, with permission, from REF. 5 © (2010) Rockefeller University Press.

contacts can allow regulation of one organelle by the other, as well as concerted regulation of cell biological processes through bidirectional trafficking of factors between the two organelles. Here we discuss four main functions that have been characterized for ER–mitochondria contacts, including control of lipid biosynthesis, mitochondrial division, Ca^{2+} signalling and coordinated dynamics of the two organelles. In each case, we discuss what is known about the factors that localize to these contacts and may orchestrate these functions.

Lipid exchange during biosynthesis. Most of the enzymes involved in lipid biosynthesis are localized to the ER membrane; however, some are located on the mitochondrial membrane. In some cases, the enzymes required for synthesis of a single phospholipid are located on both the ER and the mitochondria. Thus, there are lipid biosynthetic pathways that are thought to utilize ER–mitochondria contact sites. Biochemically, a fraction of the ER can be isolated that is attached to mitochondria (referred to as the mitochondria-associated membrane (MAM)); this fraction is enriched in enzymes that are involved in lipid synthesis, including phosphatidylserine (PS) synthase[6–8]. In fact, biosynthesis of two of the cell's most abundant phospholipids, phosphatidylcholine (PC) and phosphatidylethanolamine (PE), is coordinated by largely uncharacterized molecular complexes at the ER–mitochondria interface[9] (FIG. 2a). During this biosynthetic process, PS is first made by enzymes on the ER, but it must be translocated to the outer mitochondrial membrane (OMM) and then transferred again to the inner mitochondrial membrane (IMM), where the enzymes are located that convert it to PE[10]. To make PC, the PE precursor must then be translocated from the OMM to the ER, where it is modified by ER enzymes to make PC. There must also be a mechanism by which PC is translocated back from the ER to the OMM, as mitochondria also contain PC. Clearly, the lipid exchange between the two membranes is bidirectional and extensive, although the mechanism for exchange and the factors involved in lipid transport remain elusive. It is interesting to consider how biosynthesis and lipid transfer between these two membranes could be regulated in order to maintain the steady-state ratios of phospholipids found in each of these organelles.

© 2012 Macmillan Publishers Limited. All rights reserved

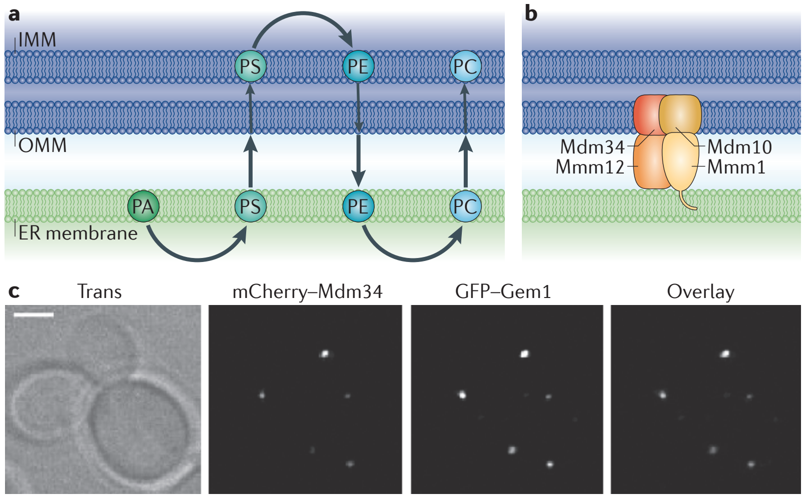

Figure 2 | **ER–mitochondria contact sites mediate lipid biosynthesis.** **a** | The biosynthesis pathway that generates phosphatidylcholine (PC) from phosphatidic acid (PA) requires sequential steps in both the endoplasmic reticulum (ER) and mitochondria. The enzymes that convert PA to phosphatidylserine (PS) or phosphatidylethanolamine (PE) to PC are in the ER, whereas the enzymes that convert PS to PE are in the mitochondria. So, to achieve the final lipid composition of either membrane, there must be a significant exchange of lipids between the two organelles. **b** | The ER–mitochondria encounter structure (ERMES) is a complex in yeast consisting of proteins that reside in both the ER and outer mitochondrial membranes (OMMs). ERMES forms a bridge between the ER and mitochondrial membranes: maintenance of mitochondrial morphology protein 1 (Mmm1) localizes to the ER; mitochondrial distribution and morphology protein 10 (Mdm10) and Mdm34 are in the OMM; and Mmm12 is cytoplasmic. **c** | The ERMES component Mdm34 (labelled with mCherry) localizes to punctate structures on mitochondria that colocalize with Gem1 (GTPase EF-hand protein of mitochondria 1). GFP, green fluorescent protein; IMM, inner mitochondrial membrane. Images in **c** are reproduced, with permission, from REF. 12 © (2011) National Academy of Sciences.

An ER–mitochondria tethering complex has been identified in a yeast screen that may coordinate phospholipid synthesis between the two membranes. This synthetic screen aimed to identify mutants that disrupted ER–mitochondria tethering and whose phenotype could be rescued by an artificial tether. It revealed a four-member complex, the ER–mitochondria encounter structure (ERMES), which consists of maintenance of mitochondrial morphology protein 1 (Mmm1), mitochondrial distribution and morphology protein 10 (Mdm10), Mdm12 and Mdm34 (REF. 11) (FIG. 2b). All four components colocalize at punctate structures on the mitochondria[11,12] (FIG. 2c). Subsequent studies have made a compelling case that ERMES is a tether. Not only can ERMES defects be rescued by a synthetic tether but ERMES components also include both ER (Mmm1) and OMM (Mdm10 and Mdm34) proteins, although Mdm12 is cytoplasmic[12] (FIG. 2b). Furthermore, Mmm1, Mdm12 and Mdm34 belong to a group of seven yeast proteins that share a synaptotagmin-like mitochondrial-lipid binding protein (SMP) domain, which may be important for their localization at the ER–mitochondria junction[13]. Indeed, deletion of the SMP domain from the ERMES component Mmm1 prevents its accumulation at contact sites[13]. The SMP domain is predicted to belong to the tubular lipid-binding (TULIP) protein superfamily. TULIP family members have an affinity for lipids, and some members are known to be involved in lipid trafficking, a function that is also carried out at the ER–mitochondria junction[14]. Cells with defective members of the ERMES complex have a lower rate of PS conversion to PC than wild-type cells, indicating that ERMES may be important for coupling at sites of lipid exchange[11]. However, others have reported no significant effect of ERMES component deletions on PS to PE conversion and suggest that ERMES could be a tether for other functions that occur at ER–mitochondria contact sites[15]. This discrepancy could be due to the use of different methods for lipid analyses, or it is possible that PE to PC conversion is the step during biosynthesis that is defective in the absence of functional ERMES[11,15]. It is agreed, however, that ERMES is a strong candidate in yeast for a physical tether between the ER and mitochondria. Future work will be needed to determine how many functions at ER–mitochondria contacts require the ERMES tether. Notably, an animal homologue of ERMES has not yet been identified, so another tethering complex must exist in animal cells to allow phospholipids to be transferred between the ER and mitochondria.

ER control of mitochondrial biogenesis. ER–mitochondria contact is maintained despite the fact that mitochondrial morphology is continuously being altered by mitochondrial fission and fusion. Mitochondrial division is driven by dynamin-related protein 1 (DRP1) in vertebrates (the yeast orthologue of which is Dnm1). DRP1 is a cytoplasmic protein that is recruited to the mitochondrial membrane, where it circumscribes the OMM as a helical oligomer. Fission occurs as DRP1 hydrolyses GTP, causing a conformational change in the oligomer that clenches the membrane and triggers fission[16–19]. A long-standing question has been what recruits this division machinery from the cytosol to a specific position along the mitochondrial membrane. In yeast, mitochondria fission 1 protein (Fis1) and mitochondrial division protein 1 (Mdv1) are required to recruit Dnm1 from the cytosol to the mitochondrial membrane[20–22]. However, the vertebrate orthologues of Fis1 are expendable, and there is no orthologue for Mdv1 (REF. 23). DRP1 instead depends on the OMM protein mitochondrial fission factor (MFF) for its recruitment to the mitochondrial membrane[23,24]. Thus, a conserved complex on the OMM that recruits DRP1 to fission sites has not yet been identified. Notably, sites of ER tubule contact with the mitochondrial membrane correlate with localization of DRP1 (REF. 4) (FIG. 3a). In vertebrate cells, these ER tubules circumscribe the mitochondrial membrane at constriction sites marked by DRP1 and its cofactor MFF[4] (FIG. 3a). In fact, DRP1 and MFF make excellent live fluorescent markers for ER–mitochondria contact sites. ER–mitochondria contact is not disrupted by DRP1 or MFF depletion, which suggests that contact is independent of division machinery recruitment[4]. ER tubules similarly mark the position of Dnm1 recruitment in yeast (FIG. 3a). Contact with the ER is therefore a conserved feature of mitochondrial division sites. This contact is also maintained after fission, and this may be possible because ER tubules are coupled specifically with the site of mitochondrial division.

© 2012 Macmillan Publishers Limited. All rights reserved

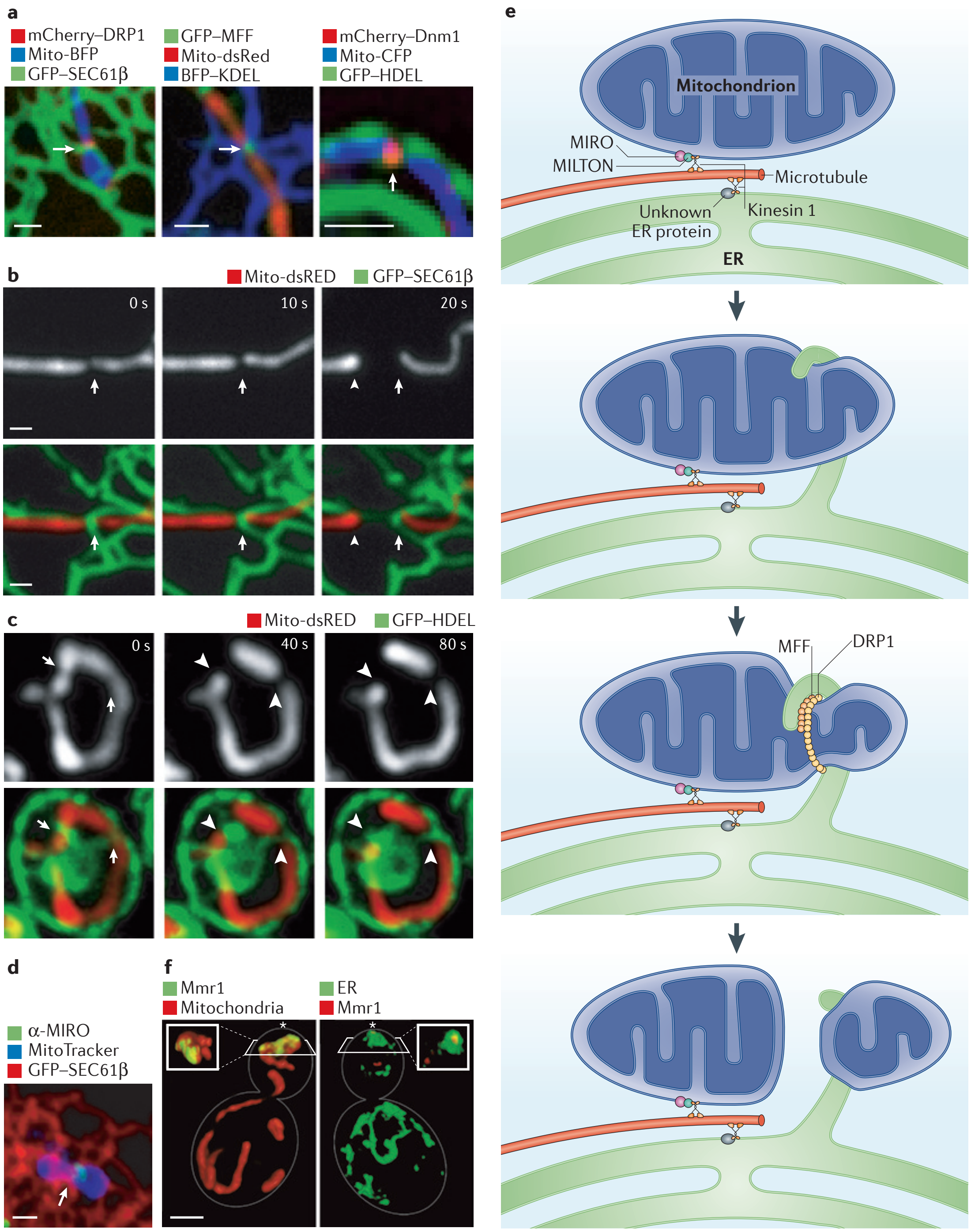

The ER not only marks the site of division machinery recruitment but also marks the positions where mitochondria are constricted for extended periods of time before division[4] (FIG. 3b,c). As mean mitochondrial diameters far exceed the diameter of the helix formed by DRP1, it has been proposed that something aside from dynamin family members must first constrict mitochondria[16,24,25]. Indeed, the demonstration that ER contacts circumscribe mitochondrial constriction sites suggests that the ER might drive initial constriction of mitochondria before the division machinery is recruited. Consistent with this, depletion of either DRP1 or the factor that recruits DRP1, MFF, prevents mitochondria from undergoing normal fission, resulting in an elongated morphology. But the mitochondria are still constricted at positions where the ER tubules circumscribe the mitochondrial membrane[4]. Thus, the ER is located at mitochondrial constrictions even before MFF and DRP1 recruitment.

Still, the causal relationship between ER contact and mitochondrial constriction has not yet been established. One possibility is that the ER does not cause the mitochondrial constriction but simply associates with these sites: the ER might probe the mitochondrial surface until

© 2012 Macmillan Publishers Limited. All rights reserved

◀ Figure 3 | **ER–mitochondria contacts are important for mitochondrial dynamics.** **a** | Confocal microscopy images showing the mitochondrial division machinery proteins DRP1 (dynamin-related protein 1) and MFF (mitochondrial fission factor) in Cos-7 cells and the yeast division machinery dynamin Dnm1 localizing to positions where endoplasmic reticulum (ER) tubules circumscribe the mitochondrial membrane. **b**,**c** | Live confocal fluorescence time-lapse images of Cos-7 cells (**b**) and yeast cells (**c**) showing mitochondrial constriction followed by division taking place at the site of an ER tubule crossing the mitochondria. Arrows indicate the initial site of constriction and arrowheads indicate the site of mitochondrial division. **d** | The Ca^{2+} binding protein MIRO (mitochondrial Rho GTPase), which regulates mitochondrial motility, localizes to a position (indicated by an arrow) where an ER tubule crosses over the mitochondrial membrane in a Cos-7 cell. **e** | A model of multiple factors that converge at ER–mitochondria contact sites to regulate mitochondrial dynamics. Both mitochondria and the ER are linked to microtubules by factors that associate with the microtubule motor kinesin 1. In the case of mitochondria, this occurs through kinesin 1 binding to the cytoplasmic protein MILTON, which in turn binds MIRO on the outer mitochondrial membrane. MIRO is important for mitochondrial movement. Less is known about the ER proteins that tether the ER to microtubules. Mitochondrial constriction mediated by DRP1 occurs at sites of ER–mitochondria contact, and one possibility is that ER contact promotes initial constriction of the mitochondria before DRP1 recruitment. Ultimately, mitochondrial fission is promoted by DRP1 and its cofactor MFF. **f** | Volume rendering of structured illumination microscopy images show a yeast cell during coordinated organelle inheritance into the bud. This is mediated by contact sites between the ER and the mitochondria, and normal ER inheritance is important for mitochondrial inheritance. Mitochondrial MYO2 receptor-related protein 1 (Mmr1) links the mitochondria to the ER during inheritance and accumulates between the mitochondria (shown in red) and the cortical ER (labelled by green fluorescent protein (GFP)–Sec63, in green). The bud tip is indicated by an asterisk. Scale bars represent 1 μM. Images in **a**–**c** are reproduced, with permission, from REF. 4 © (2011) American Association for the Advancement of Science. The image in **d** is reproduced, with permission, from REF. 12 © (2011) National Academy of Sciences. Images in **f** are reproduced, with permission, from REF. 48 © (2011) Elsevier. BFP, blue fluorescent protein; CFP, cyan fluorescent protein; HDEL, His-Asp-Glu-Leu; KDEL, Lys-Asp-Glu-Leu.

it finds a region with the amount of membrane curvature that indicates a constriction. Alternatively, the ER might actually promote constriction by physically wrapping around and squeezing the mitochondria at contact sites (FIG. 3e). As lipid biosynthesis also occurs at ER–mitochondria contact sites, it seems plausible that the domains of lipid asymmetry generated at these contacts could change the shape of the OMM and IMM in a way that drives constriction. Regardless of the mechanism used, it is clear that protein complexes localized to the OMM at the ER–mitochondria interface must be required to recruit the factors that regulate mitochondrial division.

It is possible that mitochondrial fusion could also be influenced by contact with the ER. In mammalian cells, both mitofusin 1 (MFN1) and MFN2 are known to tether two mitochondria together to direct their fusion[26]. The MFN2 protein also tethers contacts between mitochondria and ER[27], an unusual quality that requires its localization to both organelles. In mouse embryonic fibroblasts lacking MFN2, ER–mitochondria contact is reduced but can be rescued by expression of an MFN2 construct that contains an ER-targeting sequence[27]. This demonstrates that the presence of MFN2 on the ER can restore tethering to MFN1 on the mitochondria. What is not clear is whether MFN2 is unique in its ability to promote tethering or whether it is one of many tethers that exist in animal cells; the latter is more likely, since MFN2 depletion does not affect ER tethering at constriction sites in mammalian cells[4]. Considering that MFN2 affects both mitochondrial fusion and ER–mitochondria tethering, an appealing possibility is that ER contact is also required for fusion and MFN2 is the tether at these sites.

Regulating mitochondrial dynamics and inheritance. Both the ER and mitochondria are highly dynamic organelles capable of undergoing numerous reorganizations while maintaining a consistent overall shape. Live confocal microscopy reveals that the two organelles remain tethered to each other even as they move[5] (FIG. 1D,E). Both organelles move bidirectionally on microtubules in animal cells using a mechanism that requires the motor proteins kinesin 1 and dynein[28,29]. How the tethered organelles coordinate their movements along the cytoskeleton so that they are not ripped apart also remains an interesting question with few answers. In animal cells, it has been demonstrated that the two organelles colocalize over a population of microtubules that are post-translationally modified by acetylation[5]. This could be one mechanism to ensure that they track together at least along the same microtubule. Alternatively, one organelle might be dominant during dynamic movements and simply drag the other organelle with it.

The ER protein (or proteins) that tethers the dynamic ER to motor proteins on microtubules has not yet been identified. Mitochondrial movement is better understood. In animal cells, the most well-characterized complex that regulates mitochondrial movement includes the central player MIRO (mitochondrial Rho GTPase). MIRO is an OMM protein that binds to a cytoplasmic factor, MILTON, which in turn binds kinesin 1 heavy chain on microtubules[30] (FIG. 3d). MIRO is both a Ras-like GTPase and a Ca^{2+} binding protein that contains two EF-hand motifs that sense increases in cytosolic Ca^{2+}. Increased cytoplasmic Ca^{2+} causes mitochondria to stop moving on microtubules, and this effect can be suppressed when MIRO is depleted or a MIRO EF-hand mutant is expressed[31]. MIRO is thus proposed to be a Ca^{2+} sensor that stops mitochondrial movement when Ca^{2+} levels increase.

Contacts between ER and mitochondria seem to be important for regulating the dynamics of mitochondria. Intriguingly, immunofluorescence microscopy analysis shows that the OMM protein MIRO1, which regulates mitochondrial dynamics, localizes to punctae that correspond well to positions of ER–mitochondria contact[12] (FIG. 3d). Thus, a likely possibility is that direct sensing of Ca^{2+} release from the ER at contact sites by MIRO either blocks mitochondrial motility or dissociates mitochondria from microtubules at certain contact sites. Ca^{2+} release from the ER could be part of the mechanism that allows ER and mitochondrial movements to be coordinated along microtubules. It is also interesting that high Ca^{2+} levels lead to activation of DRP1, which increases mitochondrial fission[31]. This Ca^{2+}-dependent effect on DRP1 requires MIRO, and MIRO depletion also increases mitochondrial division[31]. Notably, MIRO is highly conserved, and its yeast homologue, Gem1 (GTPase EF-hand protein of mitochondria 1), colocalizes with ERMES punctae. In a similar way to loss of MIRO,

© 2012 Macmillan Publishers Limited. All rights reserved

loss of Gem1 causes mitochondria to become globular or fragmented[11,12,32]. Gem1 has also been reported to biochemically associate with the ERMES complex[12,33]. Thus, there are strong links between ER contact sites and MIRO and Gem1, as one or both of these factors have been linked to mitochondrial motility, Ca^{2+} sensing, ERMES and DRP1-mediated mitochondrial division.

The ER and mitochondria also have coupled dynamics in yeast, and the mechanisms by which this occurs have been best studied during yeast cell division. The ER and mitochondria cannot be generated *de novo*; therefore, both must be properly segregated to the growing daughter cell bud. Both organelles are inherited through the bud neck and orient along the mother–bud axis on actin cables attached to the bud tip[34–36]. In the case of the ER, it first extends through the bud neck along this central axis, and then branches out to re-establish the cortical ER, the peripheral ER domain that is closely apposed to the plasma membrane at a mean distance of 33 nm[37]. ER inheritance into the bud requires that it move in a polarized fashion. In yeast, both the ER and mitochondria do not move on microtubules but instead track on actin filaments using myosin motors. Both ER and mitochondria depend on two different type-V myosin family members for their polarized inheritance: the ER uses myosin 4 (Myo4)[36,38,39], whereas mitochondria use Myo2 (REFS 40–46). Nevertheless, the two organelles appear to maintain contact as they are inherited.

The ER is inherited into the bud before mitochondria[21,37,42], and ER contact is required to direct mitochondrial dynamics towards the bud. When cortical ER inheritance is blocked by disruption of the Myo4 motor, mitochondrial inheritance is defective despite the fact that Myo4 does not regulate actin-dependent movement of mitochondria[39]. A member of the DSL family of tethering proteins, Mmr1 (mitochondrial MYO2 receptor-related protein 1), links mitochondria to the ER during inheritance. Mmr1 associates with mitochondria and Myo2, and thereby acts as a linker protein[32,40–43,47]. Mmr1 deletion does not affect the normal dynamics of mitochondria, but disrupts the ability of these mitochondrial movements to be directed towards the bud[48]. Its localization is consistent with this function: it concentrates in punctae at the leading edge of mitochondria that have been pulled into the bud tip[47]. These punctae have been shown by three-dimensional confocal microscopy to be precisely positioned at contact sites between the mitochondrial membrane and the apposing membrane of the cortical ER (FIG. 3f). So, Mmr1 tethering could effectively prevent mitochondria from 'backtracking' into the mother cell.

Proper ER and mitochondrial distribution during inheritance in yeast also requires Ypt11, a Rab-like protein that localizes to the cortical ER. In the absence of Ypt11, there are defects in the inheritance of both ER and mitochondria to the bud tip[42,48]. Ypt11 binds to the tail of Myo2, and its overexpression leads to increased mitochondrial accumulation in the bud. Ypt11 overexpression can also compensate for the loss of Mmr1, consistent with a role for Mmr1 in directly mediating mitochondrial transport into the bud[48]. Mutations in the ERMES tethering complex also cause a defect in mitochondrial but not ER inheritance[11,15,49–51]. Similarly to Mmr1 loss, the defect in inheritance caused by compromising the ERMES complex can also be rescued by Ypt11 overexpression[15]. Together, these data demonstrate that ER contact is important for mitochondrial inheritance in budding yeast. They also raise the possibility that this contact could affect mitochondrial migration during other processes that require polarized cell growth. For example, it will be interesting to determine whether this contact has a role during axon generation and degeneration.

It is unclear whether ER contact also anchors mitochondria during animal cell division. The ER undergoes structural reorganization in animal cells during mitosis, but it remains controversial whether the ER becomes more cisternal or tubular[52–55]. Mitochondria also undergo structural changes during the cell cycle. At the onset of mitosis, DRP1 is phosphorylated by cyclin B–cyclin dependent kinase 1 (CDK1) and mitochondria become fragmented[56]. DRP1 is also recruited to the mitochondrial membrane, a process that is dependent on phosphorylation of the small G protein RALA by the mitotic kinase Aurora A and accumulation of RALA-binding protein 1 (RALBP1)[57]. Knockdown of either RALA or RALBP1 results in elongation of mitochondria during mitosis[57]. It is not known whether the ER and mitochondria remain tightly coupled throughout mitosis or whether the dramatic shape changes that occur in both organelles result in their dissociation.

Coordinating Ca^{2+} transfer. Ca^{2+} is released from the ER to mitochondria at contact sites, and this seems to be important for mitochondrial function, division and regulation of apoptosis[58,59] (FIG. 4a). This Ca^{2+} release is proposed to occur through the ER Ca^{2+} channel inositol 1,4,5-trisphosphate receptor ($Ins(1,4,5)P_3R$) to the voltage-dependent anion-selective channel protein 1 (VDAC1) on the OMM[59,60]. Although these factors have not yet been shown to partition specifically to contact sites by fluorescence microscopy, they are biochemically enriched in the same MAM membrane fraction that contains enzymes involved in lipid biosynthesis[61]. Using an $InsP_3$ agonist, Ca^{2+} transfer to mitochondria has been shown to be $Ins(1,4,5)P_3R$-dependent; agonist addition increases Ca^{2+} efflux from the ER and promotes Ca^{2+} uptake into mitochondria[58]. Furthermore, use of a Ca^{2+} sensitive photoprotein, aequorin, in the mitochondrial matrix or in the intermembrane space (IMS) has demonstrated that $Ins(1,4,5)P_3R$-induced Ca^{2+} release leads to localized sites of Ca^{2+} influx in the IMS[59], suggesting that there may be localized subdomains of Ca^{2+} transfer between the ER and mitochondria. Further support for these microdomains has come from GFP-based Ca^{2+} probes that localize to the cytosolic surface of the OMM and selectively monitor $[Ca^{2+}]$[62]. Elegant studies have also shown that artificial tethers between the ER and mitochondria can be used to alter the efficiency of Ca^{2+} transfer[3,63]. In particular, separate fusion proteins were used that localize to either the mitochondria or the ER and form a covalent linkage upon addition of rapamycin (FIG. 4b). Rapamycin-induced artificial tethering

© 2012 Macmillan Publishers Limited. All rights reserved

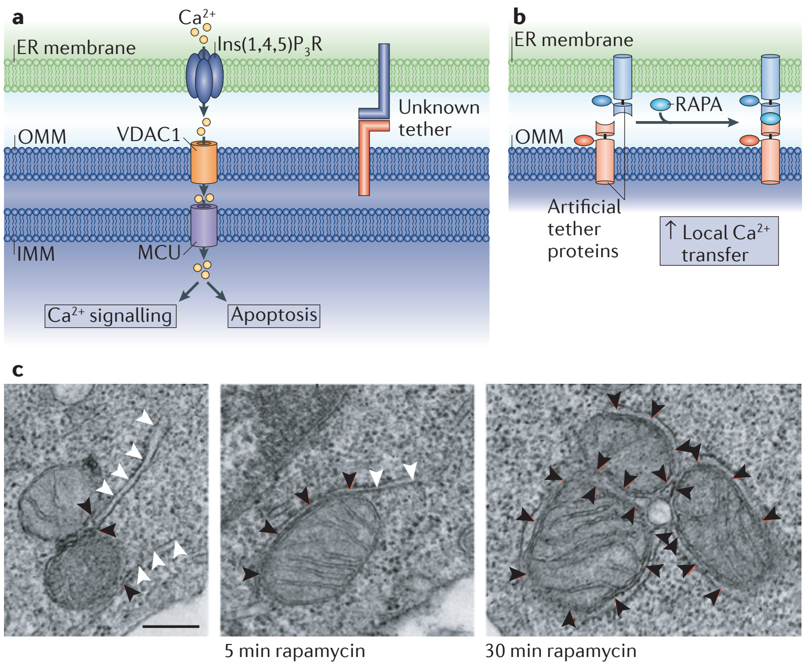

Figure 4 | **Multiple roles of Ca^{2+} transfer between the ER and mitochondrial membranes. a** | Ca^{2+} transfer is proposed to occur from the endoplasmic reticulum (ER) lumen into the mitochondria at contact sites. This requires the inositol 1,4,5-trisphosphate receptor (Ins(1,4,5)P_3R) on the ER membrane, and Ca^{2+} uptake is thought to be mediated by voltage-dependent anion selective channel protein 1 (VDAC1) on the outer mitochondrial membrane (OMM). More recently, the mitochondrial calcium uniporter (MCU) has been identified as the regulator of Ca^{2+} uptake at the inner mitochondrial membrane (IMM), and this is likely to require Ca^{2+} concentrations found near ER–mitochondria contacts. **b** | A rapamycin-inducible tether has demonstrated the importance of tethering between the ER and mitochondria for Ca^{2+} transfer. Half of the tether is localized to the OMM by fusing a mitochondrial localization signal (taken from mitochondrial A-kinase anchor protein 1 (AKAP1), residues 34–63) to 12 kDa FK506-binding protein (FKBP12)–mitochondrial red fluorescent protein 1 (mRFP1) (red). The partner protein is targeted to the ER membrane using an ER targeting signal (taken from SAC1, residues 521–587) fused to FKBP12 rapamycin binding domain (FRB)–cyan fluorescent protein (CFP) (blue). Treatment with rapamycin (RAPA) induces dimerization between FKBP12 and FRB, and thus membrane tethering; this increases local Ca^{2+} transfer. **c** | An electron microscope tomograph of RBL-2H3 cells expressing the artificial imaged tether before and after rapamycin-induced dimerization. Black arrowheads indicate ER–mitochondria contact, and white arrowheads indicate ER not in contact with mitochondria. Scale bar represents 250 nm. Images in **b** and **c** are reproduced, with permission, from REF. 63 © (2010) Elsevier.

increased Ca^{2+} transfer. Moreover, the spacing distance of the tethered bridge was relevant[63]: if the two membranes were tethered too closely together and could not accommodate the size of Ins(1,4,5)P_3R between them, Ca^{2+} transfer was no longer observed[63]. These data further demonstrate that both contact and its organization define the ability of Ca^{2+} to be transferred between these two organelles (FIG. 4b).

There are three main functions for Ca^{2+} release from the ER to the mitochondria. The first is to provide a high local concentration of Ca^{2+} for mitochondrial membrane proteins that require Ca^{2+} binding for their functions but do not have a low enough K_d to bind Ca^{2+} at cytoplasmic concentrations. Because the lumen of the ER stores a high concentration of free Ca^{2+} (100–500 μM) relative to the cytosol (~100 nM)[64], close apposition to this ER Ca^{2+} store can be used to activate Ca^{2+}-dependent processes at contact sites. For example, regulated Ca^{2+} influx through the IMM into the matrix, which activates the tricarboxylic acid (TCA) cycle in order to generate energy, is likely to require concentrations of Ca^{2+} that could only be generated at ER contact sites. Although free Ca^{2+} moves easily through the OMM, it does not pass easily through the IMM and must go through a highly selective, low-affinity Ca^{2+} channel in the IMM. The molecular identity of the IMM Ca^{2+} channel was only recently discovered and was named the mitochondrial Ca^{2+} uniporter (MCU)[65–68]. It seems likely that MCU Ca^{2+} transport into the matrix would require free Ca^{2+} concentrations that could only be encountered near ER contact sites[66].

Second, mitochondrial division is stimulated by changes in Ca^{2+} concentrations in a DRP1-dependent manner[4]. Interestingly, some of the factors that are found at ER–mitochondria contact sites and are required for proper mitochondrial morphology are regulated by Ca^{2+} binding. The most notable of these is MIRO and its yeast homologue, Gem1, which are important for normal mitochondrial dynamics[15,31,32]. The mechanism for how MIRO or Gem1 affects mitochondrial fragmentation or division is not clear but may require local Ca^{2+} influx at contacts. Gem1 requires its first EF-hand Ca^{2+} binding domain to colocalize with ERMES punctae[12] (FIG. 2c), and this correlation suggests that it could be binding Ca^{2+} at ER–mitochondria contact sites. However, Ca^{2+} efflux from the ER has not yet been demonstrated in yeast.

The third function that has been described for localized concentrations of free Ca^{2+} at the ER–mitochondria interface is the activation of apoptosis[69]. Local Ca^{2+} flux can stimulate apoptosis by opening the mitochondrial permeability transition pore (MPTP), which leads to cytochrome *c* release, propagation of the caspase cascade and, ultimately, apoptosis. Ins(1,4,5)P_3R is the channel that is most likely to be responsible for release of ER Ca^{2+} stores to the opposing mitochondrial membrane during apoptosis. Indeed, depletion of Ins(1,4,5)P_3R from several cell lines confers resistance to apoptotic stimuli[70,71]. Aside from Ins(1,4,5)P_3R, there are other ER-localized factors that are implicated in regulating apoptosis. For example, promyelocytic leukaemia (PML) protein has been localized to the ER membrane both using immunofluorescence analysis and biochemically[72,73]. PML forms a complex with Ins(1,4,5)P_3R and is proposed to regulate Ca^{2+} release at the ER membrane in response to apoptotic stimuli[72]. The removal of PML from mouse embryonic fibroblasts reduces the Ca^{2+} response to oxidative apoptotic stimuli, and this Ca^{2+} response can be rescued by a PML construct that is artificially targeted to the ER, demonstrating that PML mediates its effects at the ER membrane[72]. Interaction between the ER protein BAP31 (B-cell receptor-associated protein 31) and the mitochondrial protein FIS1 is also required for the progression of apoptosis. Depletion of FIS1 confers protection against cell death[74] by preventing BAP31 cleavage, which alters procaspase 8 activation in response to apoptotic signals[75].

© 2012 Macmillan Publishers Limited. All rights reserved

Dramatic DRP1-mediated mitochondrial division occurs during apoptosis, although mitochondria fragmentation still occurs even in the absence of DRP1 (REFS 76–78). This role of DRP1 in apoptosis may be independent of its role in mitochondrial fission because DRP1 is also important for the mitochondrial outer-membrane permeabilization (MOMP) that is required for proper cytochrome *c* release during apoptosis[77,79]. When Ca^{2+} is released from the ER to the mitochondria during apoptosis, BAX and BAK, two proapoptotic members of the BCL-2 family, facilitate MOMP[79,80]. DRP1 enhances MOMP by stimulating BAX oligomerization on the mitochondrial membrane. Interestingly, BAX and BAK in turn promote the stable association of the division dynamin, DRP1, to the mitochondrial membrane during apoptosis[81]. Thus, BAX, BAK and DRP1 co-regulate the accumulation of each other on the mitochondria. As DRP1 is recruited to sites of ER–mitochondria contacts during mitochondrial division under non-apoptotic conditions[4], it is possible that DRP1, BAX and BAK could also colocalize together at ER contact sites to coordinate the activation of MOMP. So, although DRP1-dependent mitochondrial division may not be required during apoptosis, ER tubules may be required to recruit another mediator of mitochondrial division for apoptosis.

Conclusions

It is clear that the interface between the ER and mitochondrial membranes has diverse roles. The multiple functions that occur at these contact sites might all be synergized. For example, lipid biosynthesis may be regulated at contact sites that are tethered by ERMES; and ERMES punctae colocalize with Gem1. Gem1 and its mammalian homologue MIRO are Ca^{2+}-binding proteins, and MIRO also marks contact sites between the ER and mitochondria. MIRO is linked to mitochondrial dynamics on microtubules, and these dynamics are regulated by Ca^{2+} flux. Moreover, mitochondria fragment or divide when MIRO or Gem1 is depleted or Ca^{2+} levels are altered, and mitochondrial division occurs at ER contact sites. Thus, the factors at ER–mitochondria contacts that control lipid biosynthesis, Ca^{2+} signalling and mitochondrial dynamics and division are intimately entwined. These connections suggest that all of these processes could be co-regulated at ER contacts. What needs to be done now is to determine whether there is one tether or many that mediate contact site formation. If there are several, it will be important to address the functions of each and the mechanisms of their formation. It is currently not known how many discrete ER contact sites are present on any given single mitochondria or the percentage of the surface area of a mitochondria that is covered by the ER. However, assessing how depletion of candidate tethers affects the number and structure of contact sites using EM tomography could be one way to begin to address these questions. Finally, it will be important to address the significance of ER–mitochondria contacts for disease. It is compelling that defects in both ER structural proteins and factors involved in mitochondrial division and dynamics are all associated with neurodegenerative diseases[82–84]. This raises the question of whether these diseases might result from changes in ER–mitochondria contact that are affecting one or multiple functions that occur at these sites.

1. Elbaz, Y. & Schuldiner, M. Staying in touch: the molecular era of organelle contact sites. *Trends Biochem. Sci.* **36**, 616–623 (2011).
2. Friedman, J. R. & Voeltz, G. K. The ER in 3D: a multifunctional dynamic membrane network. *Trends Cell Biol.* **21**, 709–717 (2011).
3. Csordás, G. *et al.* Structural and functional features and significance of the physical linkage between ER and mitochondria. *J. Cell Biol.* **174**, 915–921 (2006).
4. Friedman, J. R. *et al.* ER tubules mark sites of mitochondrial division. *Science* **334**, 358–362 (2011).
Demonstrates that mitochondrial division occurs at positions where ER tubules contact mitochondria, and that contact and constriction occurs prior to recruitment of the division machinery.
5. Friedman, J. R., Webster, B. M., Mastronarde, D. N., Verhey, K. J. & Voeltz, G. K. ER sliding dynamics and ER-mitochondrial contacts occur on acetylated microtubules. *J. Cell Biol.* **190**, 363–375 (2010).
6. Vance, J. Phospholipid synthesis in a membrane fraction associated with mitochondria. *J. Biol. Chem.* **265**, 7248–7256 (1990).
7. Stone, S. J. & Vance, J. E. Phosphatidylserine synthase-1 and -2 are localized to mitochondria-associated membranes. *J. Biol. Chem.* **275**, 34534–34540 (2000).
8. Voelker, D. R. Interorganelle transport of aminoglycerophospholipids. *Biochim. Biophys. Acta* **1486**, 97–107 (2000).
9. van Meer, G., Voelker, D. R. & Feigenson, G. W. Membrane lipids: where they are and how they behave. *Nature Rev. Mol. Cell. Biol.* **9**, 112–124 (2008).
10. Osman, C., Voelker, D. R. & Langer, T. Making heads or tails of phospholipids in mitochondria. *J. Cell Biol.* **192**, 7–16 (2011).
11. Kornmann, B. *et al.* An ER-mitochondria tethering complex revealed by a synthetic biology screen. *Science* **325**, 477–481 (2009).
A screen for mutants that could be rescued by an artificial tether between the ER and mitochondria, which led to the identification of a tethering complex consisting of Mmm1, Mdm10, Mdm12 and Mdm34 proteins, residents of both ER and mitochondrial membranes.
12. Kornmann, B., Osman, C. & Walter, P. The conserved GTPase Gem1 regulates endoplasmic reticulum-mitochondria connections. *Proc. Natl Acad. Sci. USA* **108**, 2–7 (2011).
13. Toulmay, A. & Prinz, W. A. A conserved membrane-binding domain targets proteins to organelle contact sites. *J. Cell Sci.* **125**, 49–58 (2012).
Demonstrated that the SMP domains of yeast proteins are required for localization to membrane contact sites. Three of the four ERMES components contain SMP domains.
14. Kopec, K. O., Alva, V. & Lupas, A. N. Homology of SMP domains to the TULIP superfamily of lipid-binding proteins provides a structural basis for lipid exchange between ER and mitochondria. *Bioinformatics* **26**, 1927–1931 (2010).
15. Nguyen, T. T. *et al.* Gem1 and ERMES do not directly affect phosphatidylserine transport from ER to mitochondria or mitochondrial inheritance. *Traffic* **13**, 880–890 (2012).
16. Ingerman, E. *et al.* Dnm1 forms spirals that are structurally tailored to fit mitochondria. *J. Cell Biol.* **170**, 1021–1027 (2005).
17. Bleazard, W. *et al.* The dynamin-related GTPase Dnm1 regulates mitochondrial fission in yeast. *Nature Cell Biol.* **1**, 298–304 (1999).
18. Labrousse, A. M., Zappaterra, M. D., Rube, D. A. & van der Bliek, A. M. *C. elegans* dynamin-related protein DRP-1 controls severing of the mitochondrial outer membrane. *Mol. Cell* **4**, 815–826 (1999).
19. Smirnova, E., Griparic, L., Shurland, D. L. & van der Bliek, A. M. Dynamin-related protein Drp1 is required for mitochondrial division in mammalian cells. *Mol. Biol. Cell* **12**, 2245–2256 (2001).
20. Tieu, Q., Okreglak, V., Naylor, K. & Nunnari, J. The WD repeat protein, Mdv1p, functions as a molecular adaptor by interacting with Dnm1p and Fis1p during mitochondrial fission. *J. Cell Biol.* **158**, 445–452 (2002).
21. Mozdy, A. D., McCaffery, J. M. & Shaw, J. M. Dnm1p GTPase-mediated mitochondrial fission is a multi-step process requiring the novel integral membrane component Fis1p. *J. Cell Biol.* **151**, 367–380 (2000).
22. Tieu, Q. & Nunnari, J. Mdv1p is a WD repeat protein that interacts with the dynamin-related GTPase, Dnm1p, to trigger mitochondrial division. *J. Cell Biol.* **151**, 353–366 (2000).
23. Otera, H. *et al.* Mff is an essential factor for mitochondrial recruitment of Drp1 during mitochondrial fission in mammalian cells. *J. Cell Biol.* **191**, 1141–1158 (2010).
Showed that MFF is required for mitochondrial recruitment of DRP1 and therefore fission. MFF function is independent of FIS1, which the authors found was dispensable for mitochondrial fission.
24. Gandre-babbe, S. & van der Bliek, A. M. The novel tail-anchored membrane protein Mff controls mitochondrial and peroxisomal fission in mammalian cells. *Mol. Biol. Cell* **19**, 2402–2412 (2008).
25. Legesse-miller, A., Massol, R. H. & Kirchhausen, T. Constriction and Dnm1p recruitment are distinct processes in mitochondrial fission. *Mol. Biol. Cell* **14**, 1953–1963 (2003).
26. Chen, H. *et al.* Mitofusins Mfn1 and Mfn2 coordinately regulate mitochondrial fusion and are essential for embryonic development. *J. Cell Biol.* **160**, 189–200 (2003).

© 2012 Macmillan Publishers Limited. All rights reserved

27. de Brito, O. M. & Scorrano, L. Mitofusin 2 tethers endoplasmic reticulum to mitochondria. *Nature* **456**, 605–610 (2008).
Showed that MFN2 is enriched at ER–mitochondria contact sites and that ablation or silencing in mouse embryonic fibroblasts and HeLa cells disrupts contact and ultimately mitochondrial Ca^{2+} uptake.
28. Pilling, A. D., Horiuchi, D., Lively, C. M. & Saxton, W. M. Kinesin-1 and Dynein are the primary motors for fast transport of mitochondria in *Drosophila* motor axons. *Mol. Biol. Cell* **17**, 2057–2068 (2006).
29. Woźniak, M. J. *et al.* Role of kinesin-1 and cytoplasmic dynein in endoplasmic reticulum movement in VERO cells. *J. Cell Sci.* **122**, 1979–1989 (2009).
30. Glater, E. E., Megeath, L. J., Stowers, R. S. & Schwarz, T. L. Axonal transport of mitochondria requires milton to recruit kinesin heavy chain and is light chain independent. *J. Cell Biol.* **173**, 545–557 (2006).
31. Saotome, M. *et al.* Bidirectional Ca^{2+}-dependent control of mitochondrial dynamics by the Miro GTPase. *Proc. Natl Acad. Sci. USA* **105**, 20728–20733 (2008).
Demonstrated that mitochondria movements are enhanced by MIRO overexpression and that MIRO is important for Ca^{2+}-induced arrest of mitochondrial motility.
32. Frederick, R. L., Mccaffery, J. M., Cunningham, K. W., Okamoto, K. & Shaw, J. M. Yeast Miro GTPase, Gem1p, regulates mitochondrial morphology via a novel pathway. *J. Cell Biol.* **167**, 87–98 (2004).
33. Stroud, D. A. *et al.* Composition and topology of the endoplasmic reticulum-mitochondria encounter structure. *J. Mol. Biol.* **413**, 743–750 (2011).
34. Fehrenbacher, K., Davis, D. & Wu, M. Endoplasmic reticulum dynamics, inheritance, and cytoskeletal interactions in budding yeast. *Mol. Biol. Cell* **13**, 854–865 (2002).
35. Du, Y., Pypaert, M., Novick, P. & Ferro-Novick, S. Aux1p/Swa2p is required for cortical endoplasmic reticulum inheritance in *Saccharomyces cerevisiae*. *Mol. Biol. Cell* **12**, 2614–2628 (2001).
36. Du, Y., Ferro-Novick, S. & Novick, P. Dynamics and inheritance of the endoplasmic reticulum. *J. Cell Sci.* **117**, 2871–2878 (2004).
37. West, M., Zurek, N., Hoenger, A. & Voeltz, G. K. A. 3D analysis of yeast ER structure reveals how ER domains are organized by membrane curvature. *J. Cell Biol.* **193**, 333–346 (2011).
38. Ueda, H. *et al.* Myosin-dependent endoplasmic reticulum motility and F-actin organization in plant cells. *Proc. Natl Acad. Sci. USA* **107**, 6894–6899 (2010).
39. Estrada, P. *et al.* Myo4p and She3p are required for cortical ER inheritance in *Saccharomyces cerevisiae*. *J. Cell Biol.* **163**, 1255–1266 (2003).
40. Simon, V. R., Swayne, T. C. & Pon, L. A. Actin-dependent mitochondrial motility in mitotic yeast and cell-free systems: identification of a motor activity on the mitochondrial surface. *J. Cell Biol.* **130**, 345–354 (1995).
41. Itoh, T., Watabe, A., Toh-e, A. & Matsui, Y. Complex formation with Ypt11p, a rab-type small GTPase, is essential to facilitate the function of Myo2p, a class V myosin, in mitochondrial distribution in *Saccharomyces cerevisiae*. *Mol. Cell. Biol.* **22**, 7744–7757 (2002).
42. Frederick, R., Okamoto, K. & Shaw, J. M. Multiple pathways influence mitochondrial inheritance in budding yeast. *Genetics* **837**, 825–837 (2008).
43. Suelmann, R. & Fischer, R. Mitochondrial movement and morphology depend on an intact actin cytoskeleton in *Aspergillus nidulans*. *Cell. Motil. Cytoskeleton* **45**, 42–50 (2000).
44. Altmann, K., Frank, M., Neumann, D., Jakobs, S. & Westermann, B. The class V myosin motor protein, Myo2, plays a major role in mitochondrial motility in *Saccharomyces cerevisiae*. *J. Cell Biol.* **181**, 119–130 (2008).
45. Fortsch, J., Hummel, E., Krist, M. & Westermann, B. The myosin-related motor protein Myo2 is an essential mediator of bud-directed mitochondrial movement in yeast. *J. Cell Biol.* **194**, 473–488 (2011).
46. Valiathan, R. R. & Weisman, L. S. Pushing for answers: is myosin V directly involved in moving mitochondria? *J. Cell Biol.* **181**, 15–18 (2008).
47. Itoh, T., Toh-e, A. & Matsui, Y. Mmr1p is a mitochondrial factor for Myo2p-dependent inheritance of mitochondria. *EMBO J.* **23**, 2520–2530 (2004).
48. Swayne, T. C. *et al.* Role for cER and Mmr1p in anchorage of mitochondria at sites of polarized surface growth in budding yeast. *Curr. Biol.* **21**, 1994–1999 (2011).
Demonstrated that Mmr1, a member of the DSL1 family of tethering proteins, localizes to the ER–mitochondria junction of the bud tip and that its deletion impairs bud tip anchorage of mitochondria, although ER distribution is unaffected.
49. Sogo, L. F. & Yaffe, M. P. Regulation of mitochondrial morphology and inheritance by Mdm10p, a protein of the mitochondrial outer membrane. *J. Cell Biol.* **126**, 1361–1373 (1994).
50. McConnell, S. J., Stewart, L. C., Talin, A. & Yaffe, M. P. Temperature-sensitive yeast mutants defective in mitochondrial inheritance. *J. Cell Biol.* **111**, 967–976 (1990).
51. Meisinger, C. *et al.* The morphology proteins Mdm12/Mmm1 function in the major β-barrel assembly pathway of mitochondria. *EMBO J.* **26**, 2229–2239 (2007).
52. Puhka, M., Vihinen, H., Joensuu, M. & Jokitalo, E. Endoplasmic reticulum remains continuous and undergoes sheet-to-tubule transformation during cell division in mammalian cells. *J. Cell Biol.* **179**, 895–909 (2007).
53. Lu, L., Ladinsky, M. S. & Kirchhausen, T. Cisternal organization of the endoplasmic reticulum during mitosis. *Mol. Biol. Cell* **20**, 3471–3480 (2009).
54. Puhka, M., Joensuu, M., Vihinen, H., Belevich, I. & Jokitalo, E. Progressive sheet-to-tubule transformation is a general mechanism for endoplasmic reticulum partitioning in dividing mammalian cells. *Mol. Biol. Cell* **23**, 2424–2432 (2012).
55. Anderson, D. J. & Hetzer, M. W. Nuclear envelope formation by chromatin-mediated reorganization of the endoplasmic reticulum. *Nature Cell Biol.* **9**, 1160–1166 (2007).
56. Taguchi, N., Ishihara, N., Jofuku, A., Oka, T. & Mihara, K. Mitotic phosphorylation of dynamin-related GTPase Drp1 participates in mitochondrial fission. *J. Biol. Chem.* **282**, 11521–11529 (2007).
57. Kashatus, D. F. *et al.* RALA and RALBP1 regulate mitochondrial fission at mitosis. *Nature Cell Biol.* **13**, 1108–1115 (2011).
58. Rizzuto, R., Brini, M., Murgia, M. & Pozzan, T. Microdomains with high Ca^{2+} close to IP3-sensitive channels that are sensed by neighboring mitochondria. *Science* **262**, 744–747 (1993).
59. Rizzuto, R. Close contacts with the endoplasmic reticulum as determinants of mitochondrial Ca^{2+} responses. *Science* **280**, 1763–1766 (1998).
Used a Ca^{2+}-sensitive photoprotein to image the ER–mitochondria junction and demonstrated that increased Ca^{2+} flow from the ER Ins(1,4,5)P_3R results in higher levels of Ca^{2+} on the mitochondrial surface and increased Ca^{2+} uptake by mitochondria.
60. Szabadkai, G. *et al.* Chaperone-mediated coupling of endoplasmic reticulum and mitochondrial Ca^{2+} channels. *J. Cell Biol.* **175**, 901–911 (2006).
61. Hayashi, T., Rizzuto, R., Hajnoczky, G. & Su, T.-P. MAM: more than just a housekeeper. *Trends Cell Biol.* **19**, 81–88 (2009).
62. Giacomello, M. *et al.* Ca^{2+} hot spots on the mitochondrial surface are generated by Ca^{2+} mobilization from stores, but not by activation of store-operated Ca^{2+} channels. *Mol. Cell* **38**, 280–290 (2010).
63. Csordás, G. *et al.* Imaging interorganelle contacts and local calcium dynamics at the ER-mitochondrial interface. *Mol. Cell* **39**, 121–132 (2010).
Demonstrated that variable length artificial tethers between the ER and mitochondria can alter the efficiency of Ca^{2+} transfer, suggesting that there is an optimal gap distance for proper Ca^{2+} transfer.
64. Berridge, M. The endoplasmic reticulum: a multifunctional signaling organelle. *Cell Calcium* **32**, 235–249 (2002).
65. De Stefani, D., Raffaello, A., Teardo, E., Szabò, I. & Rizzuto, R. A forty-kilodalton protein of the inner membrane is the mitochondrial calcium uniporter. *Nature* **476**, 336–340 (2011).
66. Rizzuto, R., De Stefani, D., Raffaello, A. & Mammucari, C. Mitochondria as sensors and regulators of calcium signalling. *Nature Rev. Mol. Cell Biol.* **13**, 566–578 (2012).
67. Baughman, J. M. *et al.* Integrative genomics identifies MCU as an essential component of the mitochondrial calcium uniporter. *Nature* **476**, 341–345 (2011).
68. Kirichok, Y., Krapivinsky, G. & Clapham, D. E. The mitochondrial calcium uniporter is a highly selective ion channel. *Nature* **427**, 360–364 (2004).
69. Scorrano, L. *et al.* BAX and BAK regulation of endoplasmic reticulum Ca^{2+}: a control point for apoptosis. *Science* **300**, 135–139 (2003).
70. Jayaraman, T. & Marks, A. R. T cells deficient in inositol 1,4,5-trisphosphate receptor are resistant to apoptosis. *Mol. Cell. Biol.* **17**, 3005–3012 (1997).
71. Khan, A. A. *et al.* Lymphocyte apoptosis: mediation by increased type 3 inositol 1,4,5-trisphosphate receptor. *Science* **273**, 503–507 (1996).
72. Giorgi, C. *et al.* PML regulates apoptosis at endoplasmic reticulum by modulating calcium release. *Science* **330**, 1247–1251 (2010).
73. Pinton, P., Giorgi, C. & Pandolfi, P. P. The role of PML in the control of apoptotic cell fate: a new key player at ER-mitochondria sites. *Cell Death Differ.* **18**, 1450–1456 (2011).
74. Lee, Y., Jeong, S., Karbowski, M., Smith, C. L. & Youle, R. J. Roles of the mammalian mitochondrial fission and fusion mediators Fis1, Drp1, and Opa1 in apoptosis. *Mol. Biol. Cell* **15**, 5001–5011 (2004).
75. Iwasawa, R., Mahul-Mellier, A.-L., Datler, C., Pazarentzos, E. & Grimm, S. Fis1 and Bap31 bridge the mitochondria-ER interface to establish a platform for apoptosis induction. *EMBO J.* **30**, 556–568 (2011).
Demonstrates that FIS1 and BAP31 interact to form a platform for procaspase-8 recruitment and function. This triggers Ca^{2+} release from the ER to the mitochondria, thereby activating apoptosis.
76. Frank, S. *et al.* The role of dynamin-related protein 1, a mediator of mitochondrial fission, in apoptosis. *Dev. Cell* **1**, 515–525 (2001).
77. Cassidy-Stone, A. *et al.* Chemical inhibition of the mitochondrial division dynamin reveals its role in Bax/Bak-dependent mitochondrial outer membrane permeabilization. *Dev. Cell* **14**, 193–204 (2008).
78. Ishihara, N. *et al.* Mitochondrial fission factor Drp1 is essential for embryonic development and synapse formation in mice. *Nature Cell Biol.* **11**, 958–966 (2009).
79. Montessuit, S. *et al.* Membrane remodeling induced by the dynamin-related protein Drp1 stimulates Bax oligomerization. *Cell* **142**, 889–901 (2010).
80. Lucken-Ardjomande, S. & Martinou, J.-C. Regulation of Bcl-2 proteins and of the permeability of the outer mitochondrial membrane. *C. R. Biol.* **328**, 616–631 (2005).
81. Wasiak, S., Zunino, R. & McBride, H. M. Bax/Bak promote sumoylation of DRP1 and its stable association with mitochondria during apoptotic cell death. *J. Cell Biol.* **177**, 439–450 (2007).
82. Nunnari, J. & Suomalainen, A. Mitochondria: in sickness and in health. *Cell* **148**, 1145–1159 (2012).
83. Blackstone, C., O'Kane, C. J. & Reid, E. Hereditary spastic paraplegias: membrane traffic and the motor pathway. *Nature Rev. Neurosci.* **12**, 31–42 (2011).
84. Schon, E. A. & Area-Gomez, E. Is Alzheimer's disease a disorder of mitochondria-associated membranes? *J. Alzheimers. Dis.* **20**, S281–292 (2010).

Acknowledgements
We thank J. Shaw and J. Friedman for helpful comments on the manuscript. This work was supported by a grant from the US National Institutes of Health (NIH), RO1GM083977, to G.K.V. and by an NIH predoctoral training grant, GM07135, to A.A.R.

Competing interests statement
The authors declare no competing financial interests.

FURTHER INFORMATION
Authors' homepage:
http://mcdb.colorado.edu/labs1/voeltzlab/

ALL LINKS ARE ACTIVE IN THE ONLINE PDF

© 2012 Macmillan Publishers Limited. All rights reserved
