# Supplementary material for: Resveratrol reduces the apoptosis induced by cigarette smoke extract by upregulating MFN2
Source: PLoS One. 2017 Apr 13;12(4):e0175009. doi: 10.1371/journal.pone.0175009 (PMC5391199; doi:10.1371/journal.pone.0175009)
Supplement: S3 Text — (PDF) [file pone.0175009.s006.pdf]

in a single cell, noise can substantially restrict the amount of information transduced about input intensity, particularly within individual signaling pathways. The bush and tree network models, which provide a unified theoretical framework for analyzing branched motifs widespread in natural and synthetic signaling networks, further demonstrated that signaling networks can be more effective in information transfer, although bottlenecks can also severely limit the information gained. Receptor-level bottlenecks restrict the TNF and also PDGF signaling networks (fig. S11) and may be prevalent in other signaling systems.

We explored several strategies that a cell might use to overcome restrictions due to noise. We found that negative feedback can suppress bottleneck noise, which can be offset by concomitantly reduced dynamic range of the response. Time integration can increase the information transferred, to the extent that the response undergoes substantial dynamic fluctuations in a single cell over the physiologically relevant time course. The advantage of collective cell responses can also be substantial, but limited by the number of cells exposed to the same signal or by the information present in the initiating signal itself.

Responses incorporating the signaling history of the cell might also increase the information (*40*, *41*). For instance, responses relative to the basal state (fold-change response) might be less susceptible to noise arising from diverse initial states (*23*), although this does not necessarily translate into large amounts of transferred information (table S1). Similarly, for the reporter gene system described here (fig. S12), ~0.5 bits of additional information can be obtained if a cell can determine expression levels at both early and late time points. However, noise in the biochemical networks that a cell uses to record earlier output levels and to later compute the final response may nullify the information gain potentially provided by this strategy. Overall, we anticipate that the information theory paradigm can extend to the analysis of noise-mitigation strategies and information-transfer mechanisms beyond those explored here, in order to determine what specific signaling systems can do reliably despite noise.

References and Notes

1. J. G. Albeck, J. M. Burke, S. L. Spencer, D. A. Lauffenburger, P. K. Sorger, *PLoS Biol.* **6**, e299 (2008).
2. N. Rosenfeld, J. W. Young, U. Alon, P. S. Swain, M. B. Elowitz, *Science* **307**, 1962 (2005).
3. T. J. Perkins, P. S. Swain, *Mol. Syst. Biol.* **5**, 326 (2009).
4. W. J. Blake, M. Kaern, C. R. Cantor, J. J. Collins, *Nature* **422**, 633 (2003).
5. M. B. Elowitz, A. J. Levine, E. D. Siggia, P. S. Swain, *Science* **297**, 1183 (2002).
6. J. Paulsson, *Nature* **427**, 415 (2004).
7. J. M. Pedraza, A. van Oudenaarden, *Science* **307**, 1965 (2005).
8. J. M. Raser, E. K. O'Shea, *Science* **304**, 1811 (2004).
9. T. M. Cover, J. A. Thomas, *Elements of Information Theory* (Wiley, New York, 1991).
10. R. R. de Ruyter van Steveninck, G. D. Lewen, S. P. Strong, R. Koberle, W. Bialek, *Science* **275**, 1805 (1997).
11. D. Fuller *et al.*, *Proc. Natl. Acad. Sci. U.S.A.* **107**, 9656 (2010).
12. E. Ziv, I. Nemenman, C. H. Wiggins, *PLoS ONE* **2**, e1077 (2007).
13. G. Tkacik, C. G. Callan Jr., W. Bialek, *Proc. Natl. Acad. Sci. U.S.A.* **105**, 12265 (2008).
14. P. Mehta, S. Goyal, T. Long, B. L. Bassler, N. S. Wingreen, *Mol. Syst. Biol.* **5**, 325 (2009).
15. R. Cheong, A. Hoffmann, A. Levchenko, *Mol. Syst. Biol.* **4**, 192 (2008).
16. R. Cheong, C. J. Wang, A. Levchenko, *Mol. Cell. Proteomics* **8**, 433 (2009).
17. S. L. Werner *et al.*, *Genes Dev.* **22**, 2093 (2008).
18. S. Tay *et al.*, *Nature* **466**, 267 (2010).
19. L. Ashall *et al.*, *Science* **324**, 242 (2009).
20. D. E. Nelson *et al.*, *Science* **306**, 704 (2004).
21. R. Cheong *et al.*, *J. Biol. Chem.* **281**, 2945 (2006).
22. A. Hoffmann, A. Levchenko, M. L. Scott, D. Baltimore, *Science* **298**, 1241 (2002).
23. C. Cohen-Saidon, A. A. Cohen, A. Sigal, Y. Liron, U. Alon, *Mol. Cell* **36**, 885 (2009).
24. X. R. Bao, I. D. Fraser, E. A. Wall, S. R. Quake, M. I. Simon, *Biophys. J.* **99**, 2414 (2010).
25. M. Coppey, A. N. Boettiger, A. M. Berezhkovskii, S. Y. Shvartsman, *Curr. Biol.* **18**, 915 (2008).
26. B. B. Averbeck, P. E. Latham, A. Pouget, *Nat. Rev. Neurosci.* **7**, 358 (2006).
27. J. W. Pillow *et al.*, *Nature* **454**, 995 (2008).
28. E. Schneidman, W. Bialek, M. J. Berry II, *J. Neurosci.* **23**, 11539 (2003).
29. H. Wajant, K. Pfizenmaier, P. Scheurich, *Cell Death Differ.* **10**, 45 (2003).
30. A. Becskei, L. Serrano, *Nature* **405**, 590 (2000).
31. I. Lestas, G. Vinnicombe, J. Paulsson, *Nature* **467**, 174 (2010).
32. R. C. Yu *et al.*, *Nature* **456**, 755 (2008).
33. I. E. Wertz *et al.*, *Nature* **430**, 694 (2004).
34. E. G. Lee *et al.*, *Science* **289**, 2350 (2000).
35. V. Shahrezaei, P. S. Swain, *Proc. Natl. Acad. Sci. U.S.A.* **105**, 17256 (2008).
36. S. Krishna, M. H. Jensen, K. Sneppen, *Proc. Natl. Acad. Sci. U.S.A.* **103**, 10840 (2006).
37. S. Thierfelder, K. Ostermann, A. Göbel, G. Rödel, *Appl. Biochem. Biotechnol.* **163**, 954 (2011).
38. K. Francis, B. O. Palsson, *Proc. Natl. Acad. Sci. U.S.A.* **94**, 12258 (1997).
39. J. Parkin, B. Cohen, *Lancet* **357**, 1777 (2001).
40. I. Nemenman, G. D. Lewen, W. Bialek, R. R. de Ruyter van Steveninck, *PLOS Comput. Biol.* **4**, e1000025 (2008).
41. S. P. Strong, R. Koberle, R. R. de Ruyter van Steveninck, W. Bialek, *Phys. Rev. Lett.* **80**, 197 (1998).

Acknowledgments: We thank A. Hoffmann, M. Simon, S. Shvartsman, C. Cohen-Saidon, and U. Alon for sharing data and materials; A. Ganesan and H. Chang for experimental assistance; and P. Iglesias, Y. Qi, and A. Feinberg for insightful discussions and reviewing drafts of the manuscript. This work was supported by NIH grants GM072024 and RR020839 (R.C., A.R., C.J.W., and A.L.) and CA132629 (I.N.), the Medical Scientist Training Program at the Johns Hopkins University (R.C.), and, in early stages of the work, the Los Alamos National Laboratory Directed Research and Development program (I.N.).

Supporting Online Material

www.sciencemag.org/cgi/content/full/science.1204553/DC1
Materials and Methods
SOM Text
Figs. S1 to S12
Table S1
References (*42–55*)

18 February 2011; accepted 7 September 2011
Published online 15 September 2011;
10.1126/science.1204553

ER Tubules Mark Sites of Mitochondrial Division

Jonathan R. Friedman,[1] Laura L. Lackner,[2] Matthew West,[1] Jared R. DiBenedetto,[1] Jodi Nunnari,[2] Gia K. Voeltz[1]*

Mitochondrial structure and distribution are regulated by division and fusion events. Mitochondrial division is regulated by Dnm1/Drp1, a dynamin-related protein that forms helices around mitochondria to mediate fission. Little is known about what determines sites of mitochondrial fission within the mitochondrial network. The endoplasmic reticulum (ER) and mitochondria exhibit tightly coupled dynamics and have extensive contacts. We tested whether ER plays a role in mitochondrial division. We found that mitochondrial division occurred at positions where ER tubules contacted mitochondria and mediated constriction before Drp1 recruitment. Thus, ER tubules may play an active role in defining the position of mitochondrial division sites.

Regulation of mitochondrial division is critical to normal cellular function; excess division is linked to numerous diseases, including neurodegeneration and diabetes (*1*, *2*). The central player in mitochondrial division is the highly conserved dynamin-related protein (Drp1 in mammals, Dnm1 in yeast), which belongs to a family of large guanosine triphosphatases (GTPases) that self-assemble to regulate membrane structure (*3*). Division dynamins are likely to work by oligomerizing in a GTP-dependent manner into helices that wrap around mitochondria; locally controlled assembly-stimulated GTP hydrolysis is thought to provide the mechanochemical force that completes fission of the outer and inner membranes (*4*). There are additional proteins required for mitochondrial division, such as the outer membrane protein Mff (mitochondrial fission factor), which is present only in mammals (*5*). Although general mechanisms exist for

[1]Department of Molecular, Cellular, and Developmental Biology, University of Colorado, Boulder, CO 80309, USA. [2]Department of Molecular and Cellular Biology, University of California, Davis, CA 95616, USA.

*To whom correspondence should be addressed. E-mail: gia.voeltz@colorado.edu

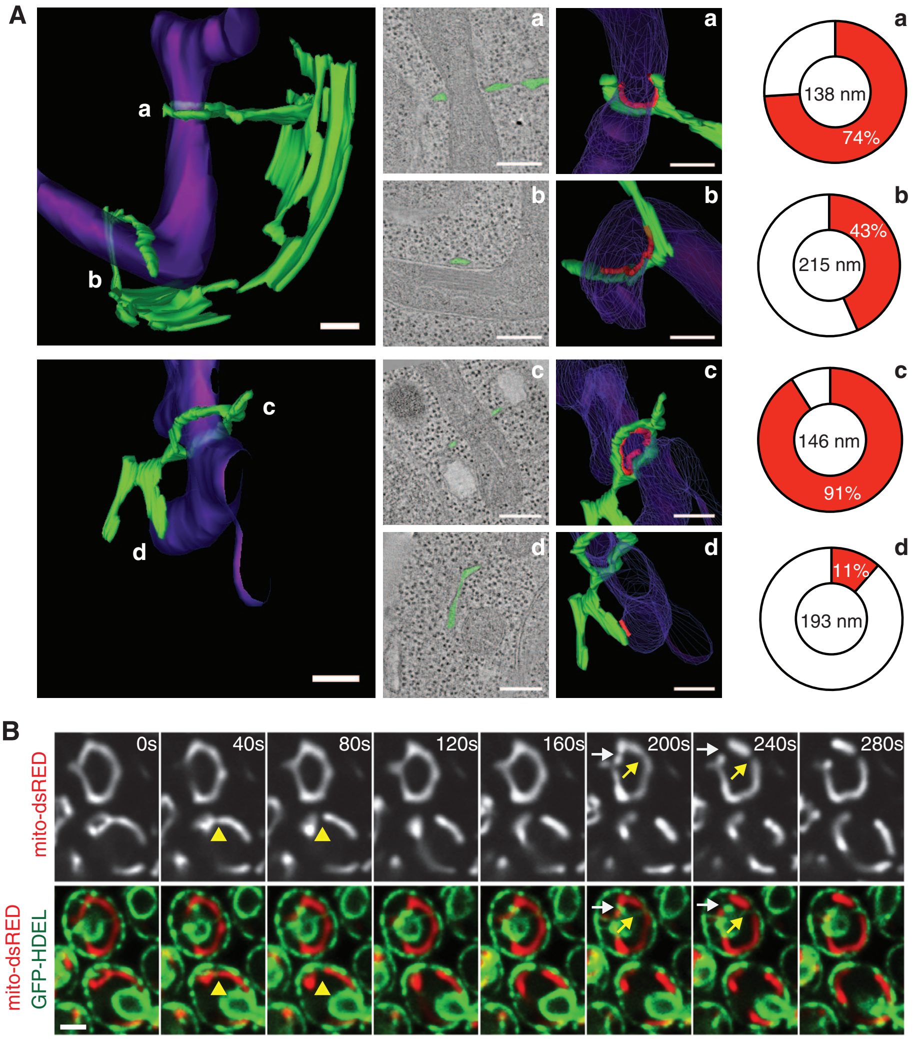

Fig. 1. Mitochondrial constriction and division occurs at ER-mitochondrial contacts in yeast. (**A**) The 3D models (left images) of ER (green) and mitochondria (purple) at contact domains were imaged by EM and tomography of high-pressure frozen yeast cells. Middle images are 2D tomographs of contact sites (second column, ER drawn in green) and the corresponding 3D models of each (third column). Contact, marked in red, is defined as regions where the ER membrane comes within 30 nm of the mitochondrial membrane, and ribosomes are excluded (third column). Right schematics demonstrate the percentage of the mitochondrial circumference that makes contact with the ER membrane [red is contact, white is not (*19*)]. The diameter of each mitochondrion at positions of ER contact is shown. Regions where the mitochondria are constricted (models a and c) have a high percent of ER wrapping. Additional EM tomographs and analysis of constrictions are shown in fig. S1, A and B. (**B**) Time-lapse images of yeast cells expressing mito-dsRed and GFP-HDEL (ER). A single focal plane is shown. Arrows and arrowheads indicate sites of mitochondrial division. A corresponding z-series is shown in fig. S1C. Scale bars indicate, in (A), 200 nm; (B), 2 μm.

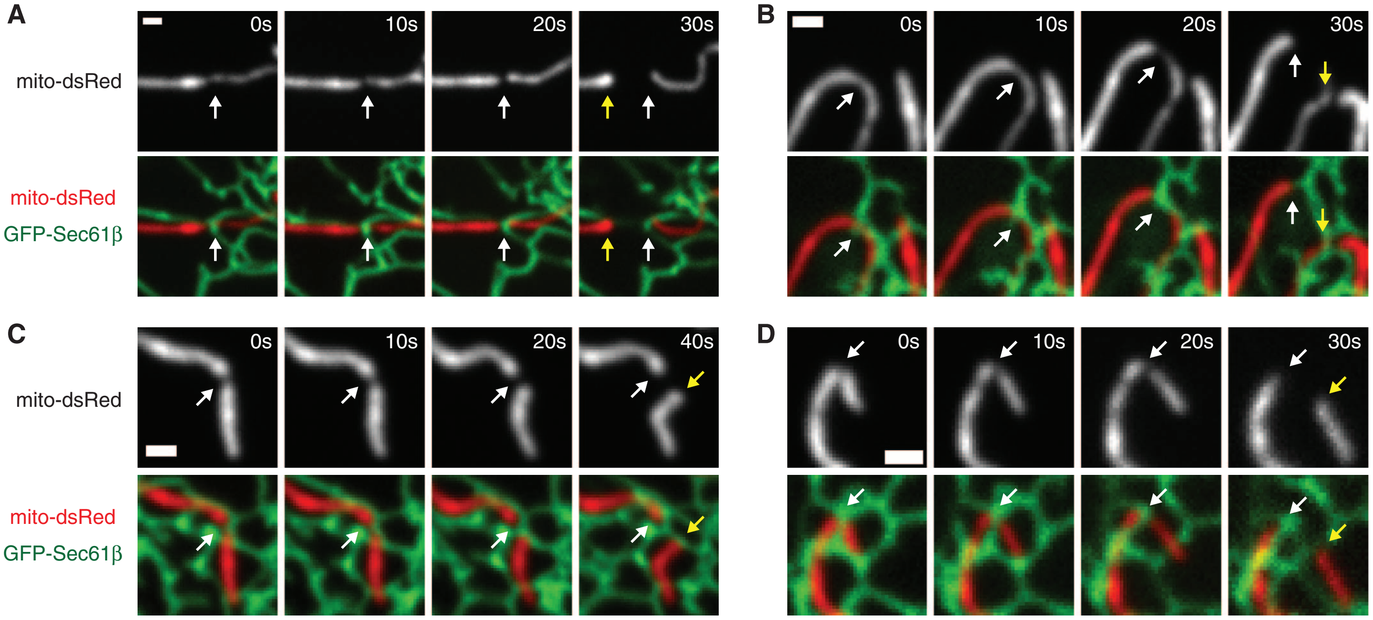

Fig. 2. Mitochondrial division occurs at ER-mitochondrial contact sites in mammalian cells. (**A** to **D**) Four examples of mitochondrial division over time courses shown in Cos-7 cells expressing GFP-Sec61β (ER) and mito-dsRed. The site of mitochondrial division (white arrows) and the position of the newly formed mitochondrial ends (yellow arrows) are shown. Additional examples are included in fig. S2A. Scale bars, 1 μm.

recruiting Dnm1 or Drp1 to mitochondria, it is not known whether there are specific sites on mitochondria that are marked for division (*6*). Furthermore, both Dnm1 and Drp1 oligomerize into helices that are much smaller than the diameter of mitochondria (Dnm1 helices have reported mean diameters of 109 nm in yeast and 129 nm in vitro), suggesting that Dnm1 (Drp1)–independent mitochondrial constriction may be needed to facilitate mitochondrial division (*4*, *6*–*9*).

Contact sites exist between mitochondria and the endoplasmic reticulum (ER) and are important for phospholipid synthesis and calcium signaling [for review, see (*10*)]. Based on recent data, there are likely several types of molecular bridges that mediate these contacts, such as the ERMES complex identified in yeast and the mitochondrial fusion protein mitofusin 2 (Mfn2) in mammalian cells (*11*, *12*). These physical contacts are persistent and maintained under dynamic conditions (*13*), suggesting that the ER-mitochondrial interface is vital for function. We have used electron microscopy (EM) and tomography to analyze the three-dimensional (3D) structure of contacts between the ER and mitochondria in the yeast *Saccharomyces cerevisiae*. We observed the high-resolution (~4 nm) structure and 3D models of four ER-mitochondrial contacts taken from two cells (Fig. 1A). In these examples, the ER was wrapped around mitochondria to varying degrees. In two of the four examples, the ER almost completely circumscribed the mitochondrial outer membrane, and mitochondria were constricted at the point of contact (mitochondrial diameter 138 nm and 146 nm circumscribed versus 215 nm and 193 nm uncircumscribed at ER contact) (Fig. 1A; fig. S1, A and B; and movies S1 and S2). These data suggest that ER tubules associate with and may mediate mitochondrial constriction sites.

We thus examined the role of ER in mitochondrial division by using fluorescence microscopy in live yeast cells transformed with an ER marker (GFP-HDEL) and mito-dsRed to image the behavior of ER and mitochondria simultaneously over time. The vast majority of mitochondrial division events were spatially linked to sites of ER-mitochondrial contact (87%, *n* = 112

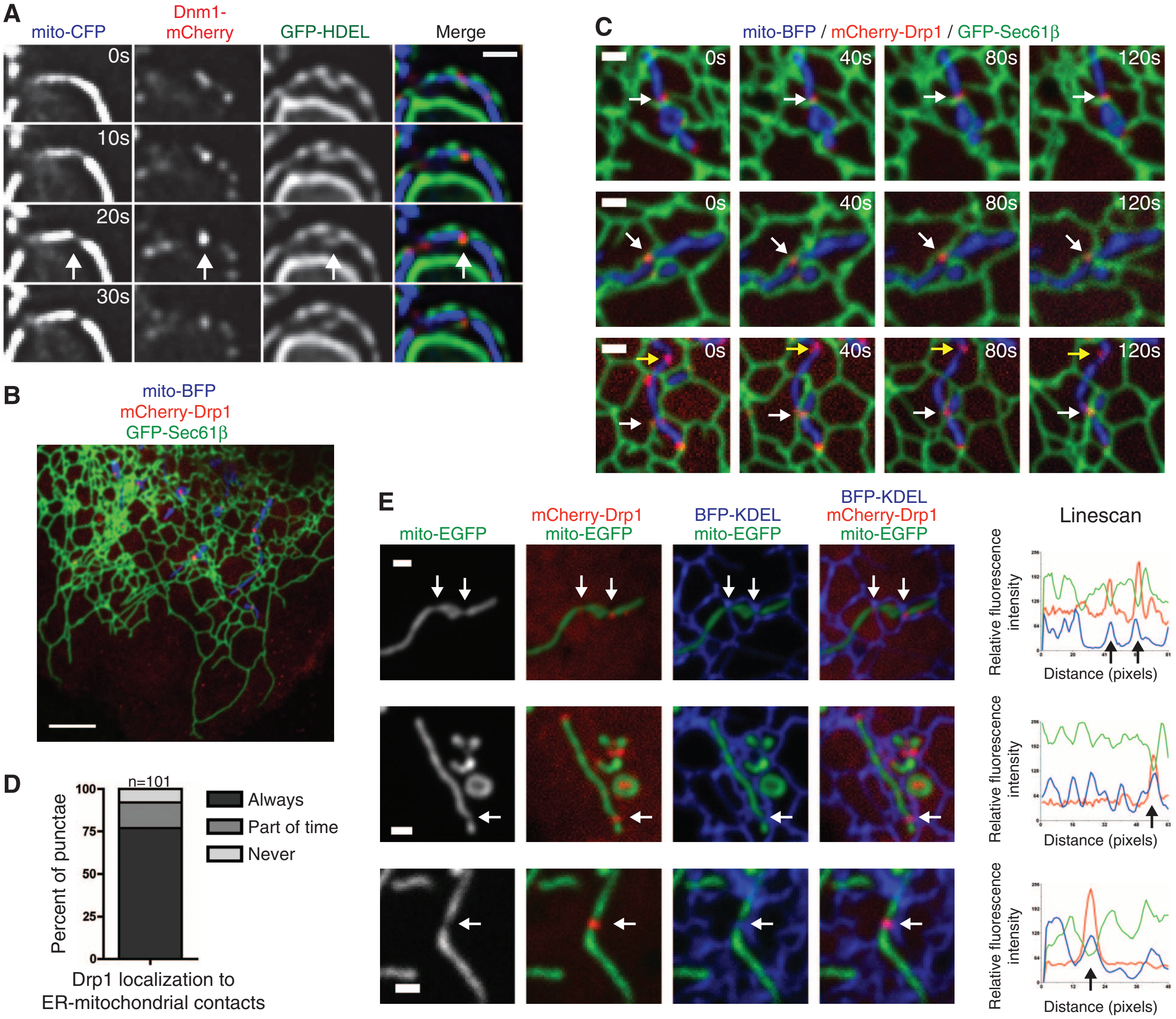

Fig. 3. Dnm1- and Drp1-mediated mitochondrial division occurs at ER contact sites. (**A**) Time-lapse images of wild-type yeast cells expressing mito-CFP, GFP-HDEL (ER), and Dnm1-mCherry. A single focal plane is shown. Arrows indicate the site of mitochondrial division, which is marked by both ER-mitochondria contact and Dnm1. (**B**) Merged image of a live Cos-7 cell expressing GFP-Sec61β (ER), mito-BFP, and mCherry-Drp1. (**C**) Examples of cells as in (B) that show that Drp1 punctae maintain colocalization with positions of ER-mitochondrial contact over time. White arrows indicate Drp1 punctae that maintain contact with both the ER and mitochondria. Yellow arrows indicate a rare example of Drp1 that does not contact the ER. (**D**) The percentage of mitochondrial Drp1 punctae that colocalize with the ER membrane over a 2-min time course. (**E**) Examples of mitochondrial constrictions at ER contact sites marked by Drp1. Left-hand images show Cos-7 cells expressing mito-EGFP, BFP-KDEL (ER), and mCherry-Drp1, merged as indicated. Right graphs are line scans drawn through the mitochondria and show the relative fluorescence intensity of mitochondria (green), ER (blue), and Drp1 (red) along its length. White arrows at constrictions on images correspond to black arrows shown on the line scan. Additional examples are shown in fig. S4. Scale bars for (A), (C), and (E), 1 μm; (B), 5 μm.

from 281 cells) (Fig. 1B). ER tubules crossed over (Fig. 1B, yellow arrows) and wrapped around mitochondria (Fig. 1B, white arrows, and fig. S1C). At ER-mitochondrial contact sites, mitochondrial constriction followed by mitochondrial division was observed (Fig. 1B).

We next tested whether ER plays a similar role in mammalian mitochondrial division by using fluorescence microscopy of live Cos-7 cells transiently transfected with fluorescent markers for ER (GFP-Sec61β) and mitochondria (mito-dsRed). We imaged regions of the cell periphery where contacts between the mitochondria and ER were well resolved and observed that mitochondrial division events predominantly occurred at sites of contact between ER and mitochondria (94%, $n = 32$ from 23 cells) (Fig. 2, fig. S2A, and movies S3 and S4). Furthermore, the majority of events (88%) were sites of ER tubules crossing over the mitochondria, suggesting that the structural context of the interaction is important. The frequency of ER-associated mitochondrial division is much higher than would be predicted on the basis of the area of mitochondria covered by crossing ER tubules as determined by colocalization of mitochondrial and ER markers (fig. S2B).

Thus, in both yeast and mammalian cells, ER tubules are at mitochondrial division sites and may be involved in mitochondrial constriction during this process. Next, we asked whether mitochondrial division occurs in yeast cells that have substantially reduced levels of tubules because of the absence of the membrane shaping proteins Rtns and Yop1 (*14*, *15*). By using both EM and fluorescence microscopic analyses, we observed that, in regions of mutant cells in which ER tubules were dramatically reduced, short ER tubules extended out of the massive ER cisternae and associated with mitochondrial constrictions and division events (fig. S3). Thus, ER tubules are a consistent feature of ER contact at mitochondrial constrictions, even under conditions where most tubules are depleted. Furthermore, Rtns and Yop1 are dispensable for the biogenesis of the ER tubules that associate with mitochondrial division events.

To ask whether ER-associated division events are spatially linked to the mitochondrial division machinery, we determined the relationship of ER-mitochondrial contacts to the division dynamins Dnm1 and Drp1. Dnm1 and Drp1 assemble into punctate structures at steady state, and a subset of these structures are found on mitochondria and at mitochondrial division sites (*6*, *16*, *17*). We imaged live yeast transformed with Dnm1-mCherry, mito–cyan fluorescent protein (CFP), and GFP-HDEL (ER) and observed that a large percentage of Dnm1 punctae were at sites of mitochondrial-ER contact (46%, $n = 225$). These Dnm1 punctae could be observed at sites where ER tubule crossover and mitochondrial division occurred (Fig. 3A). In Cos-7 cells transiently transfected with GFP-Sec61β (ER), mito–blue fluorescent protein (BFP), and mCherry-Drp1, we observed that the majority of Drp1 punctae stably associated with mitochondria and localized to ER-mitochondrial contacts over time (Fig. 3, B to D, and movie S5). Furthermore, a subset of Drp1 at these contacts was associated with a mitochondrial constriction site (78%, excluding punctae localized to mitochondrial tips, $n = 50$). The mitochondrial constrictions marked by Drp1 punctae were always either at ER tubule crossovers (81%) or adjacent to them (19%) (Fig. 3E and fig. S4). Together, the localization of the mitochondrial division dynamins in yeast and mammalian cells to regions of ER-mitochondrial contacts and the observations that these regions are associated with constricted mitochondria and subsequent division indicate a direct role of the ER in the process of mitochondrial division.

Mff is a mammalian-specific mitochondrial outer membrane protein required for mitochondrial localization of Drp1 and division (*5*, *18*). Drp1 and Mff colocalize in punctate structures on mitochondria, and Mff punctae persist in cells where Drp1 expression is reduced by RNAi (*18*). Thus, Mff punctae may mark the future sites of mitochondrial division before Drp1 recruitment (*18*). In Cos-7 cells transiently transfected with

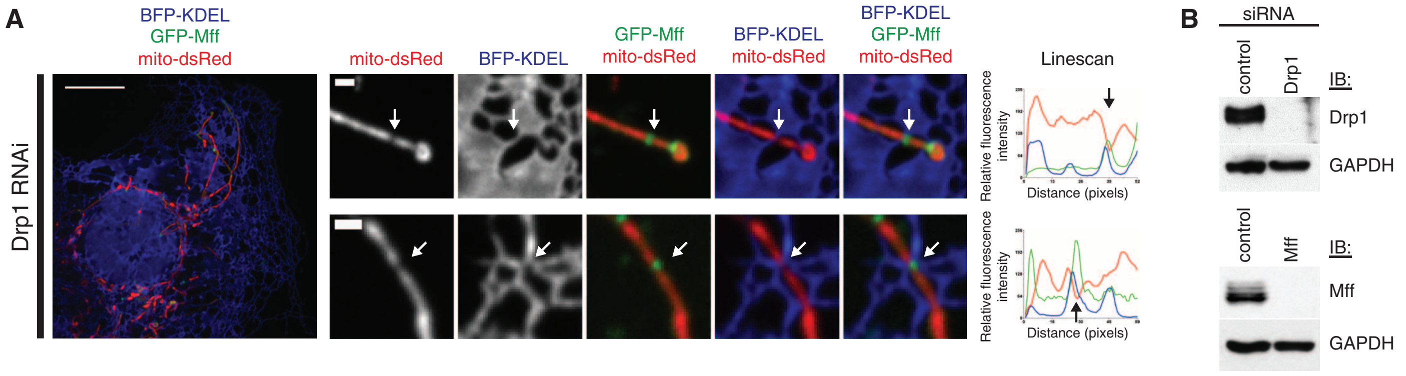

C

Constrictions marked by Mff (Drp1 RNAi)	n = 25
At ER tubule crossing	16 (64%)
Adjacent to ER tubule crossing	4 (16%)
Alongside ER tubule	3 (12%)
Not at ER	2 (8%)

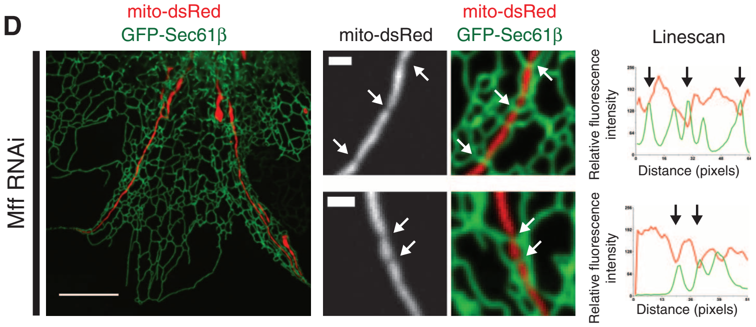

Fig. 4. The ER localizes to mitochondrial constrictions before Drp1 and Mff recruitment. (**A**) Examples of mitochondrial constrictions at ER contacts marked by Mff in Cos-7 cells depleted of Drp1. Left and center images show these cells expressing mito-dsRed, BFP-KDEL (ER), and GFP-Mff, merged as indicated. Right graphs are line scans drawn through the mitochondria and show the relative fluorescence intensity of mitochondria (red), ER (blue), and Mff (green) along their length. White arrow positions at constrictions correspond to black arrows on the line scan. Additional examples are shown in fig. S6. (**B**) Western blots with antibody against Drp1 (top) or Mff (bottom) and GAPDH demonstrate depletion of Drp1 in lysates from cells transfected with siRNA against Drp1 [as in (A)] or Mff [as in (D)] compared with control RNAi cells. (**C**) The number of Mff-localized mitochondrial constrictions in Drp1-depleted cells that colocalize with ER tubules, from 23 cells. (**D**) As in (A), for cells depleted of Mff and expressing GFP-Sec61β (ER; green on line scan) and mito-dsRed (red on line scan). Scale bars for (A) and (D) large left images, 5 μm; (A) and (D) smaller center images, 1 μm.

GFP-Mff, mCherry-Drp1, and mito-BFP, we observed that Mff circumscribed and localized to punctae on mitochondria, the majority of which colocalized with Drp1 (fig. S5, A to C). To test whether Mff punctae localize to ER contacts independently of Drp1, we depleted Drp1 from Cos-7 cells with small interfering RNA (siRNA) and cotransfected these cells with GFP-Mff, mito-dsRed, and BFP-KDEL (ER). Drp1 was substantially depleted in Drp1 RNA interference (RNAi) cells in comparison with the control cells (Fig. 4B). Selective depletion of Drp1 was further supported by the aberrant and elongated mitochondrial morphology in Drp1 RNAi cells (Fig. 4A and fig. S5D). As expected (*18*), in Drp1-depleted cells, Mff punctae localized to mitochondria (Fig. 4A). We asked whether mitochondria were constricted at Mff punctae in the absence of Drp1, and if so, whether these sites localized to ER contacts. Of the 25 constrictions we resolved, 16 were at an ER crossover (64%), and another 4 were adjacent to an ER tubule crossing (16%) (Fig. 4, A and C, and fig. S6). Thus, Mff localizes in a Drp1-independent manner to mitochondrial constrictions at sites of ER contact. We next asked whether the ER localizes to regions of mitochondrial constriction in the absence of Mff. Cos-7 cells were depleted of Mff by siRNA and cotransfected with GFP-Sec61β (ER) and mito-dsRed. As expected, mitochondrial morphology was elongated in these cells (Fig. 4, B and D, and fig. S5E). In cells depleted of Mff, we observed mitochondrial constriction at sites of ER contact, indicating that ER-mitochondrial contacts form and mark positions of mitochondrial constriction independently of both Mff and Drp1 recruitment (Fig. 4D).

Here, we have shown that ER-mitochondrial contacts are a conserved feature of mitochondrial division. We envision two ways that ER contact might directly regulate mitochondrial division: (i) ER proteins intimately participate in division, and/or (ii) ER tubules physically wrap around and constrict mitochondria to a diameter comparable to Dnm1 and Drp1 helices to facilitate their recruitment and assembly to complete fission (fig. S9). The latter is attractive given that the diameter of Dnm1 helices (~110 to 130 nm) is considerably narrower than that of mitochondria and is quite similar to the diameter of constricted mitochondria at ER tubule contacts (138 nm and 146 nm) (*4*, *6*–*9*). Regardless of the exact mechanism, the ER appears to mark the division site and is likely to be an active participant in this process, because it remains in contact with the mitochondria through the entire fission event. Many human diseases are associated with excessive mitochondrial division, raising the intriguing possibility that these diseases could involve an alteration of ER-mitochondrial contacts.

References and Notes

1. D. H. Cho, T. Nakamura, S. A. Lipton, *Cell. Mol. Life Sci.* **67**, 3435 (2010).
2. Y. Yoon, C. A. Galloway, B. S. Jhun, T. Yu, *Antioxid. Redox Signal.* **14**, 439 (2011).
3. L. L. Lackner, J. M. Nunnari, *Biochim. Biophys. Acta* **1792**, 1138 (2009).
4. E. Ingerman *et al.*, *J. Cell Biol.* **170**, 1021 (2005).
5. S. Gandre-Babbe, A. M. van der Bliek, *Mol. Biol. Cell* **19**, 2402 (2008).
6. A. Legesse-Miller, R. H. Massol, T. Kirchhausen, *Mol. Biol. Cell* **14**, 1953 (2003).
7. A. M. Labrousse, M. D. Zappaterra, D. A. Rube, A. M. van der Bliek, *Mol. Cell* **4**, 815 (1999).
8. Y. Yoon, K. R. Pitts, M. A. McNiven, *Mol. Biol. Cell* **12**, 2894 (2001).
9. J. A. Mears *et al.*, *Nat. Struct. Mol. Biol.* **18**, 20 (2011).
10. O. M. de Brito, L. Scorrano, *EMBO J.* **29**, 2715 (2010).
11. O. M. de Brito, L. Scorrano, *Nature* **456**, 605 (2008).
12. B. Kornmann *et al.*, *Science* **325**, 477 (2009); 10.1126/science.1175088.
13. J. R. Friedman, B. M. Webster, D. N. Mastronarde, K. J. Verhey, G. K. Voeltz, *J. Cell Biol.* **190**, 363 (2010).
14. M. West, N. Zurek, A. Hoenger, G. K. Voeltz, *J. Cell Biol.* **193**, 333 (2011).
15. G. K. Voeltz, W. A. Prinz, Y. Shibata, J. M. Rist, T. A. Rapoport, *Cell* **124**, 573 (2006).
16. H. Sesaki, R. E. Jensen, *J. Cell Biol.* **147**, 699 (1999).
17. E. Smirnova, L. Griparic, D. L. Shurland, A. M. van der Bliek, *Mol. Biol. Cell* **12**, 2245 (2001).
18. H. Otera *et al.*, *J. Cell Biol.* **191**, 1141 (2010).
19. Materials and methods are available as supporting material on *Science* Online.

Acknowledgments: This work is supported by NIH grant R01 GM083977 and a Searle Scholar award (to G.K.V.), NIH training grant GM08759 (to J.R.F.), NIH grant R01 GM062942 and an American Heart Innovative Research Grant (to J.N.), and grants from the Biological Sciences Initiative (BURST grant) and the Undergraduate Research Opportunity Program at the University of Colorado (to J.R.D.). We thank the Boulder 3D Electron Microscopy facility for shared equipment and helpful suggestions.

Supporting Online Material

www.sciencemag.org/cgi/content/full/science.1207385/DC1
Materials and Methods
SOM Text
Figs. S1 to S9
References (*20*–*29*)
Movies S1 to S5

22 April 2011; accepted 17 August 2011
Published online 1 September 2011;
10.1126/science.1207385

Antimicrobial Peptides Keep Insect Endosymbionts Under Control

Frédéric H. Login,[1,2] Séverine Balmand,[1,2] Agnès Vallier,[1,2] Carole Vincent-Monégat,[1,2] Aurélien Vigneron,[1,2] Michèle Weiss-Gayet,[2,3] Didier Rochat,[4] Abdelaziz Heddi[1,2]*

Vertically transmitted endosymbionts persist for millions of years in invertebrates and play an important role in animal evolution. However, the functional basis underlying the maintenance of these long-term resident bacteria is unknown. We report that the weevil coleoptericin-A (ColA) antimicrobial peptide selectively targets endosymbionts within the bacteriocytes and regulates their growth through the inhibition of cell division. Silencing the *colA* gene with RNA interference resulted in a decrease in size of the giant filamentous endosymbionts, which escaped from the bacteriocytes and spread into insect tissues. Although this family of peptides is commonly linked with microbe clearance, this work shows that endosymbiosis benefits from ColA, suggesting that long-term host-symbiont coevolution might have shaped immune effectors for symbiont maintenance.

Cooperative associations between animals and symbiotic bacteria are widespread in nature and common in insects that exploit unusually restricted nutritional resources (*1*). In many insects, intracellular bacteria (endosymbionts) are transmitted vertically and provide nutrient supplementation to their hosts, thereby improving their adaptive traits and their invasive power (*2*–*4*).

However, maintaining the beneficial nature of this long-term relationship requires both the host and the symbiont to constrain adaptive interactions. Genomic and evolutionary data have shown that major deletions and mutations of genes occur in endosymbionts, some of which are involved in bacterial virulence and host tolerance (*5*–*7*). Data on how host immune systems have evolved to tolerate cooperative bacteria remain scarce and are mainly limited to extracellular associations with environmental and/or horizontal symbiont transmission (*8*, *9*).

To protect permanent endosymbionts from the host's systemic immune response, and prevent competition with opportunistic invaders, symbionts are sequestered in bacteria-bearing host cells, called the bacteriocytes, which, in some species, group together to form a bacteriome (*10*). To investigate the immune specificities of bacteriocytes, we have studied associations with *Sitophilus*

[1]INSA-Lyon, INRA, UMR203 BF2I, Biologie Fonctionnelle Insectes et Interactions, F-69621 Villeurbanne, France. [2]Université de Lyon, F-69003 Lyon, France. [3]Université Lyon 1, CNRS UMR5534, Centre de Génétique et de Physiologie Moléculaire et Cellulaire, F-69622 Villeurbanne, France. [4]INRA, Université Pierre et Marie Curie, UMR1272 Physiologie de l'Insecte Signalisation et Communication, F-78026 Versailles, France.

*To whom correspondence should be addressed. E-mail: abdelaziz.heddi@insa-lyon.fr
