# Supplementary material for: Resveratrol reduces the apoptosis induced by cigarette smoke extract by upregulating MFN2
Source: PLoS One. 2017 Apr 13;12(4):e0175009. doi: 10.1371/journal.pone.0175009 (PMC5391199; doi:10.1371/journal.pone.0175009)
Supplement: S4 Text — (PDF) [file pone.0175009.s007.pdf]

Acta Pharmacologica Sinica (2014) 35: 779–791
© 2014 CPS and SIMM All rights reserved
1671-4083/14 $32.00
www.nature.com/aps

Original Article

Vam3, a resveratrol dimer, inhibits cigarette smoke-induced cell apoptosis in lungs by improving mitochondrial function

Ling-ling XUAN[1], Ji SHI[1, 2], Chun-suo YAO[1], Jin-ye BAI[1], Feng QU[1], Jin-lan ZHANG[1], Qi HOU[1, *]

[1]State Key Laboratory of Bioactive Substances and Functions of Natural Medicines, Institute of Materia Medica, Chinese Academy of Medical Sciences and Peking Union Medical College, Beijing 100050, China; [2]Institute of Radiation Medicine, Chinese Academy of Medical Sciences and Peking Union Medical College, Key Laboratory of Molecular Nuclear Medicine, Tianjin 300192, China

Aim: To investigate the effects of Vam3 (a resveratrol dimer extracted from Vitis amurensis Rupr) on cigarette smoke (CS)-induced cell apoptosis in lungs *in vitro* and *in vivo* and the underlying mechanisms of action.
Methods: Human bronchial epithelial cell line BEAS-2B was exposed to cigarette smoke condensate (CSC, 300 mg/L), and cell apoptosis was determined using flow cytometry and Hoechst staining. Mitochondrial membrane potential was examined with TMRE staining. ROS and ceramide levels were detected with DCFH-DA fluorescence and HPLC-MS/MS, respectively. Cytochrome *c* release was detected using immunofluorescence. Caspase-9 and neutral sphingomyelinase 2 expression was measured with Western blotting. The breast carcinoma cell line MCF7 stably expressing GFP-tagged Bax was used to elucidate the role of mitochondria in CS-induced apoptosis. For *in vivo* study, male mice were exposed to CS for 5 min twice a day for 4 weeks. The mice were orally administered Vam3 (50 $mg \cdot kg^{-1} \cdot d^{-1}$) or resveratrol (30 $mg \cdot kg^{-1} \cdot d^{-1}$) each day 1 h before the first CS exposure.
Results: Pretreatment of BEAS-2B cells with Vam3 (5 μmol/L) or resveratrol (5 μmol/L) significantly suppressed CSC-induced apoptosis, and prevented CSC-induced Bax level increase in the mitochondria, mitochondrial membrane potential loss, cytochrome *c* release and caspase-9 activation. Furthermore, pretreatment of BEAS-2B cells with Vam3 or resveratrol significantly suppressed CSC-stimulated intracellular ceramide production, and CSC-induced upregulation of neutral sphingomyelinase 2, the enzyme responsible for ceramide production in bronchial epithelial cells. Similar results were obtained in C6-pyridinium ceramide-induced apoptosis of GFP-Bax-stable MCF7 cells *in vitro*, and in the lungs of CS-exposed mice that were treated with oral administration of Vam3 or resveratrol.
Conclusion: Vam3 protects bronchial epithelial cells from CS-induced apoptosis *in vitro* and *in vivo* by preventing mitochondrial dysfunction.

Keywords: Vam3; resveratrol; lung; bronchial epithelial cell; cigarette smoking; apoptosis; Bax; mitochondria; ceramide; sphingomyelinase

Acta Pharmacologica Sinica (2014) 35: 779–791; doi: 10.1038/aps.2014.17; published online 21 Apr 2014

Introduction

Chronic obstructive pulmonary disease (COPD) is predicted to become the third leading cause of mortality, and has become one of the major causes of medical expenditure worldwide[1]. Cigarette smoke (CS), a complex mixture of oxidants, free radicals, nicotine, and chemicals, induces respiratory system oxidative stress, cell death and senescence. Many studies have shown that apoptotic epithelial and endothelial cells are increased in the lungs of COPD patients and mice exposed to CS[2–5]. Therefore, protecting airway structural cells, such as epithelial cells and endothelial cells, from apoptosis is extremely important in the treatment of COPD.

Ceramide is a second messenger that modulates epithelial cell oxidative stress and apoptosis[6, 7]. Cell-permeable ceramide analogs are able to elicit a direct effect on mitochondria, ranging from the inhibition of the respiratory chain and the enhancement of reactive oxygen species (ROS) generation to the induction of mitochondrial permeability transition[8]. Several studies have reported that ceramide causes mitochondrial inner membrane potential collapse and cytochrome *c* release from the mitochondria[9–11], two events characteristic of ceramide-induced apoptosis. Exposure to CS leads to

* To whom correspondence should be addressed.
E-mail houq@imm.ac.cn
Received 2013-11-5 Accepted 2014-02-20

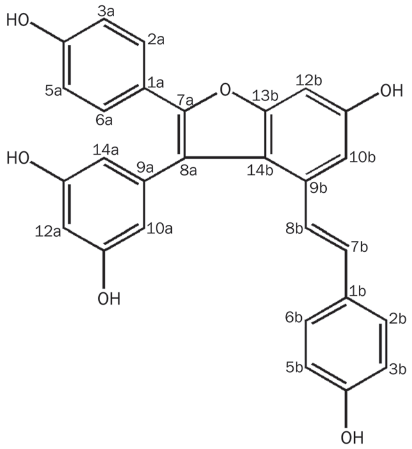

Figure 1. Chemical structure of Vam3.

ceramide accumulation in lung epithelial cells in both humans and rodents[7]. Filosto *et al* showed that increased ceramide production induced the apoptosis of human bronchial epithelial 1 and adenocarcinomic human alveolar basal epithelial cells (A549) exposed to H_2O_2 or CS, and in these cells, ceramide generation is upstream of the caspase cascades[2].

Ceramide is generated through *de novo* synthesis or the hydrolysis of sphingomyelin by sphingomyelinase (SMase). Although endogenous ceramide is produced by increased *de novo* synthesis, in most cases, SMase is also inducible[12]. Several types of SMase have been identified by their pH optima of action: neutral sphingomyelinase (nSMase), acidic sphingomyelinase (aSMase), and alkaline SMase[13]. In lung epithelial cells, the ROS component of CS specifically activates nSMase2, increases ceramide formation via the hydrolysis of sphingomyelin, and subsequently promotes pathological apoptosis[2]. Because ceramide is an upstream mediator of oxidative stress and apoptosis, compounds that regulate ceramide production and ceramide-induced apoptosis might be a potential therapy for COPD.

Vam3 is a resveratrol dimer derived from *Vitis amurensis* Rupr, which grows in northeastern and central China. Its roots and stems have been used in traditional Chinese medicines for hundreds of years. Our previous *in vivo* and *in vitro* studies demonstrated that the oral administration of Vam3 had anti-asthmatic effects and attenuated ovalbumin-induced lung tissue damage[14, 15]. Vam3 also inhibits autophagy in cigarette smoke condensate (CSC)-treated human bronchial epithelial cells (BEAS-2B) and CS-exposed mouse lungs[16]. In the present study, human breast carcinoma cells (MCF7) stably expressing GFP-tagged Bax, a tool to examine the effect of reagents on Bax translocation and other apoptotic responses, were used as screening cells to determine whether Vam3 had anti-apoptotic effects. Second, we determined whether Vam3 had anti-apoptotic effects in the BEAS-2B cell line; bronchial epithelial cells are known to be implicated in pulmonary emphysema in COPD. Finally, the effect of Vam3 on CS exposure-induced lung injury, the most common cause of COPD, was studied further in mice.

Materials and methods

Materials

Human breast carcinoma cells (MCF7) and BEAS-2B cells were obtained from the American Type Culture Collection (ATCC, Rockville, MA, USA). Tetramethylrhodamine ethyl ester (TMRE), Hoechst 33258, 2,7'-dichlorofluorescin diacetate (DCFH-DA), and *N*,*N'*-*Bis*[4-(4,5-dihydro-1*H*-imidazol-2-yl)phenyl]-3,3'-*p*-phenylene-*bis*-acrylamide dihydrochloride (GW4869) were purchased from Sigma-Aldrich (St Louis, MO, USA). M199 medium and Dulbecco's modified Eagle's medium (DMEM) were obtained from Gibco (Grand Island, NY, USA). Fetal bovine serum (FBS) was from HyClone (Logan, UT, USA). Anti-cytochrome *c* and anti-nSMase2 antibodies were purchased from Santa Cruz Biotechnology (Santa Cruz, CA, USA). Anti-caspase-9, anti-Bax, and anti-β-actin antibodies were from Cell Signaling Technology (Beverly, MA, USA). Anti-rabbit IgG secondary antibody, anti-mouse IgG secondary antibody, fluorescein isothiocyanate (FITC)-labeled goat anti-mouse secondary antibody, and rhodamine (TRITC)-labeled goat anti-mouse secondary antibody were from Zhong Shan Golden Bridge Biotechnology (Beijing, China). The pan-caspase inhibitor zVAD-fmk was purchased from Alexis Biochemical (Lausen, Switzerland). C_6-pyridinium ceramide (LCL29) was from Avanti Polar Lipids (Alabaster, AL, USA). Trizol was purchased from Invitrogen Corporation (California, USA). UltraSYBR Mixture (with Rox) was from Kangwei Biotechnology (Beijing, China). Vam3 was prepared as previously described[16]. In the preliminary experiments testing the dose effect of Vam3 on apoptosis (10, 5, 1, and 0.1 μmol/L), 10 μmol/L had severe cytotoxic effects on both MCF7 and BEAS-2B cells and at 0.1 μmol/L had little effect on C_6-pyridinium ceramide and CSC-induced apoptosis; therefore, 5 and 1 μmol/L were selected for the present study.

Cell culture

MCF7 and BEAS-2B cells were routinely maintained at 37 °C in an atmosphere of 5% CO_2 in DMEM supplemented with 10% FBS and M199 medium supplemented with 10% FBS, respectively.

Cigarette smoke condensate preparation

CSC was prepared from commercial cigarettes (15 mg tar, 1.2 mg nicotine per cigarette; Honghe, China) as previously described[16]. Briefly, one cigarette was combusted with a "water-pipe" smoking device that drew the smoke into a flask submerged in liquid nitrogen. The CSC was prepared by dissolving the collected smoke particulates in dimethyl sulfoxide (DMSO) and frozen at -80 °C until use.

Apoptosis detection with Annexin V/PI

MCF7 and BEAS-2B cells were seeded in 6-well plates at a density of 3×10^5 cells/well. Cells treated with drugs or the diluent as designed in the experiment were rinsed with warm 1×phosphate-buffered saline (PBS) (37 °C) before further diges-

tion. The cell suspensions were washed twice with ice-cold PBS and stained using an Annexin V/PI staining kit according to the manufacturer's instructions (Beyotime Institute of Biotechnology, Beijing, China) and then analyzed with a flow cytometer (Partec GmbH, Münster, Germany) to determine the proportion of apoptotic cells.

Hoechst staining for examining nuclear condensation and fragmentation

After treatment with drugs or the diluent as designed in the experiment, the cells were washed twice with 1×PBS and fixed in methanol at room temperature for 15 min and then in ice-cold methanol at -20 °C for 15 min. The cells were then washed twice with 1×PBS before incubation with 10 µg/mL Hoechst 33258 for 30 min at 37 °C. After washing, the cells were observed with a fluorescence microscope (Olympus Optical, Tokyo, Japan), and images were captured with a digital camera (Kodak, NY, USA).

Bax translocation analysis

GFP-Bax-stable MCF7 cells were plated in 96-well plates. The cells were pretreated with 5 or 1 µmol/L Vam3 or the diluent for 2 h before incubation with 10 µmol/L C_6-pyridinium ceramide or the diluent for 18 h. The cells were then visualized using a fluorescence microscope, and the percentages of GFP-Bax punctate cells were averaged from three separate visual fields as described previously[17].

Mitochondrial membrane potential assessment by TMRE staining

TMRE was used to label active mitochondria and to determine the mitochondrial membrane potential based on the fluorescence intensity of the TMRE sequestered by the mitochondria[18]. Briefly, MCF7 and BEAS-2B cells were plated in 96-well plates with black walls and clear bottoms. The cells were pretreated with 5 or 1 µmol/L Vam3 or the diluent for 2 h before incubation with 10 µmol/L C_6-pyridinium ceramide, 300 mg/L CSC or the diluent for the indicated time. The cells were then incubated with 400 nmol/L TMRE for 1 h at 37 °C in the dark. After two brief washes with warm 1×PBS (37 °C), the fluorescence intensity produced by the TMRE was measured at an excitation wavelength of 549 nm and an emission wavelength of 575 nm using a fluorescence plate reader (BioTek, Winooski, VT, USA). The TMRE fluorescence intensity was normalized as the percentage of each treatment group over the control.

Measurement of ROS production

We used a cell-based assay for measuring ROS including hydroxyl, peroxyl, or other reactive oxygen species activity within the cells. Briefly, MCF7 cells were pretreated with 5 or 1 µmol/L Vam3 or the diluent for 2 h before incubation with 10 µmol/L C_6-pyridinium ceramide or the diluent for 4 h. Then, the cells were incubated with a cell-permeable DCFH-DA probe at a final concentration of 10 µmol/L at 37 °C for 30 min in the dark. After three washes with 1×PBS, the fluorescence intensity was measured to determine the ROS levels within the cytosol at an emission wavelength of 525 nm and an excitation wavelength of 488 nm using a fluorescence microplate reader (BioTek, Winooski, VT, USA).

Western blotting

The protein extracts, *ie*, Bax from the cytosol and mitochondria, procaspase-9, cleaved caspase-9, and nSMase2, were separated on a 10%-15% SDS-polyacrylamide gel and transferred onto PVDF membranes. After blocking with 5% nonfat milk for 2 h, the membranes were incubated with specific primary antibodies overnight at 4 °C. After three washes, the membranes were incubated with secondary antibodies at the appropriate dilutions for 1 h at room temperature. The labeled protein bands were visualized using an enhanced chemiluminescent (ECL) substrate kit (Tiangen Biotech, Beijing, China). The density of each band was quantified by QuantiScan Version 11 (Biosoft, Cambridge, UK).

Immunofluorescence for detecting cytoplasmic cytochrome *c*

The cells were fixed in 4% paraformaldehyde at room temperature for 15 min and permeabilized with 1% Triton in 1×PBS for 20 min. After three washes, the cells were incubated with blocking buffer (1×PBS, 10% fetal bovine serum) at room temperature for 30 min. The cells were then incubated with an anti-cytochrome *c* antibody at 4 °C overnight. After washing, the cells were incubated with TRITC or a FITC-labeled goat anti-mouse secondary antibody for 2 h. The cells were then washed with 1×PBS and visualized with a fluorescence microscope (Olympus, Optical, Tokyo, Japan). The images were captured with a digital camera (Kodak, NY, USA).

Effects of Vam3 in an animal model of cigarette smoke exposure

All experiments were performed in accordance with the institutional guidelines at the Experimental Animal Center of the Institute of Materia Medica, Beijing, China. Specific pathogen free (SPF) male BALB/c mice (18–20 g, Beijing HFK Bioscience Co, Ltd, Beijing, China) were randomly divided into four groups (*n*=8): the naive normal control group was exposed to air and pretreated with the drug diluent; the Vam3 group was exposed to CS and pretreated with 50 mg/kg Vam3; the resveratrol (RES) group was exposed to CS and pretreated with 30 mg/kg RES; and the CS exposure group was exposed to CS and pretreated with the drug diluent as the CS control. These animals were exposed to CS for 5 min twice a day for 4 weeks as previously described[16]. One hour before the first CS exposure on each day, the animals were intragastrically administered Vam3, RES or the diluent as indicated above. At the end of the experiment, all of the animals were sacrificed for a terminal deoxynucleotidyl transferase dUTP nick end labeling (TUNEL) assay for the detection of lung epithelial cell apoptosis, lung tissue ceramide measurement and nSMase2 expression evaluation.

Terminal deoxynucleotidyl transferase dUTP nick end labeling (TUNEL) assay for detecting apoptosis in lung epithelial cells

The lungs were inflated with 10% buffered formalin. The

apoptotic cells were detected using a TUNEL assay kit (Beyotime Institute of Biotechnology, Haimen, China) on paraffin-embedded lung tissue specimens according to the manufacturer's instructions. Briefly, the apoptotic cells in the lung slices were visualized by labeling DNA strand breaks with Biotin-coupled dUTP in the presence of terminal deoxynucleotidyl-transferase. The slides were then incubated with streptavidin-HRP, washed in 1×PBS, and then stained with a diaminobenzidine (DAB) solution at room temperature. TUNEL positive stained cells were detected using a light microscope at a 200×magnification. The TUNEL-stained cells expressed as a percentage of the total cells were counted in 5 visual fields on each slide, with 4 slides in each group.

Immunohistochemistry

Immunohistochemistry was performed as previously described[16]. Paraffin-embedded specimens were deparaffinized in xylene, rehydrated through graded alcohols, and rinsed in PBS. Endogenous peroxidase activity was blocked with 3% H_2O_2 for 10 min at room temperature. Antigen retrieval was performed by placing the sections in an EDTA-antigen retrieval buffer in a microwave. The sections were blocked with sheep serum for 10 min and then incubated with an anti-Bax antibody at 4 °C overnight. The PV-9000 kit (Zhong Shan Golden Bridge Biotechnology Co, Ltd, Beijing, China) was applied to each section, according to the manufacturer's instructions. The stain was developed using an ABC kit (Zhong Shan Golden Bridge Biotechnology Co, Ltd, Beijing, China). The sections were dehydrated through graded alcohol and mounted with neutral gum. The sections were observed under a microscope. The assessment of the immunostaining intensity was performed using Image-Pro Plus 6.0 software. The antigen spot color intensity was expressed as the mean integrated optical density (IOD).

Ceramide measurement

Ceramide accumulation induces apoptosis, and its intracellular level has been used as an indicator of apoptosis. The intracellular ceramide contents were determined as described by Qu *et al*[19]. Briefly, the cells or lung tissue homogenates from each group were harvested, and the lipids were extracted. High performance liquid chromatography-tandem mass spectrometry (HPLC-MS/MS) analysis was performed to detect the levels of ceramide with different structures using an Agilent 6410B Triple Quad mass spectrometer (Agilent Technologies, Inc, Santa Clara, CA, USA).

Real-time qPCR analysis

Total RNA was extracted using Trizol according to the manufacturer's instructions. First-strand cDNA was synthesized using a ReverTra Ace qPCR RT Master Mix with a gDNA Remover Kit (TOYOBO, Osaka, Japan). Real-time qPCR was performed using an UltraSYBR Mixture (with Rox). The relative levels of gene expression were determined with β-actin as the control. The primer sequences were as follows: murine nSMase2 (forward, 5'-TCTACCTCCTCGACCAGCAC-3'; reverse, 5'-TGCTGCTCCAGTTTGTCATC-3'), human nSMase2 (forward, 5'-AGGACTGGCTGGCTGATTTC-3'; reverse, 5'-TGTCGTCAGAGGAGCAGTTATC-3'), murine β-actin (forward, 5'-TGACAGGATGCAGAAGGAGA-3'; reverse, 5'-CGCTCAGGAGGAGCAATG-3'), and human β-actin (forward, 5'-GAGCTACGAGCTGCCTGACG-3'; reverse, 5'-GTAGTTTCGTGGATGCCACAG-3').

Statistical analysis

The results are expressed as the mean±SD from at least three independent experiments. The statistical analysis was performed using one-way ANOVA to determine changes between the groups. A *P* value of 0.05 or less was considered statistically significant.

Results

Vam3 counteracted C_6-pyridinium ceramide-induced apoptosis in MCF7 cells

In this experiment, MCF7 cells were labeled with Annexin V/PI. The number of apoptotic cells in each treated group was detected using flow cytometry and expressed as the fold change over the control group treated with diluent only. Compared with the MCF7 cells treated with the diluent only (control), the C_6-pyridinium ceramide treatment alone increased the proportion of apoptotic cells to 5.53±1.23-fold of the control (P=0.003). Compared with the cells treated with C_6-pyridinium ceramide alone, pretreatment with 5 and 1 μmol/L Vam3 reduced the apoptotic cells to 2.07±0.14-fold and 2.66±0.63-fold of the control, respectively (P<0.05 for both) (Figure 2A). Vam3 at 5 μmol/L had a greater effect than 1 μmol/L but did not reach statistical significance (P=0.191). Thus, Vam3 inhibited C_6-pyridinium ceramide-induced apoptosis.

Hoechst 33258 staining of the nuclei also showed the protective effects of Vam3 on C_6-pyridinium ceramide-induced apoptosis. Compared with the MCF7 cells treated with diluent only (control), the C_6-pyridinium ceramide treatment increased the number of cells with condensed or fragmented nuclei. Compared with the cells treated with C_6-pyridinium ceramide alone, pretreatment with 5 and 1 μmol/L Vam3 decreased the number of cells with apoptotic nuclei, though the former appeared to have had a greater effect (Figure 2B).

Vam3 alleviated C_6-pyridinium ceramide-induced apoptosis through the mitochondrial pathway

Bax translocation from the cytoplasm to the mitochondria is one of the key steps in cellular apoptosis. Our previous study showed C_6-pyridinium ceramide mediated Bax translocation to the mitochondria in GFP-Bax-stable MCF7 cells[20]. Here, we investigated the effects of Vam3 on the C_6-pyridinium ceramide-induced subcellular distribution of Bax. Fluorescence microscopy results indicated that in GFP-Bax-stable MCF7 cells treated with diluent only (control), almost all of the cells had Bax localized in the cytoplasm. When C_6-pyridinium ceramide was added to these cells, GFP-Bax shifted from a diffuse cytoplasmic pattern to a punctate membrane-bound state,

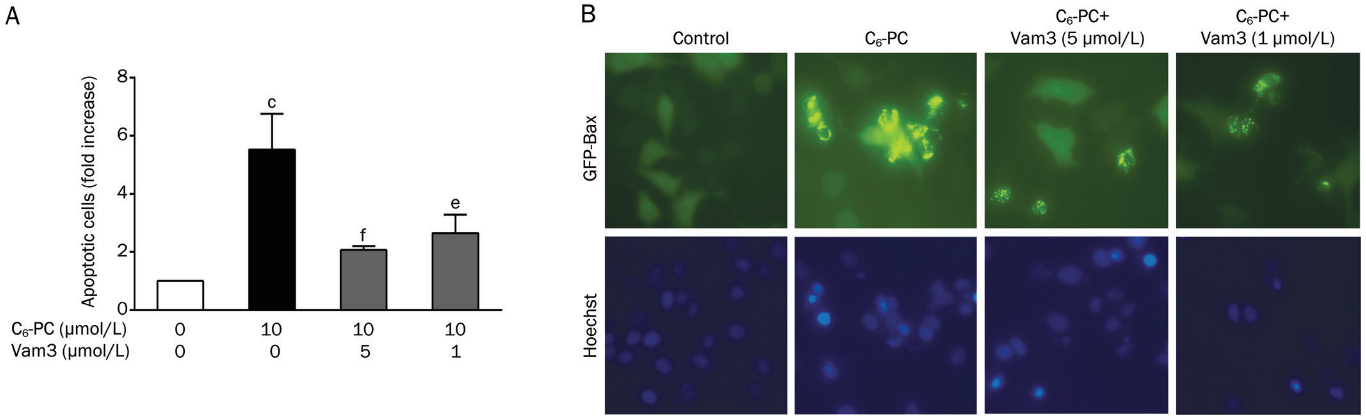

Figure 2. The effects of Vam3 on C_6-pyridinium ceramide (C_6-PC)-induced apoptosis in MCF7 cells. Panel (A) MCF7 cells were pretreated with Vam3 (0, 5, or 1 μmol/L) for 2 h before challenge with 10 μmol/L C_6-PC or the diluent as a control for 18 h. The cells were then labeled with Annexin V/PI and subjected to flow cytometry analysis. The fold change in apoptotic cells over the control was determined (*n*=3 for each group). (B) GFP-Bax-stable MCF7 cells were pretreated with Vam3 (0, 5, or 1 μmol/L) for 2 h before challenge with 10 μmol/L C_6-PC or the diluent as a control for 18 h. The cells were then stained with Hoechst 33258 nuclear stain and examined using a fluorescence microscope to detect apoptotic nuclei. (C) $^{c}P<0.01$ *vs* the control. $^{e}P<0.05$, $^{f}P<0.01$ *vs* the C_6-PC challenge alone.

consistent with our previous study[20]. Pretreatment with 5 and 1 μmol/L Vam3 reduced the C_6-pyridinium ceramide-induced GFP-Bax shift, while 5 μmol/L had a greater effect than did 1 μmol/L (Figure 3A). Consistent with the fluorescence microscopy results, C_6-pyridinium ceramide increased the percentage of GFP-Bax punctate cells to 50.13%±3.10% compared with the control (7.63%±6.62%, *P*<0.001), while pretreatment with 5 and 1 μmol/L Vam3 reduced the percentages of GFP-Bax punctate cells to 24.53%±5.93% and 35.27%±2.25%, respectively (*P*=0.003 compared with the C_6-pyridinium ceramide treatment alone for both doses), and the former had a greater effect than the latter (*P*=0.043) (Figure 3B).

In addition, compared with the GFP-Bax-stable MCF7 cells treated with diluent only, Western blot results showed that both cytosolic and mitochondrial Bax increased when treated with C_6-pyridinium ceramide alone, but mitochondrial Bax increased to a greater extent. However, compared with the cells treated with C_6-pyridinium ceramide alone, pretreatment with 5 and 1 μmol/L Vam3 barely increased the cytosolic Bax expression but significantly reduced the mitochondrial Bax expression, and 5 μmol/L had a greater effect than 1 μmol/L (Figure 3C). All of these data indicated that Bax translocated to the mitochondria from the cytosol following treatment with C_6-pyridinium ceramide, while Vam3 could reverse, at least partially, the C_6-pyridinium ceramide-induced Bax redistribution.

Mitochondria play a central role in the initiation and activation of apoptosis, and Bax plays an essential role in the onset of mitochondrial dysfunction[21]. The mitochondrial membrane potential, determined by TMRE staining (Figure 3D), and shown as the TMRE-fluorescence intensity percentage over control (%), decreased to 63.16%±2.52% of the control (*P*<0.001) after a 6 h exposure to C_6-pyridinium ceramide. Pretreatment with 5 and 1 μmol/L Vam3 increased the TMRE-fluorescence intensity to 87.72%±5.03% and 72.14%±4.97% of the control, respectively (*P*<0.001 and *P*=0.018 compared with the C_6-pyridinium ceramide treatment alone for 5 and 1 μmol/L, respectively); 5 μmol/L had a greater effect than 1 μmol/L (*P*=0.005). Vam3 treatment at 0, 1, or 5 μmol/L for 18 h had no significant effect on Bax translocation or the mitochondrial membrane potential in GFP-Bax-stable MCF7 cells (*n*=3 for each dose group) (data not shown).

Mitochondria are sensitive to changes in the cellular redox status, and ROS activation induces the disruption of the mitochondrial membrane potential and the release of cytochrome *c* from the mitochondria into the cytoplasm[22]. Therefore, we examined the production of ROS in MCF7 cells, expressed as the fluorescence intensity percentage over control (%), using DCFH-DA, a cell-permeable fluorogenic oxidation-sensitive fluorescent dye. C_6-pyridinium ceramide treatment alone increased the intracellular DCFH-DA fluorescence to 313.83%±63.20% of the control (*P*<0.001). Pretreatment with 5 and 1 μmol/L Vam3 reduced the C_6-pyridinium ceramide-induced ROS production to 110.18%±37.51% and 167.45%±52.65% of the control, respectively (*P*=0.001 and 0.007 compared with C_6-pyridinium ceramide treatment alone for 5 and 1 μmol/L, respectively), although there was no statistical difference between the two doses (Figure 3E).

In the mitochondrial apoptosis pathway, cytochrome *c* is released from the mitochondria to the cytoplasm and subsequently binds with Apaf-1 and activates caspases[23]. In GFP-Bax-stable MCF7 cells, using immunofluorescence labeling with an anti-cytochrome *c* antibody, it was found that cytoplasmic cytochrome *c* became visible (bright yellow in overlay) and that GFP-Bax was localized to the mitochondria (bright green) when treated with C_6-pyridinium ceramide alone. However, adding 5 μmol/L Vam3 significantly attenuated the cytochrome *c* release in the cells with C_6-pyridinium ceramide treatment (Figure 3F).

Caspase activation is a key step in apoptosis. Ceramide has

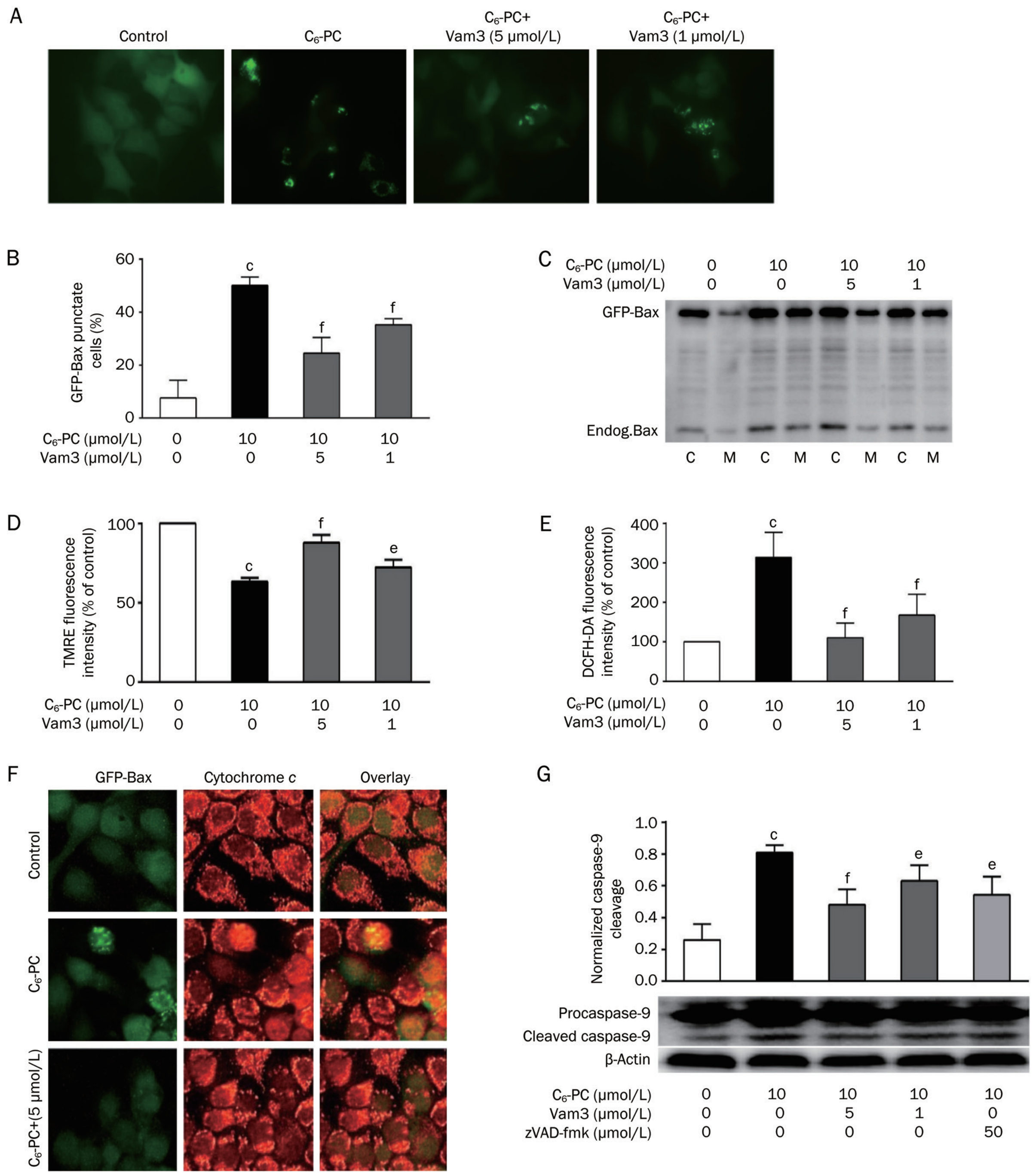

Figure 3. Mitochondrial function changes with pretreatment with Vam3 of C_6-pyridinium ceramide (C_6-PC)-challenged MCF7 cells. (A) GFP-Bax-stable MCF7 cells were pretreated with Vam3 (0, 5, or 1 μmol/L) for 2 h before challenge with 10 μmol/L C_6-PC or the diluent as a control for 18 h. The cells were then visualized using a fluorescence microscope. (B) The percentages of GFP-Bax punctate cells were quantitated from three separate visual fields from fluorescence microscopy photos (*n*=3 for each group) using the same design as in Panel A. (C) Bax expression in the cytoplasm (C) and the mitochondria (M) using Western blot analyses based on the same design as in Panel A. (D) MCF7 cells were pretreated with Vam3 (0, 5, or 1 μmol/L) for 2 h before challenge with 10 μmol/L C_6-PC or the diluent as a control for 6 h. The mitochondrial membrane potential was determined using TMRE staining and expressed as the TMRE fluorescence intensity over control (%) (*n*=3 for each group). (E) MCF7 cells were pretreated with Vam3 (0, 5, or 1 μmol/L) for 2 h before challenge with 10 μmol/L C_6-PC or the diluent as a control for 4 h. The ROS level was detected with a DCFH-DA probe and expressed as the fluorescence intensity percentage over control (%) (*n*=3 for each group). (F) GFP-Bax-stable MCF7 cells were pretreated with Vam3 (0 or 5 μmol/L) for 2 h before challenge with 10 μmol/L C_6-PC or the diluent as a control for 18 h. The cells were then stained with an anti-cytochrome *c* antibody and visualized using a fluorescence microscope. (G) MCF7 cells were pretreated with Vam3 (0, 5, or 1 μmol/L) for 2 h before challenge with 10 μmol/L C_6-PC or the diluent as a control for 18 h. Then, procaspase-9 and cleaved caspase-9 (active form) expression was determined using Western blot analyses. The upper panel is the densitometrically quantitated cleaved caspase-9 expression based on the Western blot analyses (*n*=3 for each group). ^{c}P<0.01 *vs* the control. ^{e}P<0.05, ^{f}P<0.01 *vs* the C_6-PC challenge alone.

been implicated in the induction of cell apoptosis predominantly through a caspase-dependent pathway[12]. Caspase-9 is an initiator caspase; therefore, we used active caspase-9 to determine the caspase pathway activation. C_6-pyridinium ceramide treatment alone increased the level of cleaved caspase-9, the active form of caspase-9, as shown in the expression band and density quantitation. Pretreatment with 5 and 1 μmol/L Vam3 reduced the increased cleaved caspase-9 expression induced by C_6-pyridinium ceramide (P=0.006 and 0.048 compared with the C_6-pyridinium ceramide treatment alone for 5 and 1 μmol/L, respectively), the effect of which was not different from that of zVAD-fmk (P=0.020 compared with the C_6-pyridinium ceramide treatment alone), an irreversible pan-caspase inhibitor (Figure 3G). These data indicated that the Vam3 pretreatment prevented C_6-pyridinium ceramide-induced apoptosis via a mitochondrial pathway.

Vam3 counteracted CSC-induced apoptosis in BEAS-2B cells

In the BEAS-2B cells labeled with Annexin V/PI, the number of apoptotic cells, as detected using flow cytometry, in each treated group is expressed as the fold change over the control group treated with the diluent only. CSC treatment alone increased the apoptotic cells to 5.30±1.30-fold of the control (P=0.005), while the pretreatment with 5 and 1 μmol/L Vam3 reduced the apoptotic cells to 1.64±0.24 and 2.41±0.43-fold of the control (P=0.009 and 0.022 compared with the CSC treatment alone for 5 and 1 μmol/L, respectively), and 5 μmol/L had a greater effect than 1 μmol/L (P=0.05). The effect of Vam3 was not different from that of 5 μmol/L RES (1.82±0.14-fold of control, P=0.010 compared with the CSC treatment alone) (Figure 4A). To confirm the results from the Annexin V/PI assay, using a similar experimental design, BEAS-2B cells were stained with Hoechst 33258 to detect apoptotic nuclei in the cells. Consistent with the results above, the CSC treatment alone caused significant nuclear condensation, and the pretreatment with 5 and 1 μmol/L Vam3 reduced nuclear condensation, similar to the effect of 5 μmol/L RES (Figure 4B).

Vam3 alleviated CSC-induced apoptosis through the mitochondrial pathway

We investigated the effects of Vam3 on the CSC-induced subcellular distribution of Bax. The levels of Bax were determined by Western blotting in the cytosolic and mitochondrial fractions. By 12 h after CSC (300 mg/L) addition, the Bax level had increased in the mitochondrial fraction (Figure 5A). Compared with the challenge with CSC alone, the pretreatment with Vam3 or RES reduced the mitochondrial Bax expression and increased the cytosolic Bax expression, especially at 5 μmol/L, while Vam3 had a greater effect than RES (Figure 5A). Therefore, Vam3 treatment prior to CSC exposure inhibited Bax redistribution in BEAS-2B cells.

TMRE staining was performed to determine the effect of Vam3 on the CSC-induced mitochondrial membrane potential loss. After incubating the cells with 300 mg/L CSC for 12 h, the TMRE-fluorescence intensity decreased to 58.76%±13.36% of the control (P<0.01). Pretreatment with Vam3 (5 or 1 μmol/L) or RES increased the TMRE-fluorescence intensity to 89.06%±8.04%, 75.56%±7.29% and 83.97%±14.41% of the control, respectively (P=0.006, P=0.068 and P=0.05 compared with the CSC treatment alone for 5 μmol/L Vam3 or 1 μmol/L Vam3 and RES, respectively); 5 μmol/L Vam3 had a greater effect than 1 μmol/L (P<0.01) (Figure 5B). Vam3 treatment at 0, 1, or 5 μmol/L for 18 h had no significant effect on Bax translocation or the mitochondrial membrane potential in BEAS-2B cells (n=3 for each dose group) (data not shown).

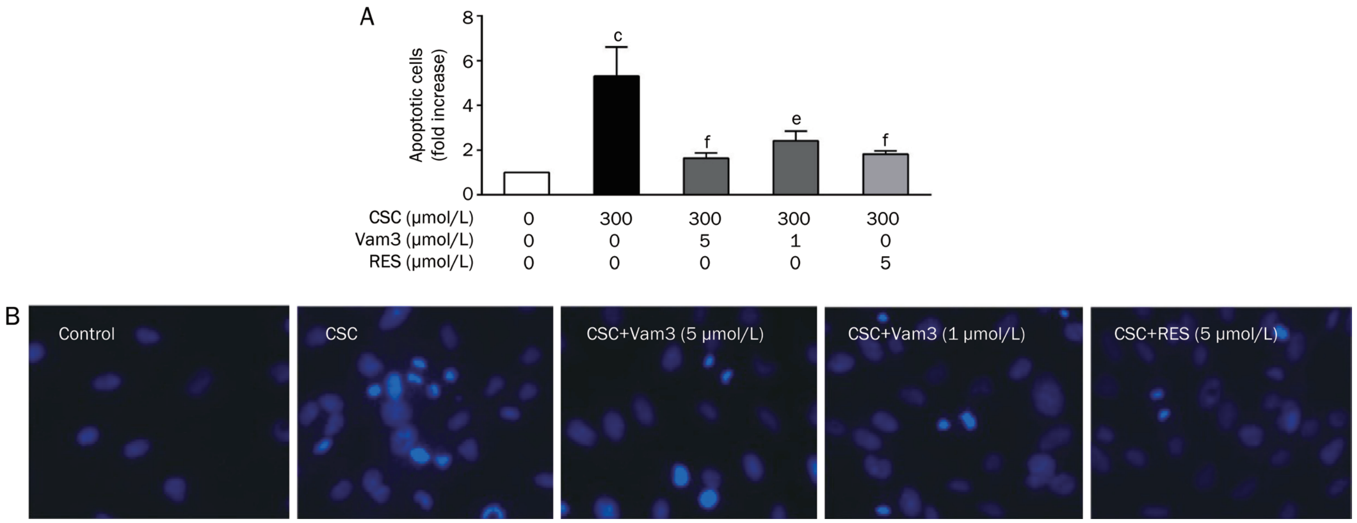

Figure 4. The effects of Vam3 on apoptosis in cigarette smoke condensate (CSC)-challenged BEAS-2B cells. (A and B) BEAS-2B cells were pretreated with Vam3 (0, 5, or 1 μmol/L) or resveratrol (RES) (5 μmol/L) for 2 h before exposure to 300 mg/L CSC or the diluent as a control for 12 h. (A) The cells were labeled with Annexin V/PI and analyzed using flow cytometry analysis. The results were expressed as the fold change of apoptotic cells over the control (n=3 for each group). (B) Using the same design as in Panel A, the cells stained with Hoechst 33258 nuclear stain were examined using a fluorescence microscope to detect apoptotic nuclei. ^{c}P<0.01 compared with the control. ^{e}P<0.05, ^{f}P<0.01 compared with the CSC challenge alone.

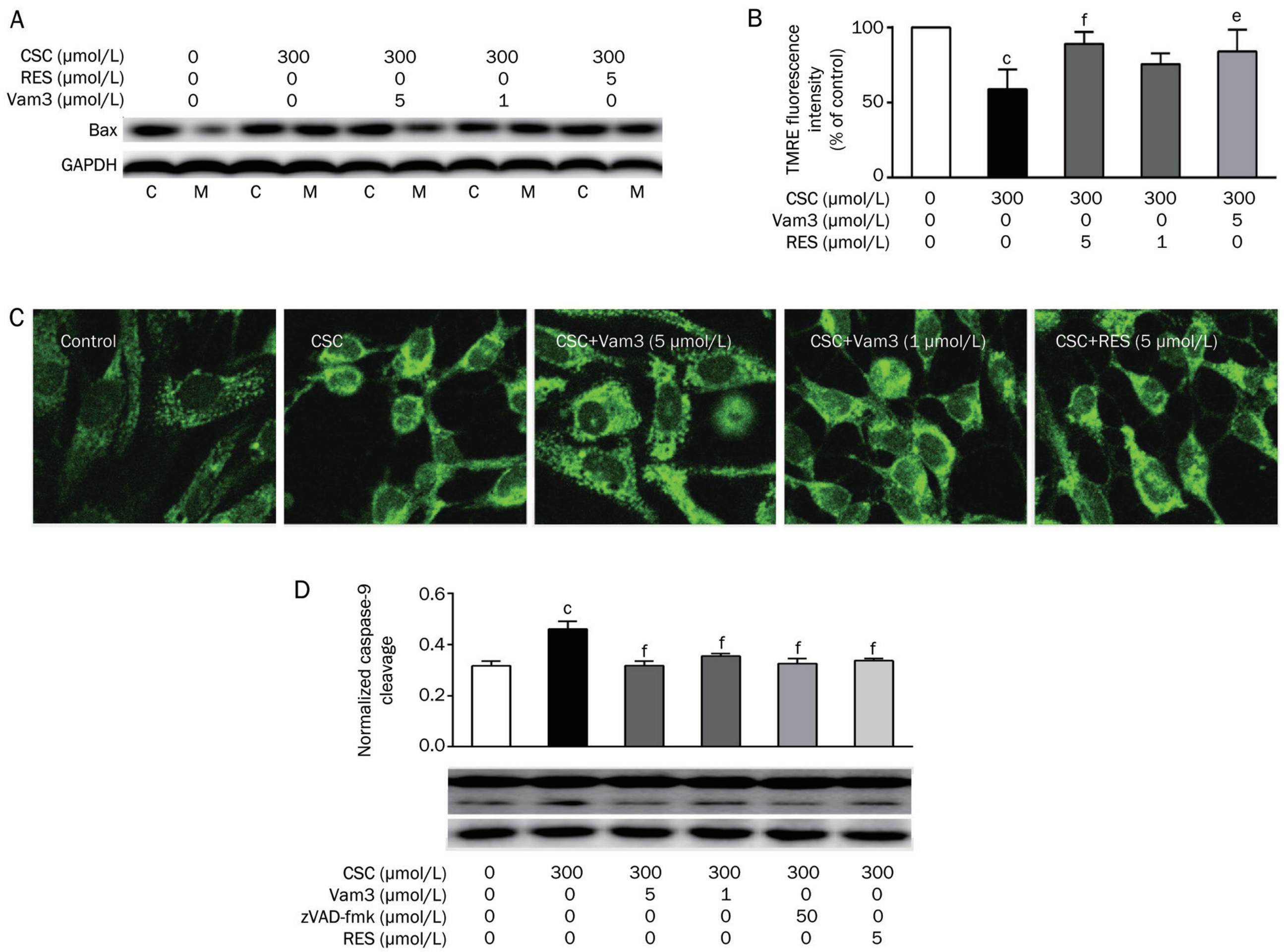

Figure 5. Mitochondrial function changes with Vam3 pretreatment of CSC-challenged BEAS-2B cells. BEAS-2B cells were pretreated with Vam3 (0, 5, or 1 μmol/L) for 2 h before challenge with 300 mg/L CSC or the diluent as a control for 12 h. (A) Bax expression in the cytoplasm (C) and the mitochondria (M) was determined using Western blot analyses. (B) The mitochondrial membrane potential was determined using TMRE staining and expressed as the TMRE fluorescence intensity over the control (%) (*n*=3 for each group). (C) The cells were stained with an anti-cytochrome *c* antibody and visualized using a fluorescence microscope. (D) Procaspase-9 and cleaved caspase-9 (active form) expression was determined using Western blot analyses. The upper panel is the densitometrically quantitated cleaved caspase-9 expression based on the Western blot analysis (*n*=3 for each group). [c]*P*<0.01 *vs* the control. [e]*P*<0.05, [f]*P*<0.01 *vs* the CSC challenge alone.

Cytochrome *c* release was examined by immunofluorescence. Exposure to CSC induced a significant cytochrome *c* release, as shown by a diffuse cytosolic cytochrome *c* staining. Vam3 and RES pretreatment prevented the induction of the diffuse cytochrome *c* staining, while the effect at 5 μmol/L was greater than at 1 μmol/L (Figure 5C).

The cleavage of caspase-9 was determined by Western blotting. CSC treatment induced the activation of caspase-9 in BEAS-2B cells, as shown in the expression band and density quantitation. Pretreatment with Vam3 (5 or 1 μmol/L), RES (5 μmol/L) or zVAD-fmk (50 μmol/L) significantly reduced CSC-induced caspase-9 activation, while the effect at 5 μmol/L was greater than at 1 μmol/L (*P*=0.029) (Figure 5D). Therefore, Vam3 pretreatment prevented CSC-induced apoptosis via the mitochondrial pathway.

Vam3 attenuated apoptosis in mouse lungs exposed to CS through the mitochondrial pathway

To test the inhibitory effects of Vam3 on apoptosis in lungs *in vivo*, we performed a TUNEL assay on lungs from the normal control, CS exposure, Vam3, and RES groups. The apoptotic lung epithelial cells significantly increased to 60.25%±18.01% in the CS-exposed group compared with the normal control group (1.87%±0.42%, *P*=0.002 compared with air exposure alone), while the pretreatment with 50 mg/kg Vam3 decreased the number of apoptotic lung epithelial cells caused by the CS exposure to 9.45%±1.53% (*P*=0.003 compared with the CS exposure alone), the effect of which was more than that of 30 mg/kg RES (21.83%±2.83%, *P*=0.012 compared with the CS exposure alone) (*P*=0.003 comparing the effect of Vam3 *vs* RES) (Figure 6A and 6B). The level of Bax and the activation

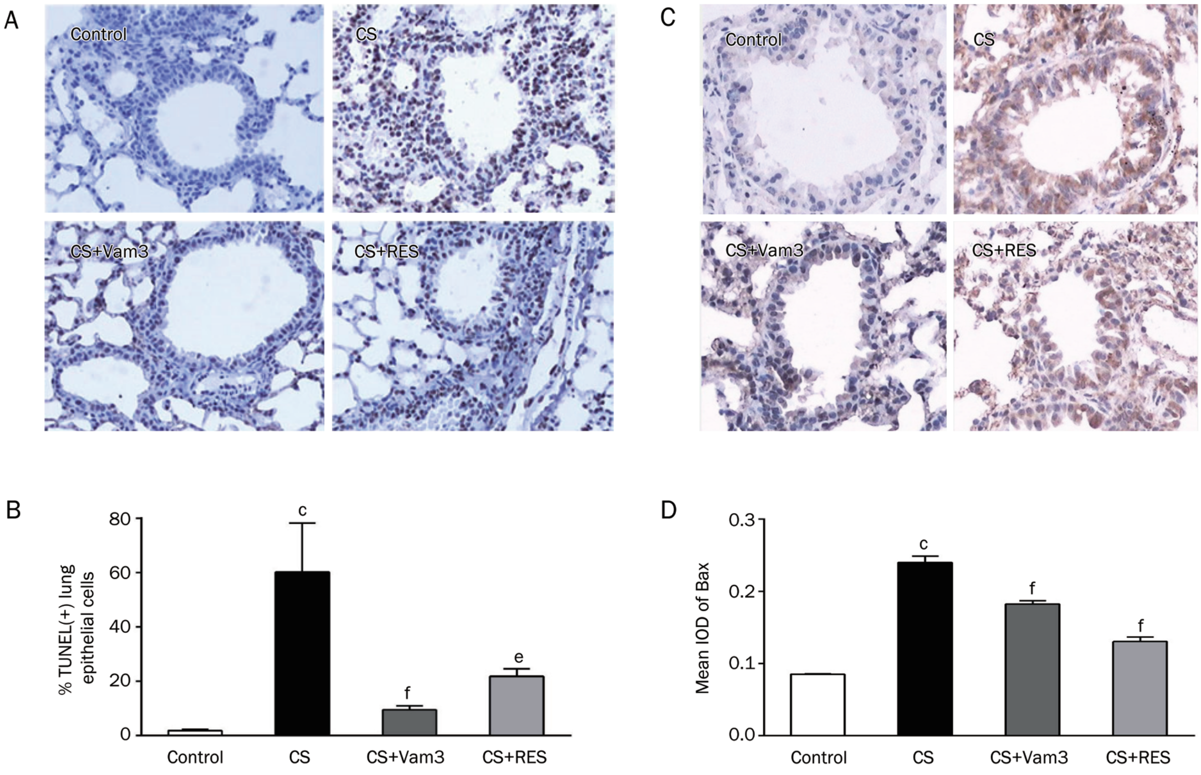

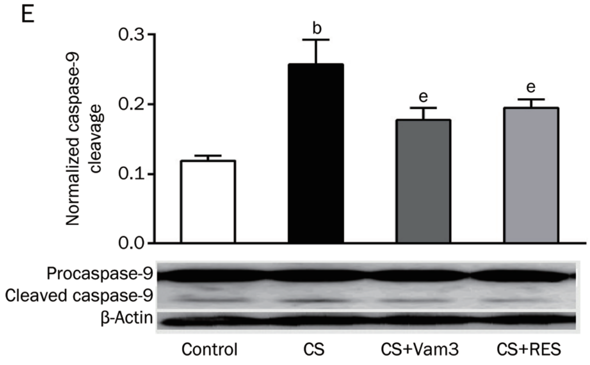

Figure 6. The effects of Vam3 on apoptosis in cigarette smoke (CS)-exposed mouse lungs. The mice were challenged with CS and pretreated with Vam3 or RES as described in the Methods. (A) A TUNEL assay was performed on the lungs from these animals. (B) shows the percentage of TUNEL-positive epithelial cells over the total cells in each visual field (n=4 per group). (C) The expression of Bax in the lung epithelial cells from each group was determined using immunohistochemical staining. (D) The graph represents the mean integrated optical density (IOD) of the Bax-positive cells (n=4 per group). (E) Procaspase-9 and cleaved caspase-9 (active form) expression was determined using Western blot analyses. The upper panel is the densitometrically quantitated cleaved caspase-9 expression based on the Western blot analysis (n=4 per group). $^{b}P<0.05$, $^{c}P<0.01$ *vs* the control. $^{e}P<0.05$, $^{f}P<0.01$ *vs* the CS challenge alone.

of caspase-9 were also examined. The expression of Bax was increased in the CS-exposed mouse lungs but was decreased in the lungs of mice treated with Vam3 or RES (Figure 6C and 6D). Vam3 and RES were also able to prevent the activation of caspase-9 (Figure 6E). Therefore, Vam3 inhibited apoptosis in CS-exposed mouse lungs *in vivo* through the mitochondrial pathway.

Vam3 attenuated ceramide accumulation

Ceramide exerts potent apoptotic effects in a variety of cell types. CSC increases intracellular levels of ceramide, which subsequently induces apoptosis[7]. The modulatory effects of Vam3 on CSC-induced ceramide accumulation were examined in BEAS-2B cells. We utilized the nSMase inhibitor GW4869 as a positive control. Treatment with CSC resulted in the increase of ceramide with the exception of C18:0 (Figure 7A). However, Vam3 pretreatment inhibited the accumulation of C18:0, C20:0, and C22:0 ceramide following the CSC treatment, similar to the effect of GW4869. Similar results were observed in MCF7 cells in which the pretreatment of the cells with Vam3 and GW4869 attenuated the TNF-α-induced ceramide accumulation (Figure 7B). To examine the efficacy of Vam3 on ceramide accumulation *in vivo*, BALB/c mice were exposed to CS with or without Vam3 (50 mg/kg) or RES (30 mg/kg) treatment. All ceramides accumulated in the lungs of the mice exposed to CS, while the pretreatment with Vam3 and not RES reduced the C16:0, C18:0, and C22:0 ceramide increases caused by CS (Figure 7C).

Vam3 pretreatment diminished nSMase2 up-regulation

Ceramides can be generated *de novo* from sphingosine or in the reverse pathway of sphingomyelin hydrolysis by sphin-

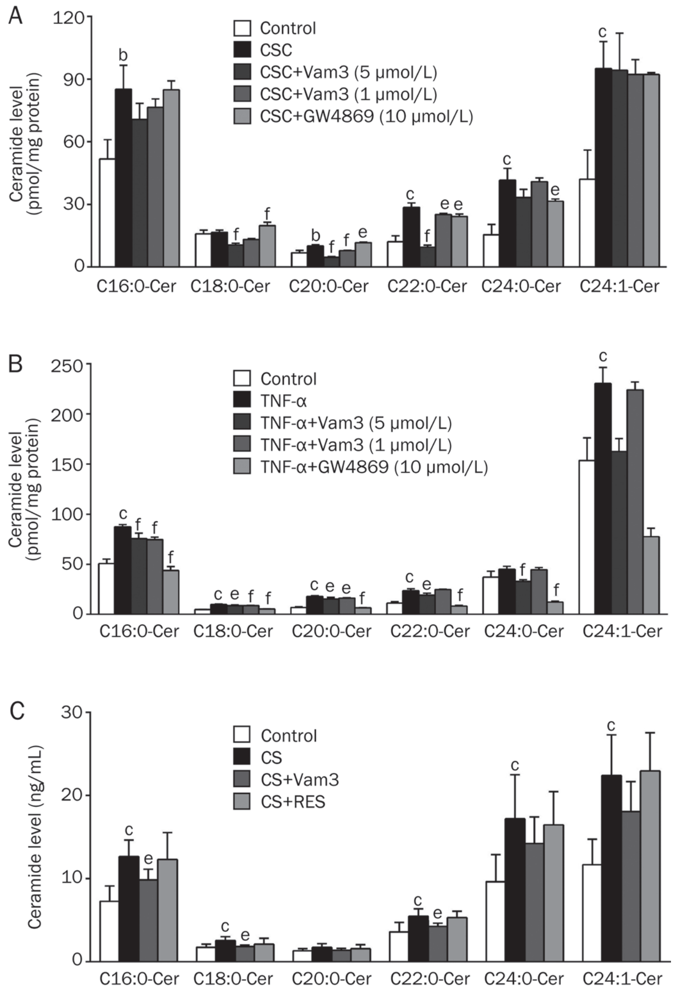

Figure 7. The effects of Vam3 on ceramide production in cigarette smoke condensate (CSC)-challenged BEAS-2B cells (A), TNF-α-challenged MCF7 cells (B) or cigarette smoke (CS)-exposed mouse lung tissues (C). (A) BEAS-2B cells were plated in 6-well plates and treated with 0, 5, or 1 μmol/L Vam3 or 10 μmol/L GW4869 for 2 h followed by challenge with CSC (300 mg/L) or the diluent as a control for 12 h. The cellular lipids were extracted and assayed for ceramide with different structures using HPLC-MS/MS (n=3 for each group). (B) Similar experimental design to Panel A using MCF7 cells but challenged with TNF-α (10 ng/mL), not CSC. (C) The mice were challenged with CS and pretreated with Vam3 or RES as described in Methods. Lung tissues from each group were used for the ceramide analysis using HPLC-MS/MS (n=4 for each group). $^{b}P<0.05$, $^{c}P<0.01$ *vs* the control. $^{e}P<0.05$, $^{f}P<0.01$ *vs* CSC, TNF-α, or CS challenge alone in Panels A, B, and C, respectively.

gomyelinase (SMase) isoenzymes. TNF-α-induced ceramide accumulation and subsequent cell death could be mediated by the activation of nSMase[24]. nSMase2 is involved in ceramide generation, apoptosis and lung injury after CS exposure[7]. nSMase2 mRNA and protein levels were examined by real-time qPCR and Western blotting in both *in vitro* and *in vivo* studies. The treatment of BEAS-2B cells with CSC (Figure 8A and 8B) or MCF7 cells with TNF-α (Figure 8C and 8D) significantly increased nSMase2 mRNA and protein levels ($P<0.05$ for both). While pretreatment with 5 and 1 μmol/L Vam3 revealed lower nSMase2 mRNA and protein levels ($P<0.05$ for all groups compared with the CSC or TNF-α challenge alone) but did not differ from the effect of GW4869 ($P<0.05$ for all compared to CSC or TNF-α challenge alone), a reagent that has been verified to inhibit nSMase2 expression.

Consistent with the *in vitro* studies above, in BALB/c mice, exposure to CS induced a significant increase in the nSMase2 mRNA and protein expression in the lungs ($P<0.05$ compared with the normal control group), whereas pretreatment with Vam3 attenuated this increase ($P<0.05$ compared with the CS challenge alone), the effect of which was greater than that of RES (P=NS compared with the CS challenge alone) (Figure 8E and 8F). Taken together, these data indicate that Vam3 pretreatment diminished the nSMase2 up-regulation caused by apoptotic stimuli.

Discussion

In our previous study, we demonstrated that Vam3 inhibited autophagy in CSC-treated BEAS-2B cells and CS-exposed mouse lungs[16]. Here, we report the protective effects of Vam3 on apoptosis in both *in vitro* and *in vivo* models. C_6-pyridinium ceramide, a mitochondrial-targeting ceramide, has been identified as a potent inducer of mitochondria-dependent apoptosis. We used C_6-pyridinium ceramide-induced apoptosis in MCF7 cells as a preliminary screening method to investigate the effects and mechanisms of Vam3 on apoptosis. Vam3 attenuated C_6-pyridinium ceramide-induced mitochondria-dependent apoptosis in MCF7 cells. We then further confirmed that Vam3 inhibited apoptosis in both CSC-treated BEAS-2B cells *in vitro* and CS-exposed mouse lungs *in vivo* and also attenuated CS-induced apoptosis *in vitro* and *in vivo* by improving mitochondrial function. Furthermore, exposure to CS led to ceramide generation and elevated apoptosis in lung epithelial cells, while Vam3 reduced ceramide production by down-regulating nSMase2 expression.

Mitochondria play a major role in apoptosis. Mitochondrial dysfunction in cell apoptosis is characterized by a decline in the mitochondrial membrane potential, increased production of ROS, respiratory impairment and the release of apoptogenic proteins, including cytochrome *c* and apoptosis-inducing factor (AIF)[21]. It has been reported that mitochondrial membrane potential loss plays a central role in the initiation of apoptosis and is linked to the release of cytochrome *c* from the mitochondria to the cytoplasm[25, 26]. In the present study, we found that Vam3 prevented the mitochondrial membrane potential loss, decreased ROS production and subsequently attenuated the release of cytochrome *c* induced by C_6-pyridinium ceramide. These findings indicated the protective effects of Vam3 on mitochondrial dysfunction. Cytochrome *c*, once released from the mitochondria, will interact with Apaf-1 and activated caspase-9, amplifying the apoptotic process[23]. Vam3 attenuated the C_6-pyridinium ceramide-induced caspase-9 activation, indicating that the anti-apoptotic effects of Vam3 may be linked to the caspase-9-regulated intrinsic pathway.

The Bcl-2 family is involved in the regulation of mito-

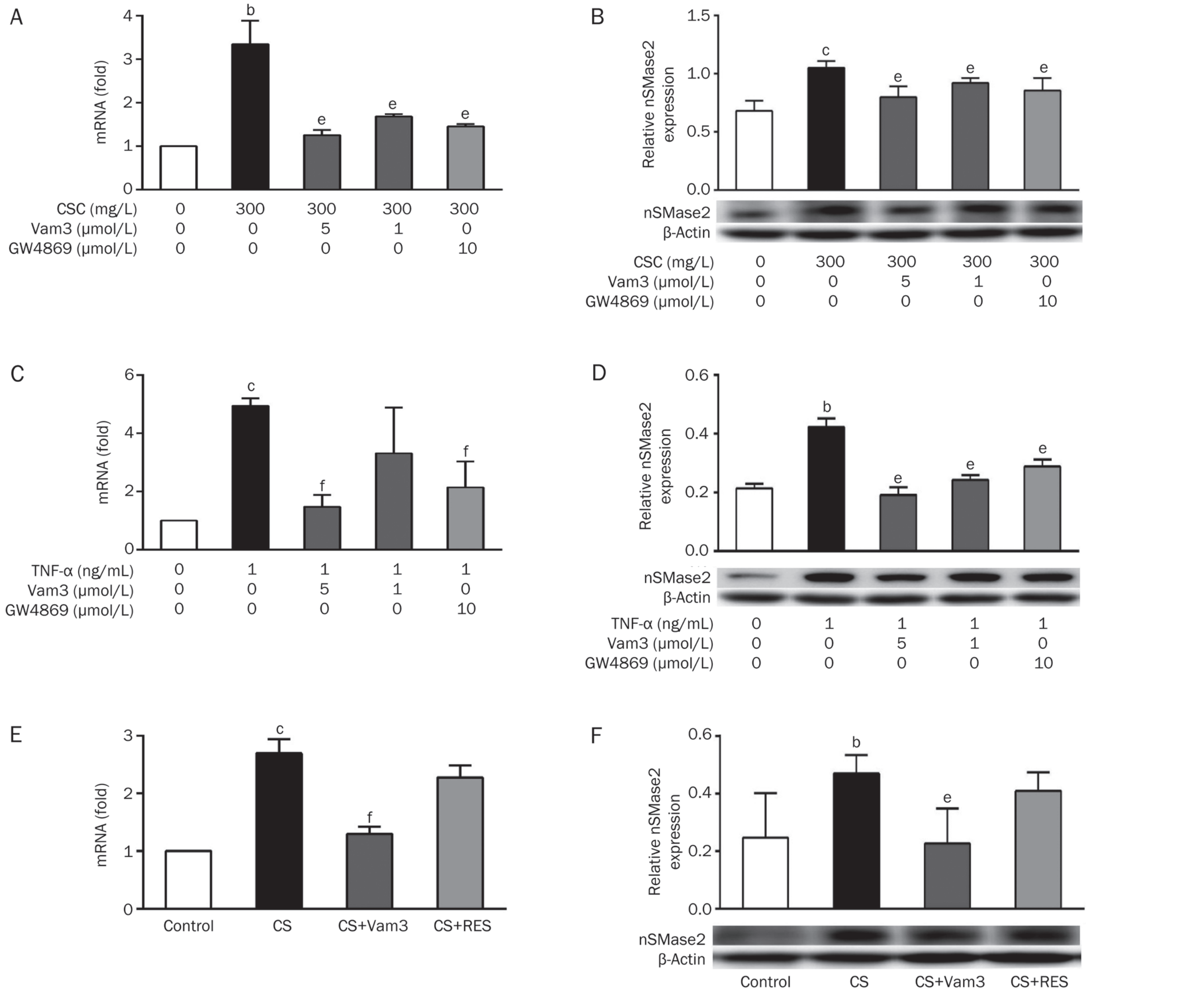

Figure 8. The effects of Vam3 on nSMase2 expression in cigarette smoke condensate (CSC)-challenged BEAS-2B cells (A and B), TNF-α-challenged MCF7 cells (C and D) or cigarette smoke (CS)-exposed mouse lung tissues (E and F). (A and B) BEAS-2B cells were plated in 6-well plates and treated with 0, 5, or 1 μmol/L Vam3 or 10 μmol/L GW4869 for 2 h followed by challenge with CSC (300 mg/L) or the diluent as a control for 6 h (A) or 12 h (B). In panel A, the mRNA level of nSMase2 was determined by real-time qPCR. (B) Western blot analysis was used to determine the nSMase2 and β-actin expression, and the densitometrically quantitated nSMase2 is shown in the upper panel (*n*=3 for each group). (C and D) Similar experimental design to Panels A and B using MCF7 cells but challenged with TNF-α (10 ng/mL), not CSC. (E and F) The mice were challenged with CS and pretreated with Vam3 or RES as described in the Methods. Lung tissues from each group were used for nSMase2 expression measurement using real-time qPCR (E) and Western blot analysis (Panel F) (*n*=4 for each group). $^{b}P<0.05$, $^{c}P<0.01$ *vs* the control. $^{e}P<0.05$, $^{f}P<0.01$ *vs* the CSC, TNF-α, or CS challenge alone in Panel A and B, C and D, E and F, respectively.

chondrial dependent apoptosis. Bcl-2 proteins are upstream regulators of mitochondrial membrane potential. The pro-apoptotic protein Bax is responsible for the formation of pores in the outer mitochondrial membrane[27, 28]. Bax, localized in the cytoplasm under normal conditions, translocates to the mitochondria in response to C_6-pyridinium ceramide[20]. The mitochondria-bound Bax is associated with the loss of mitochondrial membrane potential and the release of cytochrome *c* from the mitochondrial inter-membrane space[21]. The present study shows that Vam3 pretreatment significantly reduced C_6-pyridinium ceramide-induced Bax translocation to the mitochondria in a dose dependent manner. Therefore, Vam3 prevented C_6-pyridinium ceramide-induced mitochondrial dysfunction by regulating Bax redistribution.

Because Vam3 could attenuate C_6-pyridinium ceramide-induced mitochondria-dependent apoptosis in MCF7 cells, we next determined the efficacy of Vam3 as an inhibitor of apoptosis in CSC-treated BEAS-2B cells and in CS-exposed mouse lungs. As expected, in the BEAS-2B cells, the CSC challenge promoted apoptosis, mitochondrial membrane potential loss,

cytochrome *c* release, and caspase-9 activation, while the Vam3 pretreatment attenuated CSC-induced apoptosis through the mitochondrial pathway. Consistent with this *in vitro* study, pretreatment with Vam3 also reduced epithelial cell apoptosis in CS-exposed mouse lungs through the mitochondrial pathway.

The up-regulation of ceramide was reported in the lungs of COPD patients and CS exposed mice. Ceramide accumulation in the lungs was associated with the increased death rate of bronchial epithelial cells[2]. In CSC-treated BEAS-2B cells and CS-exposed mouse lungs, ceramide levels increased, in agreement with other reports[2, 7]; however, this intracellular ceramide accumulation could be attenuated by Vam3 treatment. Furthermore, we have also observed that Vam3 could counteract the ceramide increase induced by TNF-α in MCF7 cells.

Ceramide can be generated *de novo* from sphingosine or in the reverse pathway of sphingomyelin hydrolysis by sphingomyelinase (SMase) isoenzymes. Sphingomyelin hydrolysis by SMase is considered to be the major pathway of stress-induced ceramide production[29]. There are three isoforms of SMase distinguished by their pH optima: acid SMase (aSMase), neutral SMase (nSMase) and secretory SMase. TNF-α-induced ceramide accumulation and subsequent cell death were mediated by the activation of nSMase[30]. Levy *et al* reported that in human airway epithelial cells, it is nSMase2 that is activated by CS or H_2O_2, and this activation leads to the increase in ceramide levels and epithelial cell apoptosis[7]. nSMase2 has also been shown to be responsible for ceramide generation, apoptosis and lung injury after CS exposure. Exposure to CS leads to ceramide generation and excessive apoptosis induction in airway epithelial cells through the specific activation of nSMase2[2]. Therefore, we hypothesized that a Vam3-mediated reduction in ceramide generation may be associated with the down-regulation of nSMase2 expression. Our results showed that nSMase2 expression increased in BEAS-2B cells and mouse lungs exposed to CS and that this increase could be counteracted by Vam3. Similar results were also observed in MCF7 cells treated with TNF-α. It can be speculated that Vam3 may regulate ceramide production by targeting nSMase2 or its upstream mediators.

In conclusion, Vam3 has protective effects against C_6-pyridinium ceramide and CS induced apoptosis through the suppression of C_6-pyridinium ceramide-induced Bax redistribution, ROS generation, mitochondrial membrane potential loss, cytochrome *c* release, and the subsequent inhibition of caspase activation and counteracting ceramide production through inhibiting nSMase2 overexpression. Given that Vam3 has an anti-oxidative effect, and nSMase2 up-regulation and ceramide-induced apoptosis are both related to oxidative stress, we propose that Vam3 might exert its effects through its anti-oxidative properties.

Acknowledgements
This work was supported by grants from the National Twelfth-Five Year Research Program of China (№ 37007 and 37008).

Author contribution
Ling-ling XUAN designed and performed the experiments and wrote the paper; Ji SHI provided assistance in the experiment design; Chun-suo YAO contributed reagents; Jin-ye BAI, Feng QU, and Jin-lan ZHANG assisted with the studies; Qi HOU designed the research, analyzed the data and revised the paper.

References
1 Shen YC, Yang T, Guo SJ, Li XO, Chen L, Wang T, *et al*. Increased serum ox-LDL levels correlated with lung function, inflammation, and oxidative stress in COPD. Mediators Inflamm 2013; 2013: 972347.
2 Filosto S, Castillo S, Danielson A, Franzi L, Khan E, Kenyon N, *et al*. Neutral sphingomyelinase 2: a novel target in cigarette smoke-induced apoptosis and lung injury. Am J Respir Cell Mol Biol 2011; 44: 350–60.
3 Vijayan VK. Chronic obstructive pulmonary disease. Indian J Med Res 2013; 137: 251–69.
4 Mouded M, Egea EE, Brown MJ, Hanlon SM, Houghton AM, Tsai LW, *et al*. Epithelial cell apoptosis causes acute lung injury masquerading as emphysema. Am J Respir Cell Mol Biol 2009; 41: 407–14.
5 Comer DM, Kidney JC, Ennis M, Elborn JS. Airway epithelial cell apoptosis and inflammation in COPD, smokers and nonsmokers. Eur Respir J 2013; 41: 1058–67.
6 Petrache I, Petrusca DN, Bowler RP, Kamocki K. Involvement of ceramide in cell death responses in the pulmonary circulation. Proc Am Thorac Soc 2011; 8: 492–6.
7 Levy M, Khan E, Careaga M, Goldkorn T. Neutral sphingomyelinase 2 is activated by cigarette smoke to augment ceramide-induced apoptosis in lung cell death. Am J Physiol Lung Cell Mol Physiol 2009; 297: 125–33.
8 Andrieu-Abadie N, Gouazé V, Salvayre R, Levade T. Ceramide in apoptosis signaling: relationship with oxidative stress. Free Radic Biol Med 2001; 31: 717–28.
9 Rouba HS, Maria OE, Hadile K, Ghassan D. p53 and ceramide as collaborators in the stress response. Int J Mol Sci 2013; 14: 4982–5012.
10 Siskind LJ, Kolesnick RN, Colombini M. Ceramide forms channels in mitochondrial outer membranes at physiologically relevant concentrations. Mitochondrion 2006; 6: 118–25.
11 Parra V, Moraga F, Kuzmicic J, López-Crisosto C, Troncoso R, Torrealba N, *et al*. Calcium and mitochondrial metabolism in ceramide-induced cardiomyocyte death. Biochim Biophys Acta 2013; 1832: 1334–44.
12 Ravid T, Tsaba A, Gee P, Rasooly R, Medina EA, Goldkorn T. Ceramide accumulation precedes caspase-3 activation during apoptosis of A549 human lung adenocarcinoma cells. Am J Physiol Lung Cell Mol Physiol 2003; 284: L1082–92.
13 Wojewodka G, De Sanctis JB, Radzioch D. Ceramide in cystic fibrosis: a potential new target for therapeutic intervention. J Lipids 2011; 2011: 674968.
14 Li YT, Yao CS, Bai JY, Lin M, Cheng GF. Anti-inflammatory effect of amurensin H on asthma-like reaction induced by allergen in sensitized mice. Acta Pharmacol Sin 2006; 27: 735–40.
15 Yang L, Yao CS, Wu ZY, Xuan LL, Bai JY, Cheng GF, *et al*. Effects of dihydroxy-stilbene compound Vam3 on airway inflammation, expression of ICAM-1, activities of NF-kappaB and MMP-9 in asthmatic mice. Yao Xue Xue Bao 2010; 45: 1503–8.
16 Shi J, Yin N, Xuan LL, Yao CS, Meng AM, Hou Q. Vam3, a derivative

of resveratrol, attenuates cigarette smoke-induced autophagy. Acta Pharmacol Sin 2012; 33: 888–96.
17 Hou Q, Hsu YT. Bax translocates from cytosol to mitochondria in cardiac cells during apoptosis: development of a GFP-Bax-stable H9c2 cell line for apoptosis analysis. Am J Physiol Heart Circ Physiol 2005; 289: H477–87.
18 Chazotte B. Labeling mitochondria with TMRM or TMRE. Cold Spring Harb Protoc 2011; 2011: 895–7.
19 Qu F, Wu CS, Hou JF, Jin Y, Zhang JL. Sphingolipids as new biomarkers for assessment of delayed-type hypersensitivity and response to triptolide. PLoS ONE 2012; 7: e52454.
20 Hou Q, Jin J, Zhou H, Novgorodov SA, Bielawska A, Szulc ZM. Mitochondrially targeted ceramides preferentially promote autophagy, retard cell growth, and induce apoptosis. J Lipid Res 2011; 52: 278–88.
21 Bossy-Wetzel E, Barsoum MJ, Godzik A, Schwarzenbacher R, Lipton SA. Mitochondrial fission in apoptosis, neurodegeneration and aging. Curr Opin Cell Biol 2003; 15: 706–16.
22 Petrosillo G, Ruggiero FM, Paradies G. Role of reactive oxygen species and cardiolipin in the release of cytochrome *c* from mitochondria. FASEB J 2003; 17: 2202–8.
23 Kumarswamy R, Seth RK, Dwarakanath BS, Chandna S. Mitochondrial regulation of insect cell apoptosis: evidence for permeability transition pore-independent cytochrome-*c* release in the Lepidopteran Sf9 cells. Int J Biochem Cell Biol 2009; 41: 1430–40.
24 Dbaibo GS, El-Assaad W, Krikorian A, Liu B, Diab K, Idriss NZ, *et al*. Ceramide generation by two distinct pathways in tumor necrosis factor alpha-induced cell death. FEBS Lett 2001; 503: 7–12.
25 Yi CH, Vakifahmetoglu-Norberg H, Yuan J. Integration of apoptosis and metabolism. Cold Spring Harb Symp Quant Biol 2011; 76: 375–87.
26 Ly JD, Grubb DR, Lawen A. The mitochondrial membrane potential ($\Delta\psi$m) in apoptosis; an update. Apoptosis 2003; 8: 115–28.
27 Pangare M, Makino A. Mitochondrial function in vascular endothelial cell in diabetes. J Smooth Muscle Res 2012; 48: 1–26.
28 Krishnamoorthy G, Selvakumar K, Venkataraman P, Elumalai P, Arunakaran J. Lycopene supplementation prevents reactive oxygen species mediated reactive oxygen species mediated apoptosis in Sertoli cells of adult albino rats exposed to polychlorinated biphenyls. Interdiscip Toxicol 2013; 6: 83–92.
29 Ahn KH, Kim SK, Choi JM, Jung SY, Won JH, Back MJ, *et al*. Identification of heat shock protein 60 as a regulator of neutral sphingomyelinase 2 and its role in dopamine uptake. PLoS One 2013; 8: e67216.
30 Marchesini N, Luberto C, Hannun YA. Biochemical properties of mammalian neutral sphingomyelinase 2 and its role in sphingolipid metabolism. J Biol Chem 2003; 278: 13775–83.
