# Supplementary figures and images for: Correction: Resveratrol reduces the apoptosis induced by cigarette smoke extract by upregulating MFN2
Source: PLoS One. 2019 Mar 12;14(3):e0213877. doi: 10.1371/journal.pone.0213877 (PMC6413909; doi:10.1371/journal.pone.0213877)

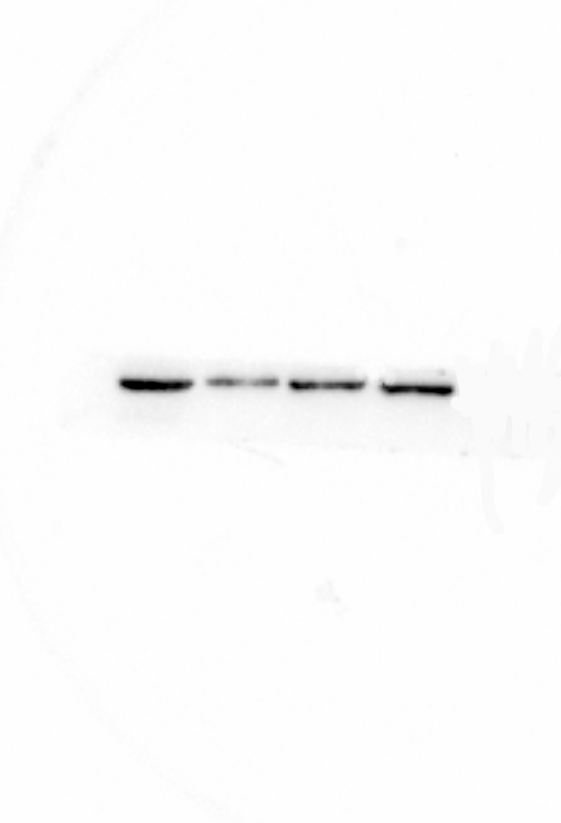

Supplement: S1 File — Raw data for mitochondria bax, Cytc and cytoplasm bax and Cytc. (ZIP) [file pone.0213877.s001.zip › 原始图/Cyto-bax1.tif]

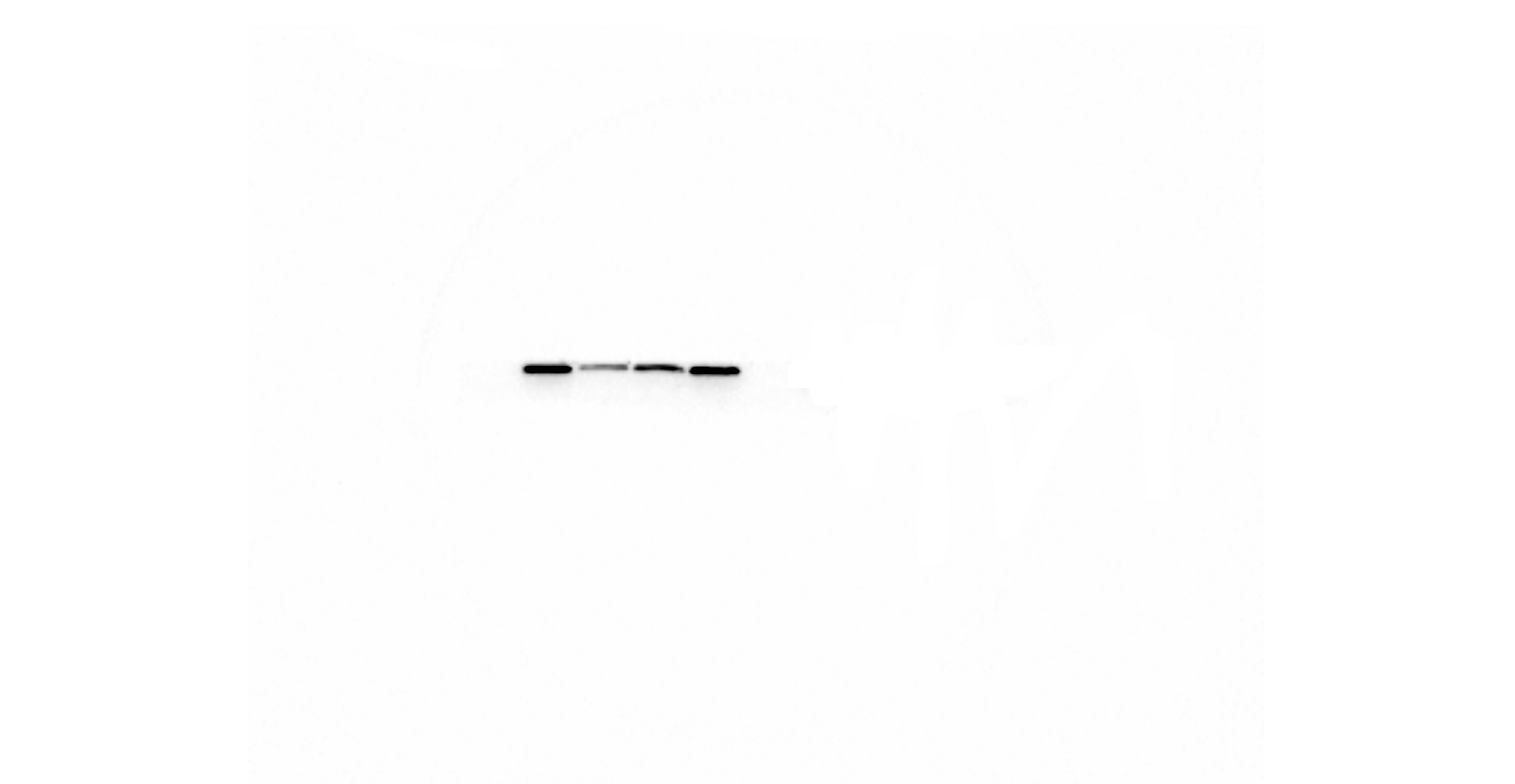

Supplement: S1 File — Raw data for mitochondria bax, Cytc and cytoplasm bax and Cytc. (ZIP) [file pone.0213877.s001.zip › 原始图/cyto-bax2.tif]

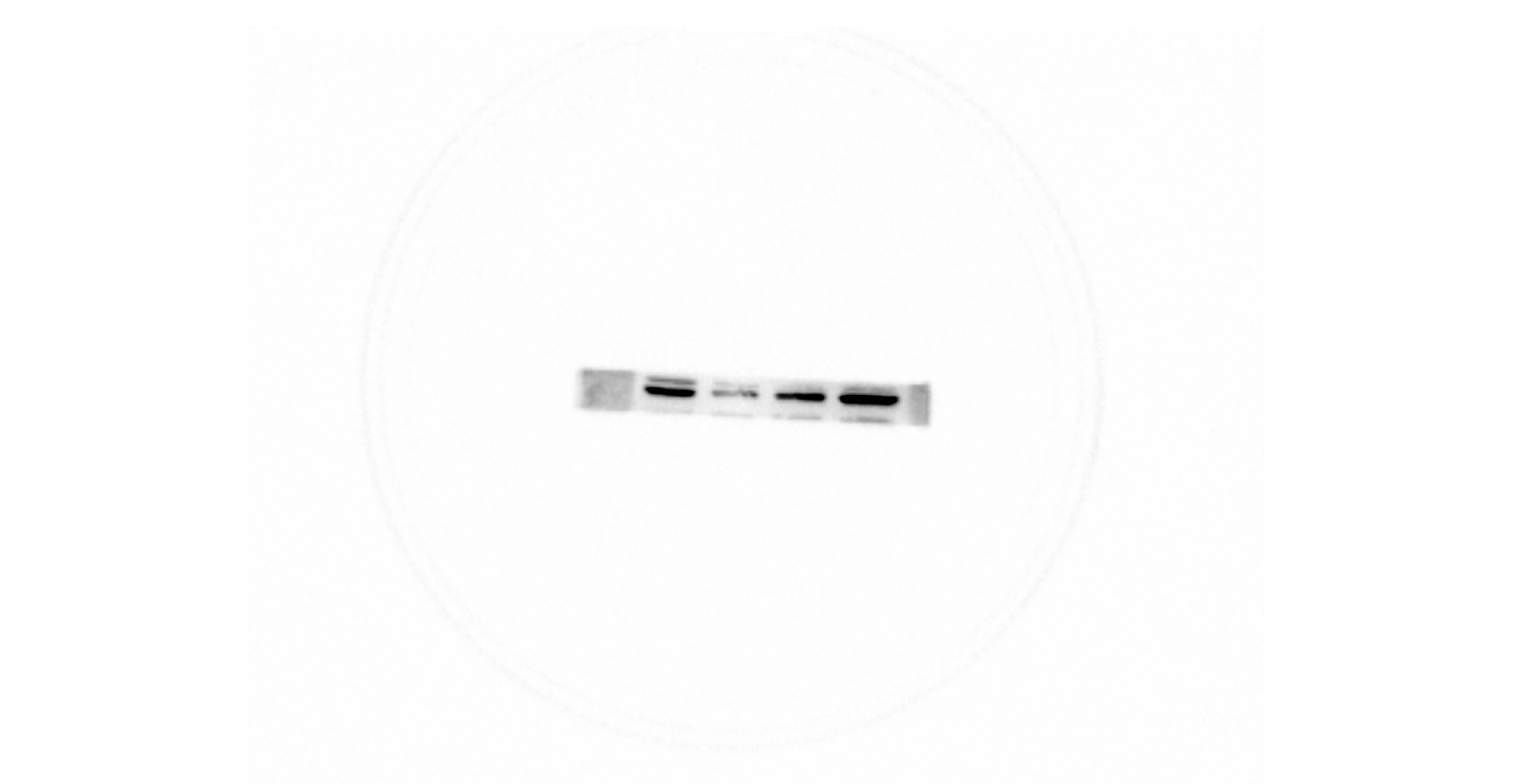

Supplement: S1 File — Raw data for mitochondria bax, Cytc and cytoplasm bax and Cytc. (ZIP) [file pone.0213877.s001.zip › 原始图/cyto-bax3.tif]

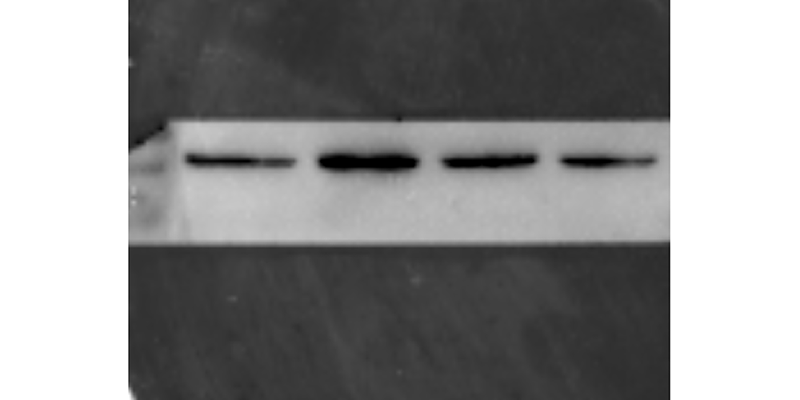

Supplement: S1 File — Raw data for mitochondria bax, Cytc and cytoplasm bax and Cytc. (ZIP) [file pone.0213877.s001.zip › 原始图/Cyto-cytc-1.tif]

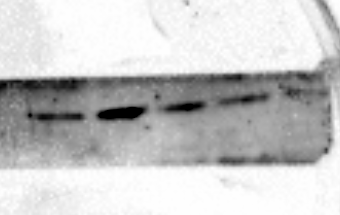

Supplement: S1 File — Raw data for mitochondria bax, Cytc and cytoplasm bax and Cytc. (ZIP) [file pone.0213877.s001.zip › 原始图/Cyto-cytc-2.tif]

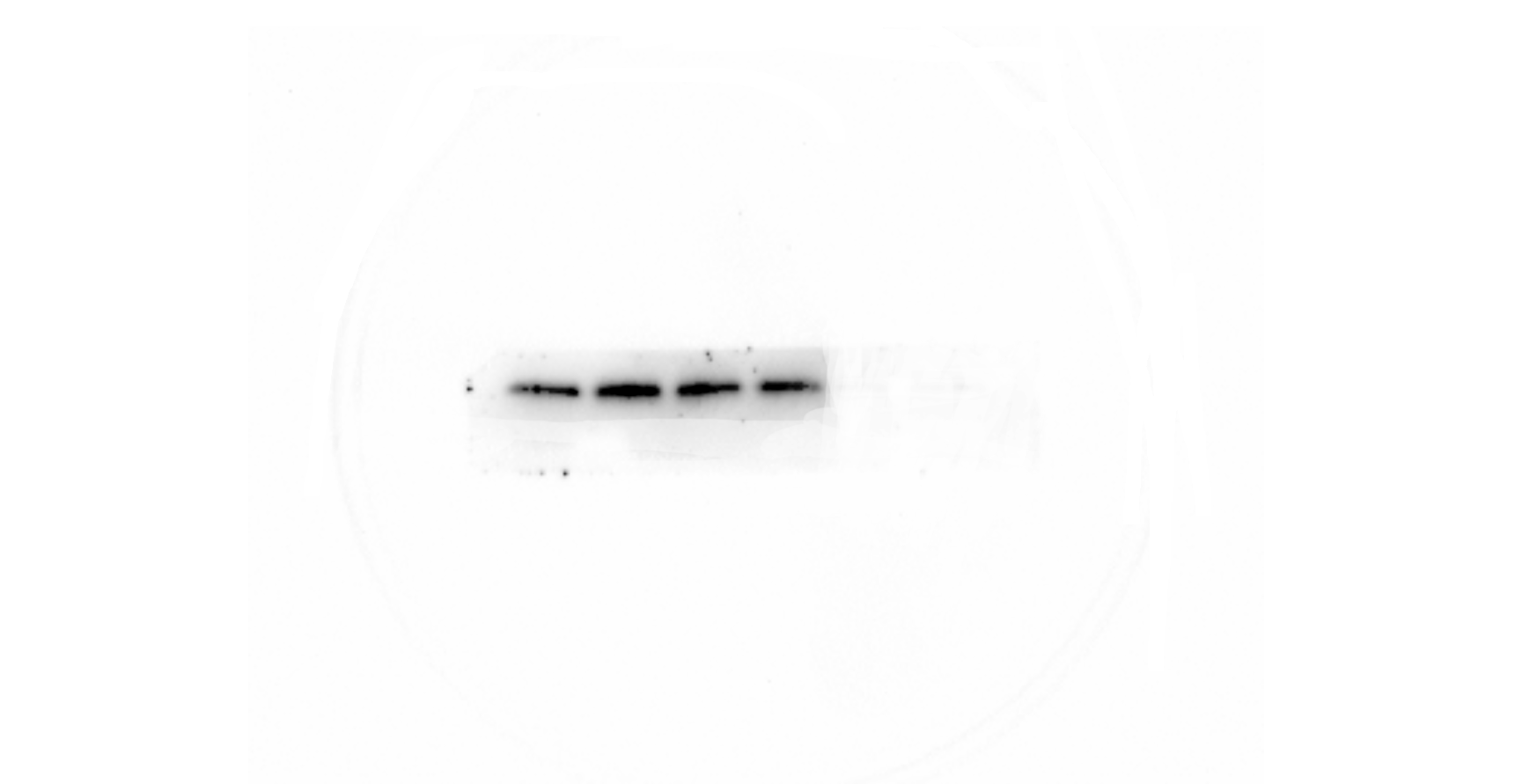

Supplement: S1 File — Raw data for mitochondria bax, Cytc and cytoplasm bax and Cytc. (ZIP) [file pone.0213877.s001.zip › 原始图/cyto-cytc-3.tif]

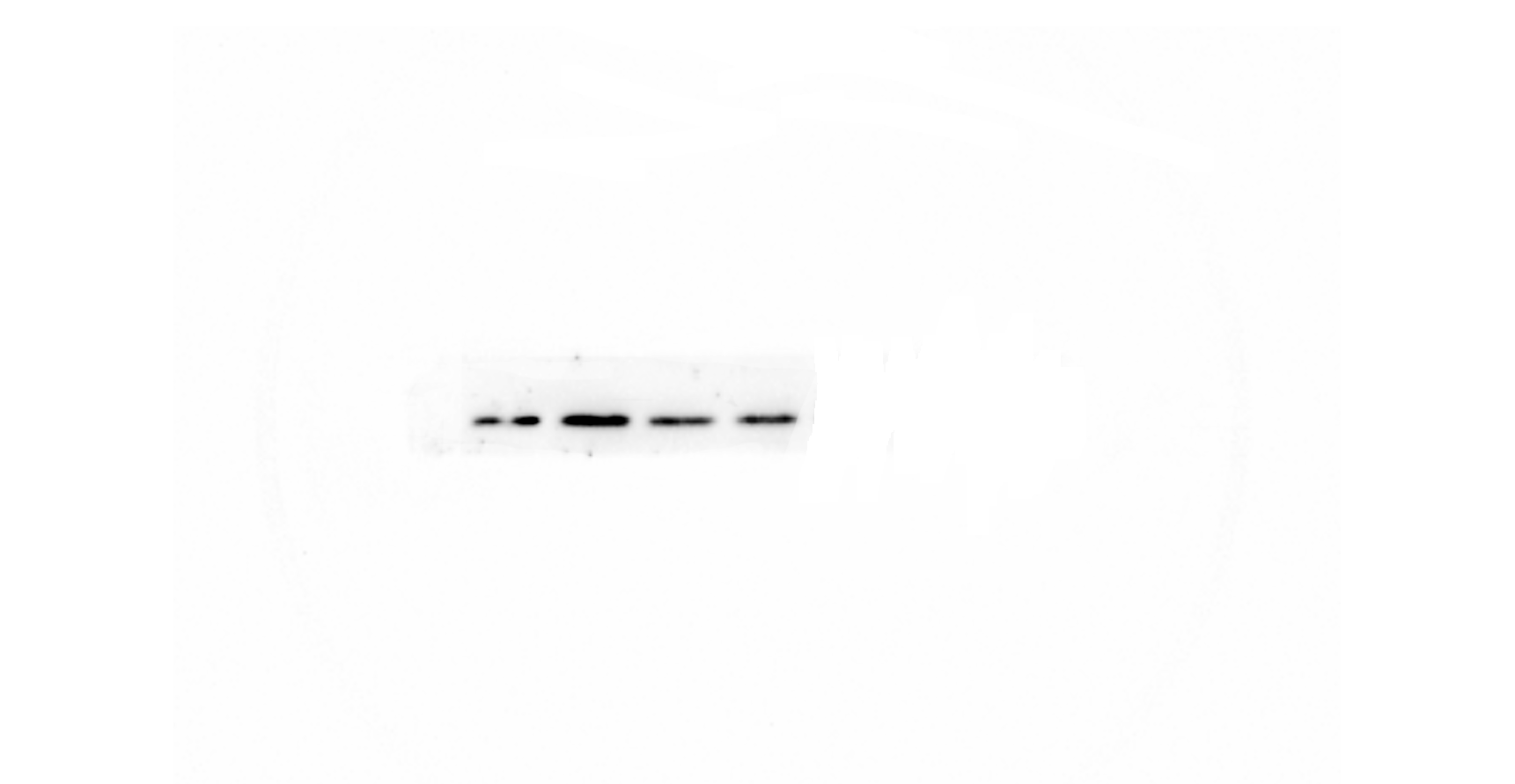

Supplement: S1 File — Raw data for mitochondria bax, Cytc and cytoplasm bax and Cytc. (ZIP) [file pone.0213877.s001.zip › 原始图/MITO-BAX1.tif]

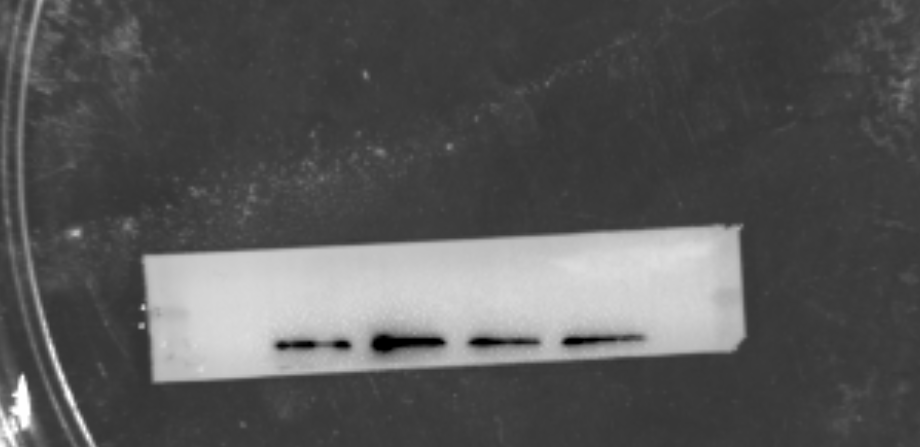

Supplement: S1 File — Raw data for mitochondria bax, Cytc and cytoplasm bax and Cytc. (ZIP) [file pone.0213877.s001.zip › 原始图/MITO-BAX2.tif]

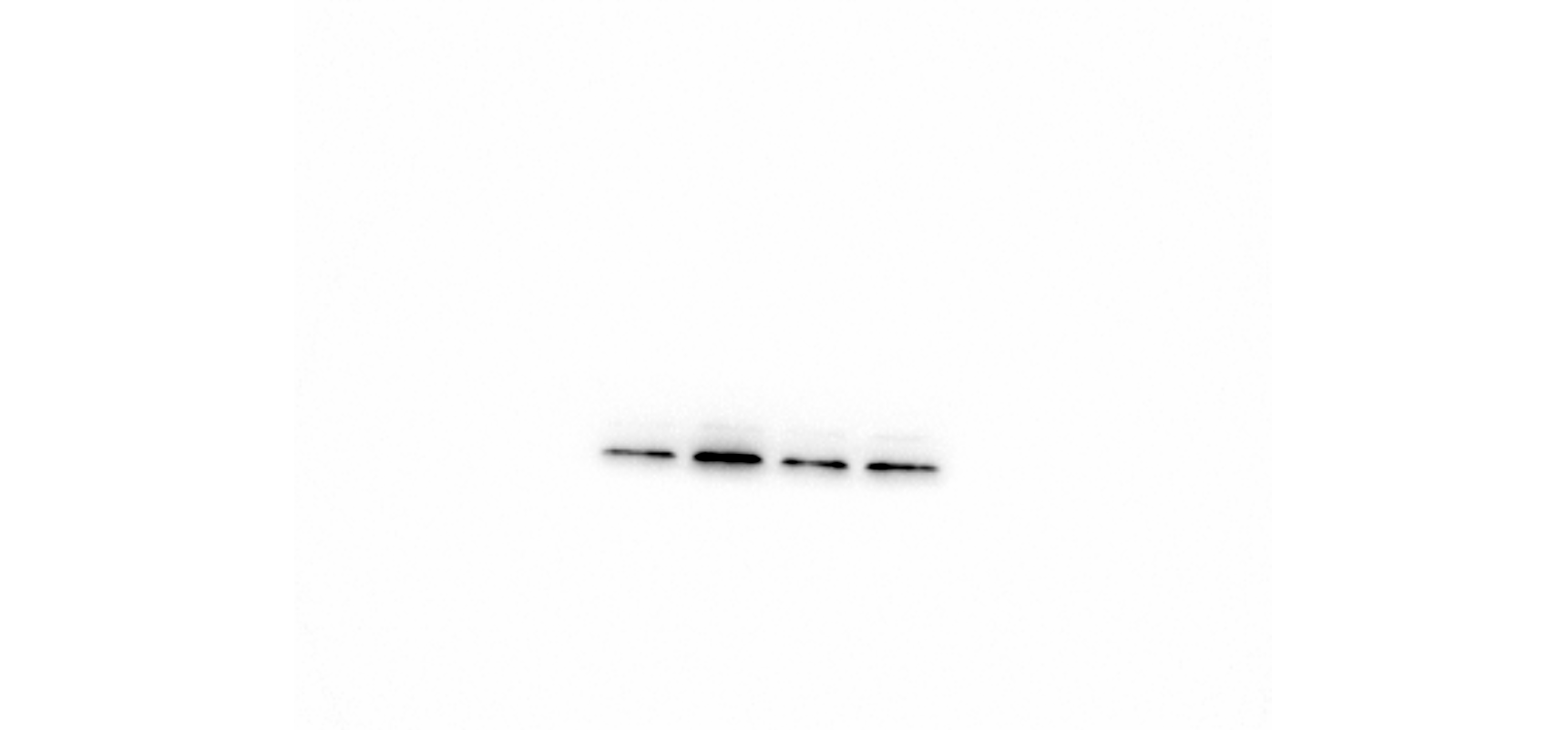

Supplement: S1 File — Raw data for mitochondria bax, Cytc and cytoplasm bax and Cytc. (ZIP) [file pone.0213877.s001.zip › 原始图/MITO-BAX3.tif]

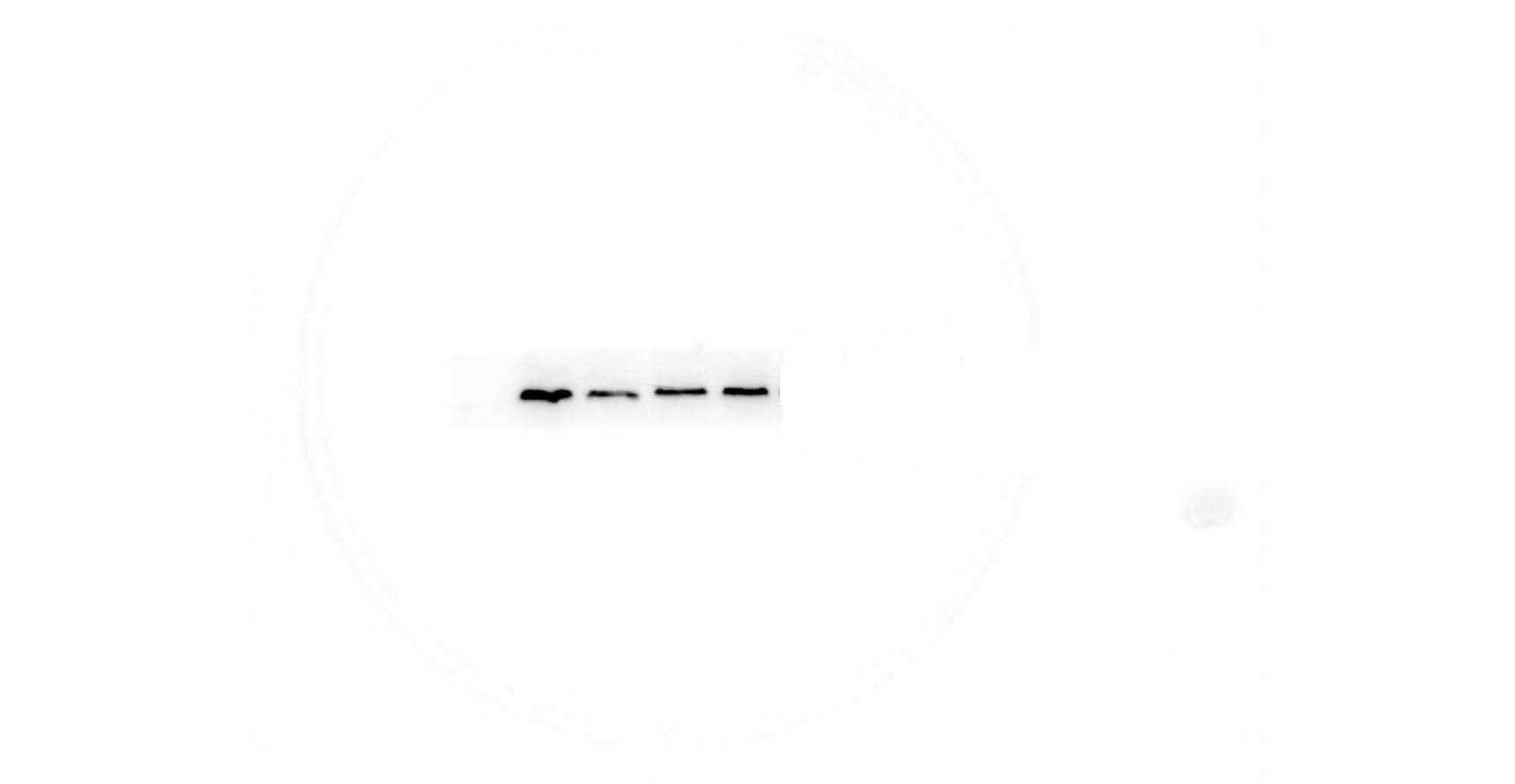

Supplement: S1 File — Raw data for mitochondria bax, Cytc and cytoplasm bax and Cytc. (ZIP) [file pone.0213877.s001.zip › 原始图/mito-cytc-1.tif]

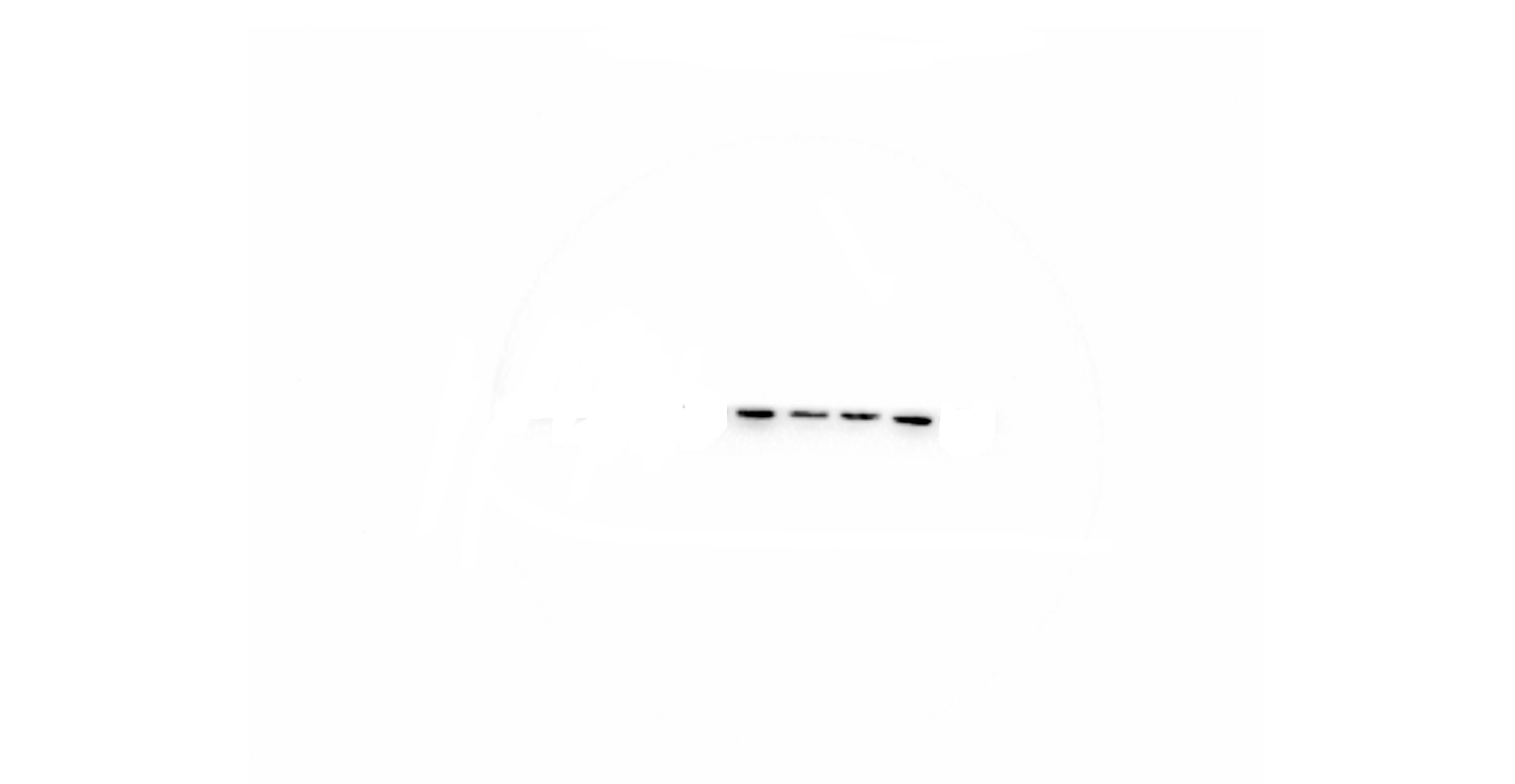

Supplement: S1 File — Raw data for mitochondria bax, Cytc and cytoplasm bax and Cytc. (ZIP) [file pone.0213877.s001.zip › 原始图/mito-cytc-2.tif]

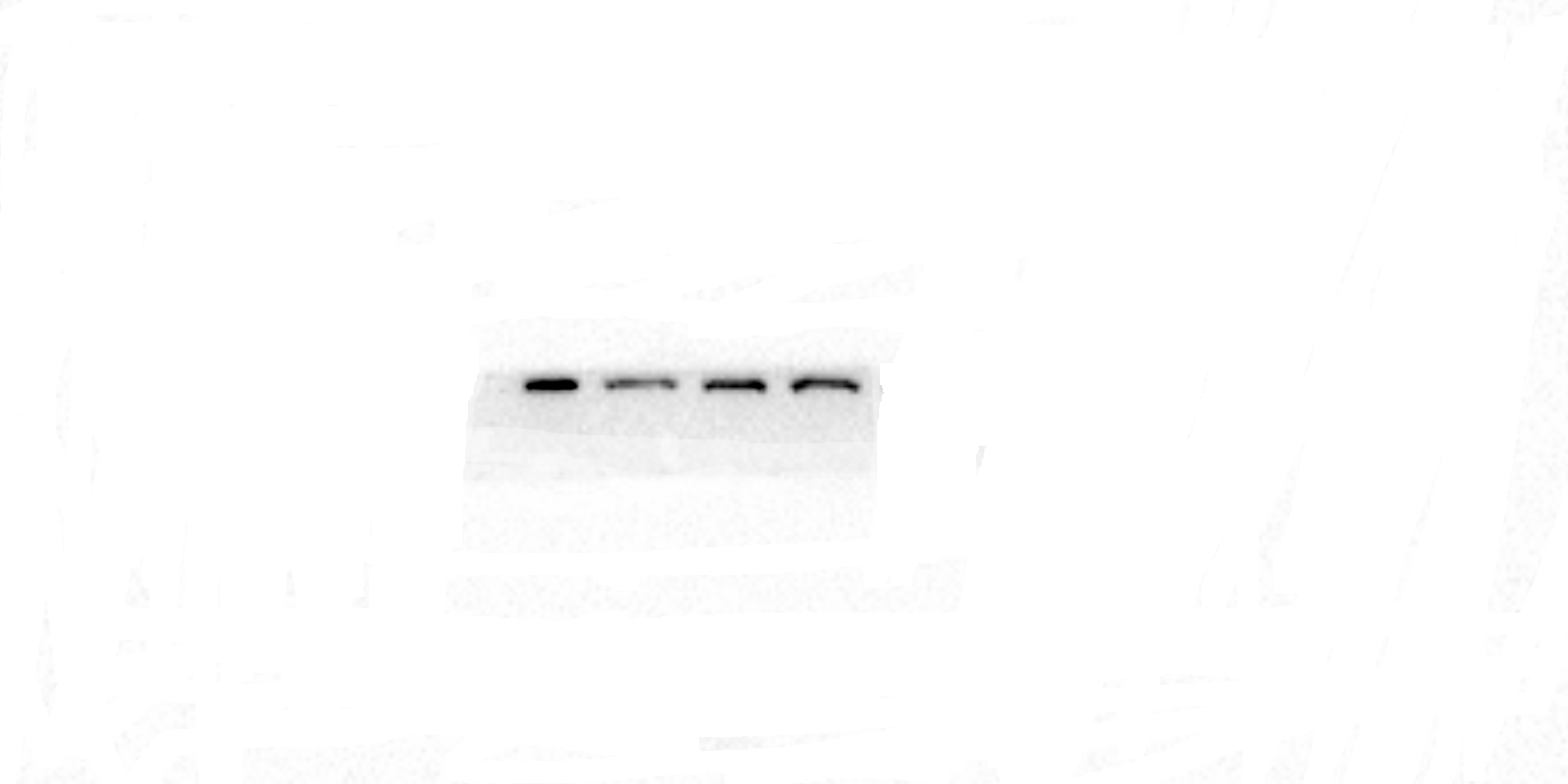

Supplement: S1 File — Raw data for mitochondria bax, Cytc and cytoplasm bax and Cytc. (ZIP) [file pone.0213877.s001.zip › 原始图/mito-cytc-3.tif]

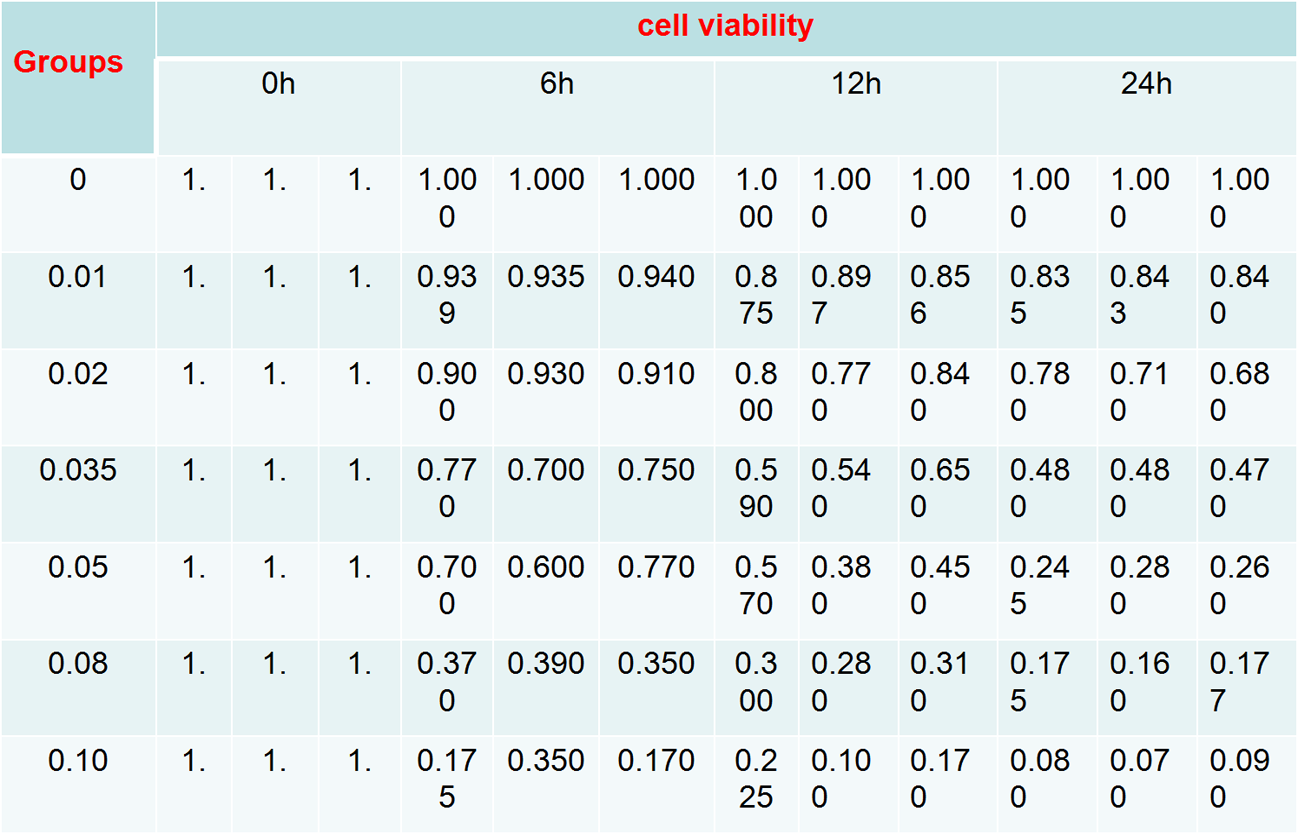

Supplement: S2 File — Raw data for Fig 1B, Fig 2A and 2B, Fig 3A, 3B, 3C and 3D, Fig 4B–4F, Fig 5A and 5B. (ZIP) [file pone.0213877.s002.zip › s1 Fig1B.tif]

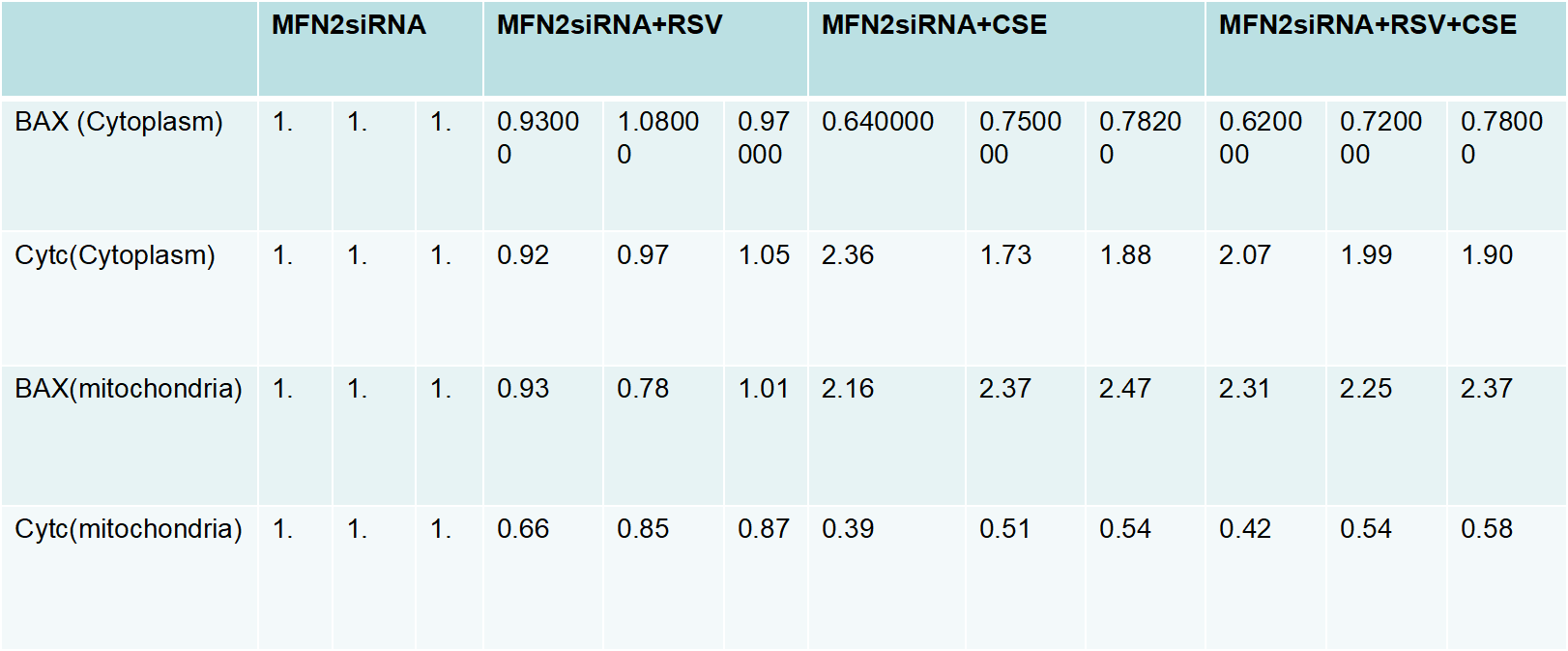

Supplement: S2 File — Raw data for Fig 1B, Fig 2A and 2B, Fig 3A, 3B, 3C and 3D, Fig 4B–4F, Fig 5A and 5B. (ZIP) [file pone.0213877.s002.zip › s10 Fig4E.tif]

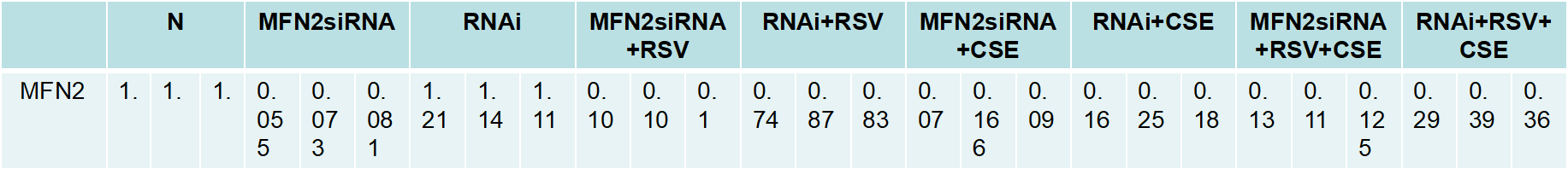

Supplement: S2 File — Raw data for Fig 1B, Fig 2A and 2B, Fig 3A, 3B, 3C and 3D, Fig 4B–4F, Fig 5A and 5B. (ZIP) [file pone.0213877.s002.zip › s11 Fig4F.tif]

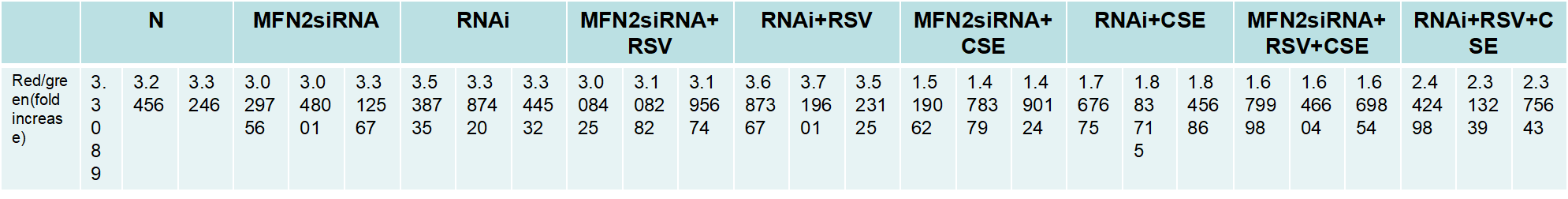

Supplement: S2 File — Raw data for Fig 1B, Fig 2A and 2B, Fig 3A, 3B, 3C and 3D, Fig 4B–4F, Fig 5A and 5B. (ZIP) [file pone.0213877.s002.zip › s12 Fig5A.tif]

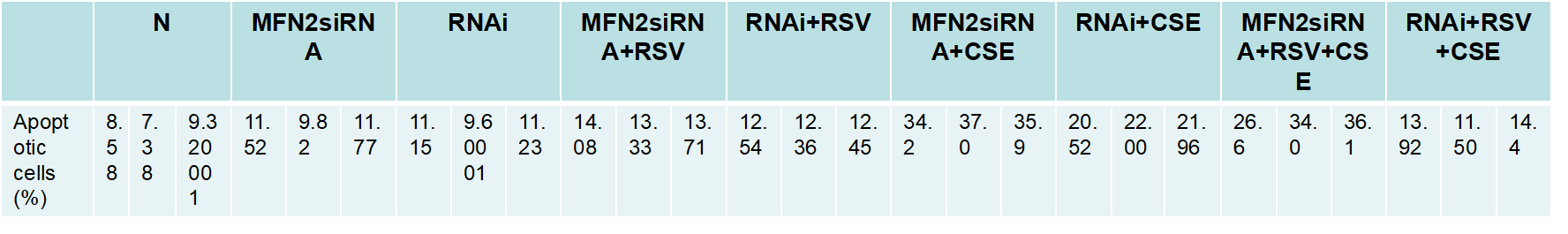

Supplement: S2 File — Raw data for Fig 1B, Fig 2A and 2B, Fig 3A, 3B, 3C and 3D, Fig 4B–4F, Fig 5A and 5B. (ZIP) [file pone.0213877.s002.zip › s13 Fig5B.tif]

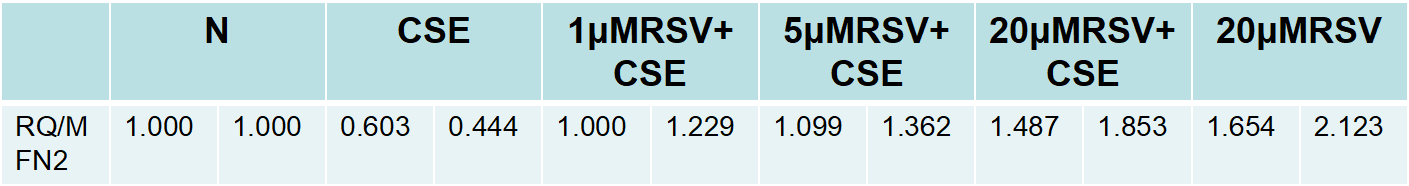

Supplement: S2 File — Raw data for Fig 1B, Fig 2A and 2B, Fig 3A, 3B, 3C and 3D, Fig 4B–4F, Fig 5A and 5B. (ZIP) [file pone.0213877.s002.zip › s14 Fig2B.tif]

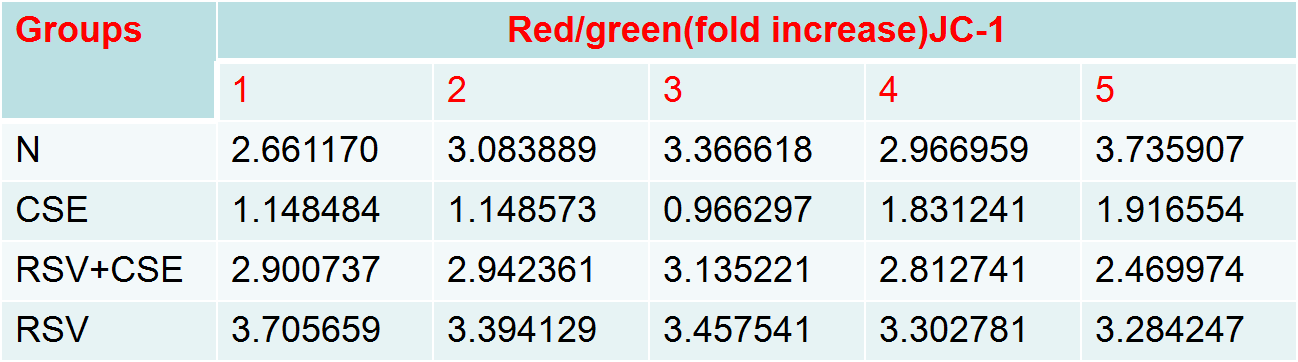

Supplement: S2 File — Raw data for Fig 1B, Fig 2A and 2B, Fig 3A, 3B, 3C and 3D, Fig 4B–4F, Fig 5A and 5B. (ZIP) [file pone.0213877.s002.zip › s2 Fig3C.tif]

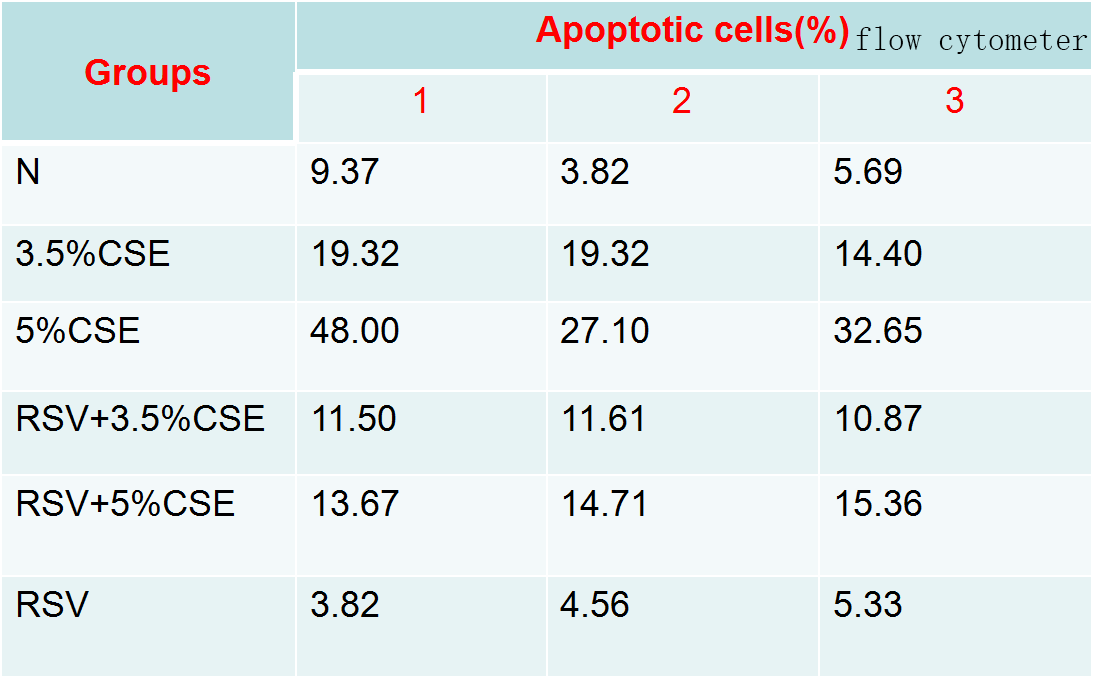

Supplement: S2 File — Raw data for Fig 1B, Fig 2A and 2B, Fig 3A, 3B, 3C and 3D, Fig 4B–4F, Fig 5A and 5B. (ZIP) [file pone.0213877.s002.zip › s3 Fig3D.tif]

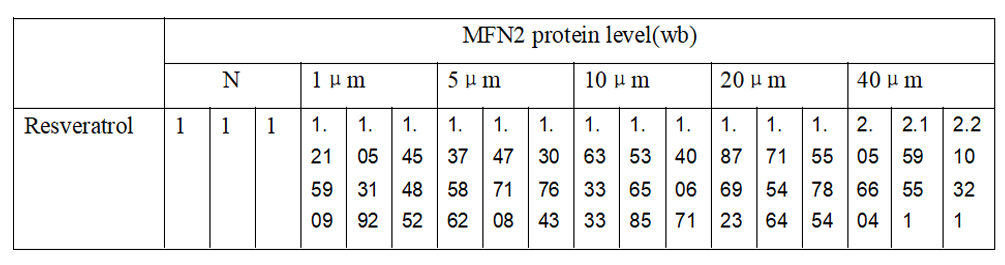

Supplement: S2 File — Raw data for Fig 1B, Fig 2A and 2B, Fig 3A, 3B, 3C and 3D, Fig 4B–4F, Fig 5A and 5B. (ZIP) [file pone.0213877.s002.zip › s4 Fig2A.tif]

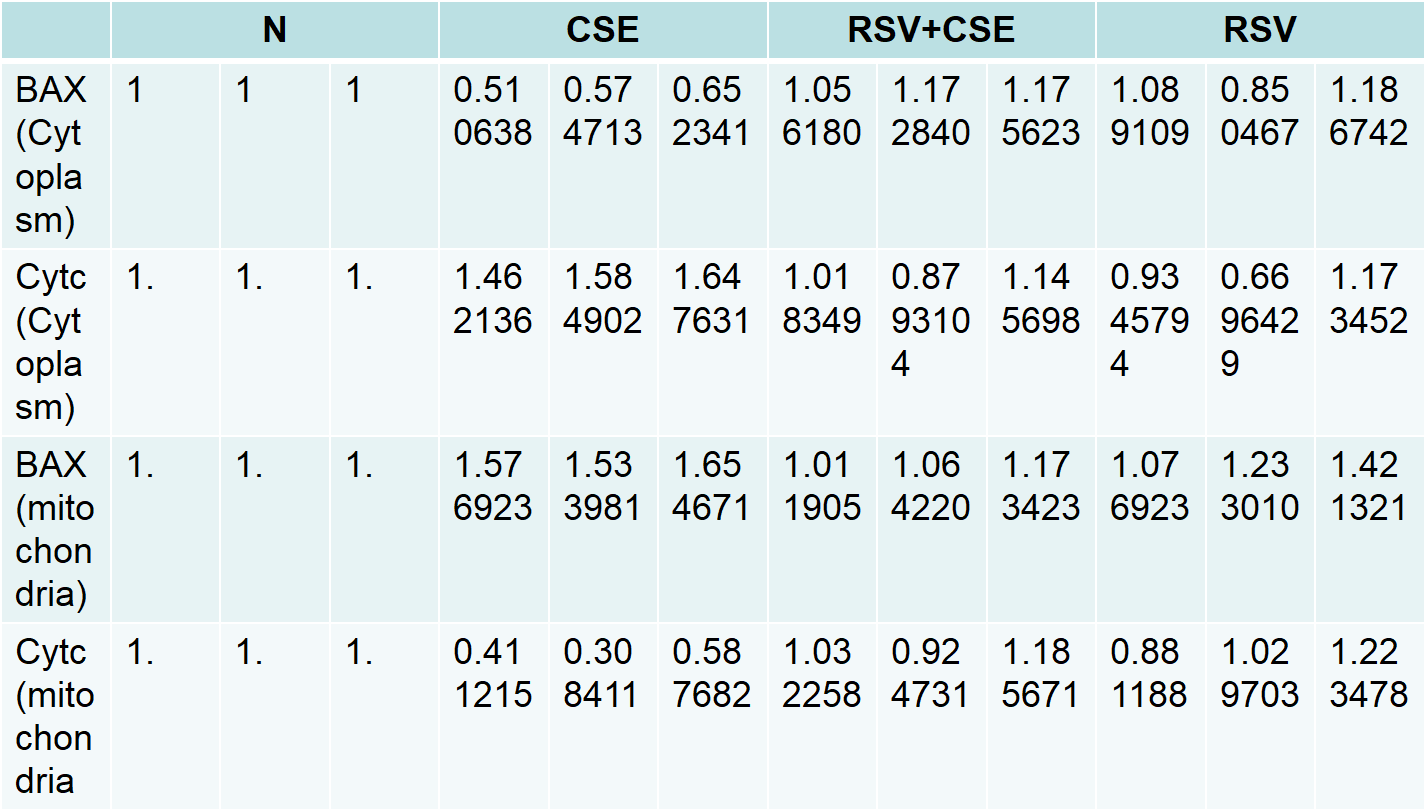

Supplement: S2 File — Raw data for Fig 1B, Fig 2A and 2B, Fig 3A, 3B, 3C and 3D, Fig 4B–4F, Fig 5A and 5B. (ZIP) [file pone.0213877.s002.zip › s5 Fig3A.tif]

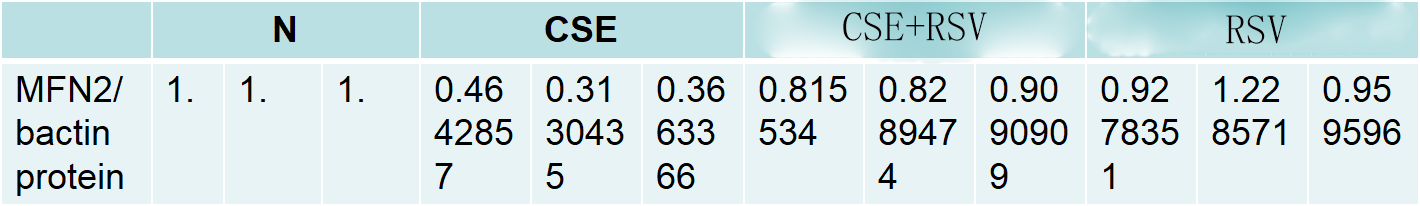

Supplement: S2 File — Raw data for Fig 1B, Fig 2A and 2B, Fig 3A, 3B, 3C and 3D, Fig 4B–4F, Fig 5A and 5B. (ZIP) [file pone.0213877.s002.zip › s6 Fig3B.tif]

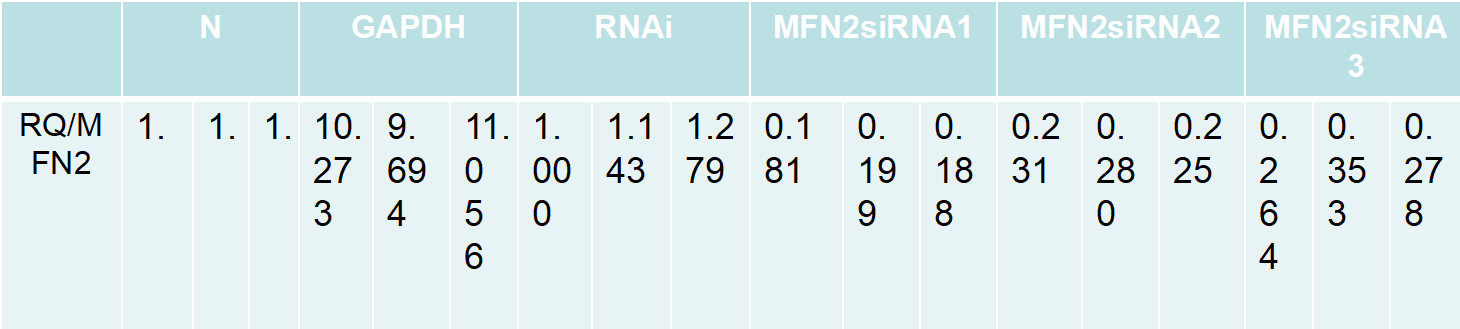

Supplement: S2 File — Raw data for Fig 1B, Fig 2A and 2B, Fig 3A, 3B, 3C and 3D, Fig 4B–4F, Fig 5A and 5B. (ZIP) [file pone.0213877.s002.zip › S7 Fig4B.tif]

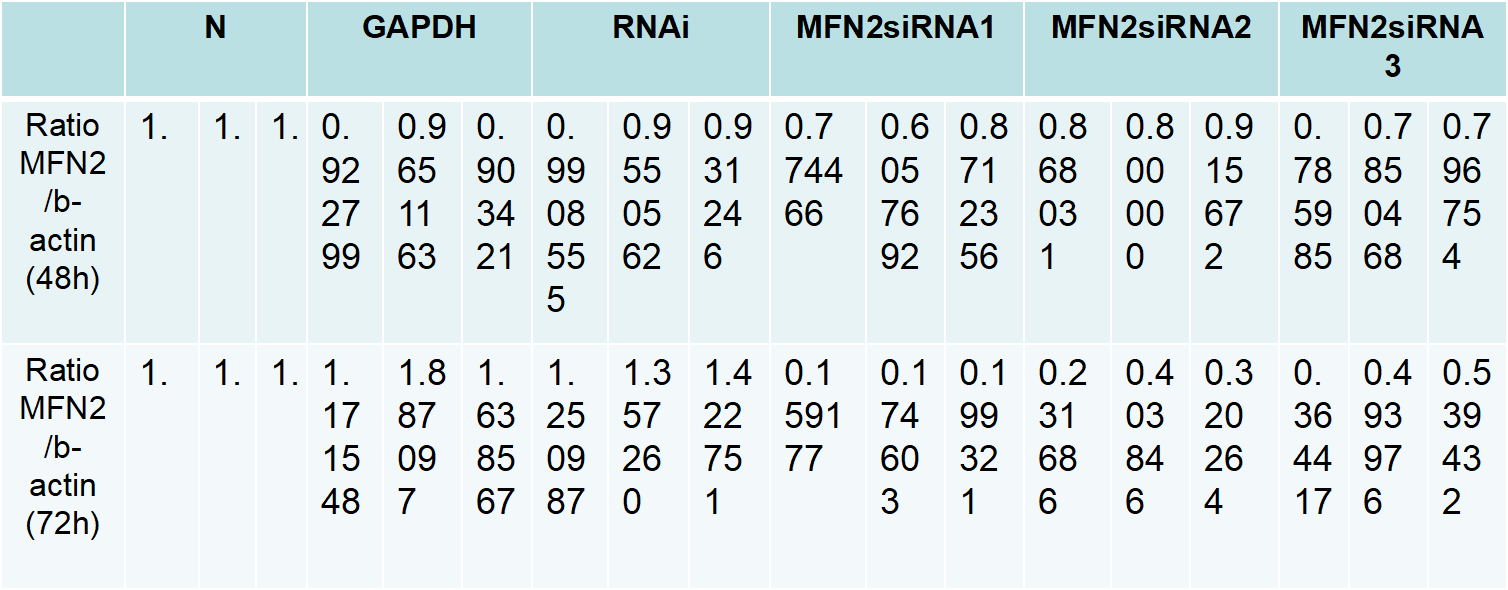

Supplement: S2 File — Raw data for Fig 1B, Fig 2A and 2B, Fig 3A, 3B, 3C and 3D, Fig 4B–4F, Fig 5A and 5B. (ZIP) [file pone.0213877.s002.zip › s8 Fig4C.tif]

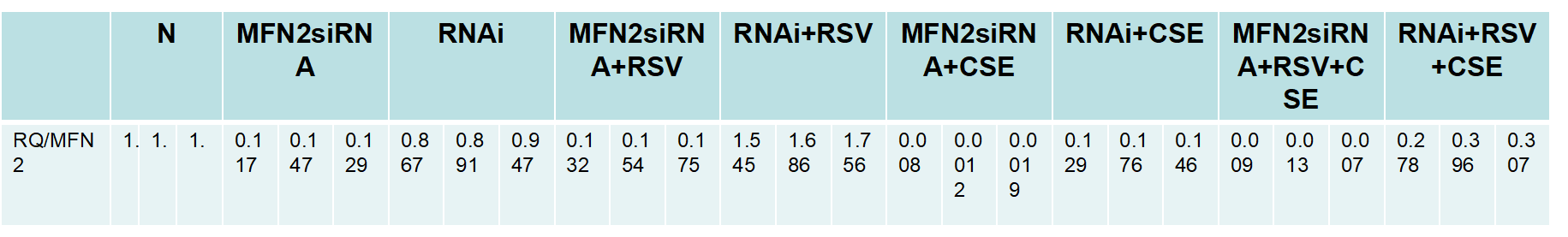

Supplement: S2 File — Raw data for Fig 1B, Fig 2A and 2B, Fig 3A, 3B, 3C and 3D, Fig 4B–4F, Fig 5A and 5B. (ZIP) [file pone.0213877.s002.zip › s9 Fig4D.tif]
